# Identification and distribution of gene clusters required for synthesis of sphingolipid metabolism inhibitors in diverse species of the filamentous fungus *Fusarium*

**DOI:** 10.1186/s12864-020-06896-1

**Published:** 2020-07-23

**Authors:** Hye-Seon Kim, Jessica M. Lohmar, Mark Busman, Daren W. Brown, Todd A. Naumann, Hege H. Divon, Silvio Uhlig, Robert H. Proctor

**Affiliations:** 1 U. S. Department of Agriculture, Agriculture Research Service, National Center for Agricultural Utilization Research, Peoria, IL USA; 2 Norwegian Veterinary Institute, Oslo, Norway

**Keywords:** *Fusarium*, Genome sequence, Secondary metabolite, Sphinganine analog, Biosynthetic gene cluster, Horizontal gene transfer

## Abstract

**Background:**

Sphingolipids are structural components and signaling molecules in eukaryotic membranes, and many organisms produce compounds that inhibit sphingolipid metabolism. Some of the inhibitors are structurally similar to the sphingolipid biosynthetic intermediate sphinganine and are referred to as sphinganine-analog metabolites (SAMs). The mycotoxins fumonisins, which are frequent contaminants in maize, are one family of SAMs. Due to food and feed safety concerns, fumonisin biosynthesis has been investigated extensively, including characterization of the fumonisin biosynthetic gene cluster in the agriculturally important fungi *Aspergillus* and *Fusarium*. Production of several other SAMs has also been reported in fungi, but there is almost no information on their biosynthesis. There is also little information on how widely SAM production occurs in fungi or on the extent of structural variation of fungal SAMs.

**Results:**

Using fumonisin biosynthesis as a model, we predicted that SAM biosynthetic gene clusters in fungi should include a polyketide synthase (PKS), an aminotransferase and a dehydrogenase gene. Surveys of genome sequences identified five putative clusters with this three-gene combination in 92 of 186 *Fusarium* species examined. Collectively, the putative SAM clusters were distributed widely but discontinuously among the species. We propose that the SAM5 cluster confers production of a previously reported *Fusarium* SAM, 2-amino-14,16-dimethyloctadecan-3-ol (AOD), based on the occurrence of AOD production only in species with the cluster and on deletion analysis of the SAM5 cluster PKS gene. We also identified SAM clusters in 24 species of other fungal genera, and propose that one of the clusters confers production of sphingofungin, a previously reported *Aspergillus* SAM.

**Conclusion:**

Our results provide a genomics approach to identify novel SAM biosynthetic gene clusters in fungi, which should in turn contribute to identification of novel SAMs with applications in medicine and other fields. Information about novel SAMs could also provide insights into the role of SAMs in the ecology of fungi. Such insights have potential to contribute to strategies to reduce fumonisin contamination in crops and to control crop diseases caused by SAM-producing fungi.

## Background

Sphingolipids play critical roles as structural components and signaling molecules in eukaryotic membranes. As a result, aberrations in sphingolipid content in plant and animal cells can lead to disease [1–3]. Given the biological roles of sphingolipids, their metabolism is a likely target during interactions of organisms with pathogens, competitors, hosts and/or predators. This is evident by production in some organisms of secondary metabolites that inhibit sphingolipid metabolism [4, 5]. Some of these inhibitors target biosynthesis of dihydroceramides, structurally simple sphingolipids from which more complex sphingolipids are derived. Dihydroceramide biosynthesis begins when an aminotransferase (serine palmitoyltransferase) catalyzes condensation of serine with the CoA-activated fatty acid palmitate to form 3-ketosphinganine [2]. A dehydrogenase (3-ketosphinganine reductase) then reduces the 3-keto group to a hydroxyl to form sphinganine. Finally, an acyltransferase (sphinganine-*N*-acyltransferase) catalyzes condensation of sphinganine with a fatty acid to form dihydroceramides.

**Fig. 1:**
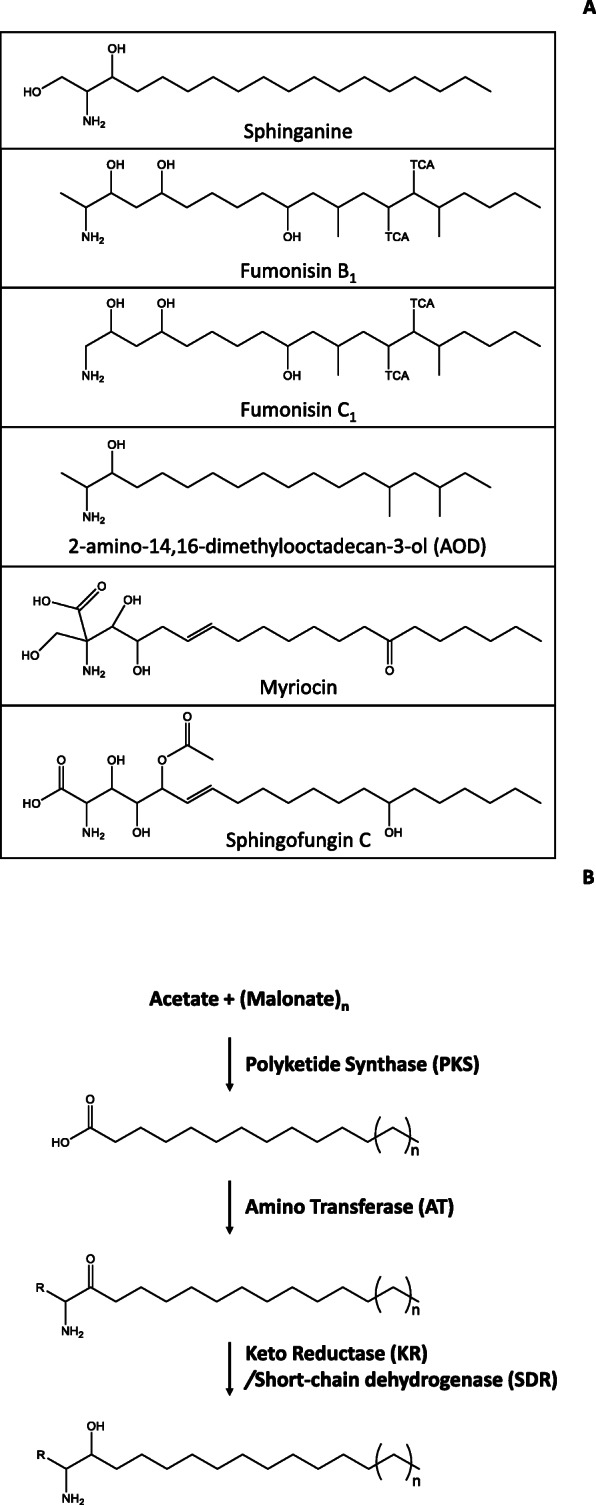
(**a**) Comparison of chemical structures of sphinganine, fumonisins, sphingofungin, and 2-amino-14, 16-dimethyl-octadecan-3-ol (AOD). (**b**) the three biosynthetic steps that are predicted to be required for formation of sphinganine-analog metabolites (SAM) and the enzymes required for catalysis of the biochemical reactions

Some sphingolipid metabolism inhibitors are structural analogs of sphinganine [4, 5], and will hereafter be referred to as sphinganine-analog metabolites (SAMs). Several fungal secondary metabolites have been demonstrated or are presumed to be SAMs. These include AAL toxins produced by *Alternaria arborescens* [6, 7], 2-amino-14,16-dimethyl-octadecan-3-ol (AOD) produced by *Fusarium avenaceum* [8], australifungin produced by *Sporormiella australis* [9], myriocin produced by *Isaria sinclairii* [10], sphingofungins produced by *Aspergillus fumigatus* [11, 12], viridiofungins produced by *Trichoderma viride* [13]*,* and fumonisins produced by some species of *Aspergillus*, *Fusarium* and *Tolypocladium* [14–18]. Like sphinganine, SAMs consist of a linear carbon chain with an amine group near one end and a hydroxyl group on the nonterminal carbon adjacent to the amine (Fig. 1a). Most SAMs differ in structure from sphinganine by the presence of methyl, hydroxyl or other substituents on the carbon chain. The mechanism by which some fungal SAMs disrupt sphingolipid metabolism has been determined. For example, sphingofungin C and myriocin inhibit serine palmitoyltransferase, while fumonisins and australifungin inhibit sphinganine-*N*-acyltransferase [3].

Fumonisins are among the mycotoxins of most concern because of their association with multiple human and livestock diseases combined with their frequent occurrence in maize and other crops. In some communities in which maize is a food staple, consumption of fumonisin contaminated maize is epidemiologically correlated with esophageal cancer and neural tube defects in humans [19, 20]. Animal diseases caused by fumonisins include leukoencephalomalacia in horses, pulmonary edema in swine, and cancer in laboratory rodents [21]. Contamination of maize with fumonisins has been attributed primarily to the ear rot pathogen *Fusarium verticillioides* and to a lesser extent to *F. proliferatum* [22, 23]. Since the discovery of fumonisins three decades ago, many biochemical and genetic details of their biosynthesis in *F. verticillioides* have been elucidated with the aim of developing strategies to reduce fumonisin contamination in crops.

Most fumonisin-producing species of *Fusarium* that have been examined produce the fumonisin analogs B_1_, B_2_, B_3_ and B_4_ (FB_1_, FB_2_, FB_3_ and FB_4_) in greatest abundance [17, 24, 25]. The first committed step in FB biosynthesis is formation of a linear, fully-saturated, 18-carbon-long chain with methyl groups at carbon atoms 12 and 16 (C12 and C16). Formation of this molecule is catalyzed by a polyketide synthase (PKS). The next two steps in fumonisin biosynthesis mirror sphinganine biosynthesis. First, an aminotransferase (AT) catalyzes condensation of the polyketide with alanine to form a linear 20-carbon chain with the two methyl groups, an amine group at C2, and a keto group at C3 [26]. Second, a short-chain dehydrogenase reductase (SDR) catalyzes reduction of the C3 keto group to a hydroxyl [27]. Formation of FB_1_, FB_2_, FB_3_ and FB_4_ results from subsequent hydroxylation of the polyketide backbone at two to five positions (C4, C5, C10, C14, C15) and esterification of tricarboxylate molecules to the hydroxyls at C14 and C15 [25]. Several *Fusarium* species produce C-series fumonisins (FCs) in greatest abundance rather than FBs [18, 28]. The two series differ in structure by the presence (FBs) or absence (FCs) of a terminal methyl group adjacent to the amine. This structural difference results from condensation of the precursor polyketide with different amino acids: alanine in FB biosynthesis, and glycine in FC biosynthesis [26, 29]. Which amino acid is used in biosynthesis is determined by the amino acid substrate specificity of the AT (Fum8) in FB versus FC-producing species [26].

There is almost no information on the biosynthesis of fungal SAMs other than fumonisins, and whether there are fungal SAMs in addition to those that have already been identified is not known. Further, there is little information on the distribution of SAM production among fungal species. Analyses of the distribution of fumonisin biosynthetic (*FUM*) genes and fumonisin production indicate that although both are discontinuously distributed among *Fusarium* species, they occur with the highest frequency among members of the multispecies lineage known as the *Fusarium fujikuroi* species complex [18, 30]. In *Aspergillus*, *FUM* genes and fumonisin production occur in certain species in the multispecies lineage known as section *Nigri* [31]. Because no other SAM biosynthetic genes have been described in fungi, it is unclear whether other SAMs have similar distribution patterns. A limitation in assessments of production of fungal secondary metabolites is that production of a given metabolite can occur only under a limited set of conditions [32, 33]. In contrast, assessing presence of secondary metabolite biosynthetic genes (i.e., genetic potential for production) is unaffected by environmental conditions. Except for fumonisins, however, assessments of genetic potential for SAM production in fungi are hindered by the lack of information on SAM biosynthetic genes.

The objective of the current study was to identify SAM biosynthetic genes in order to investigate the potential distribution of SAM production in fungi. Our approach to identify the genes was based on the tendency of fungal secondary metabolite biosynthetic genes to be clustered [32, 34, 35] and the hypothesis that SAM biosynthesis should mirror the biosynthesis of both sphinganine and fumonisins. We focused our initial efforts on the genus *Fusarium*, because of the availability of genome sequences for 186 *Fusarium* species (fusaria) provided an opportunity for an in-depth survey of SAM clusters in an agriculturally important genus. Our survey of *Fusarium* genomes identified five lineages of putative SAM biosynthetic gene clusters that were distinct from each other and from the fumonisin cluster. We also identified putative SAM clusters in species of 24 other fungal genera. Our results provide a genomics approach that could lead to identification of novel SAM structures with applications in medicine and other fields [36–38]. Enhancing knowledge of fungal SAMs could provide insights into their ecological roles, which in turn has potential to contribute to development of strategies that reduce fumonisin contamination and crop diseases caused by SAM-producing fungi.

## Results

### SAM cluster identification

Our approach to assess the genetic potential for SAM production in fungi was based on two concepts. First, enzyme-encoding genes directly involved in biosynthesis of the same secondary metabolite tend to be clustered in fungi [34, 35]. Second, we postulated that biosynthesis of novel fungal SAMs should mirror fumonisin biosynthesis, which in turn mirrors sphinganine biosynthesis, except that the former uses a secondary metabolic enzyme (PKS) and the latter uses an enzyme (fatty acid synthase) more often associated with primary metabolism [39, 40] for formation of the linear carbon backbone. If our postulate is correct, fungal SAM biosynthetic gene clusters should include genes encoding: 1) a PKS to catalyze synthesis of a highly reduced, linear carbon chain; 2) an AT to catalyze condensation of the polyketide with an amino acid; and 3) an SDR to catalyze reduction of the polyketide-derived keto group to a hydroxyl (Fig. 1b).

**Fig. 2:**
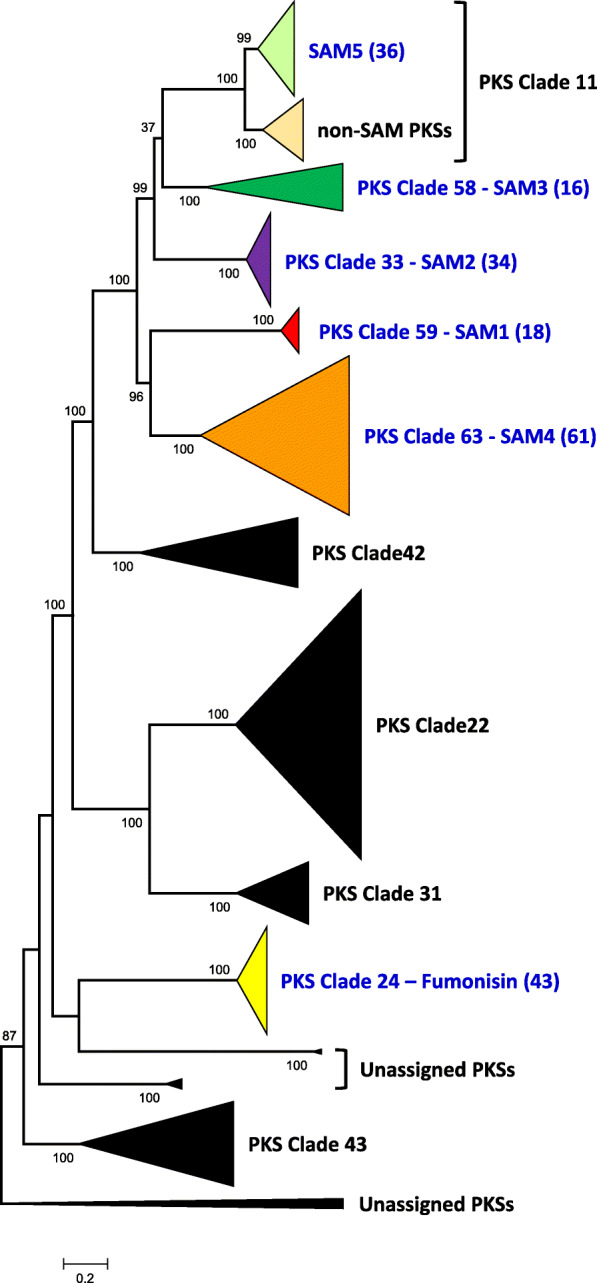
Phylogenetic tree of *Fusarium* PKS genes retrieved by OrthoFinder analysis of *Fusarium* genome sequence data and based on their relatively close relationships to the fumonisin PKS gene (*FUM1*). The phylogenetic tree was inferred by maximum likelihood analysis of an alignment of predicted amino acid sequences of coding regions of 347 PKS gene homologs. All clades shown (indicated by triangles) are within the larger, previously described Reducing PKS Clade III, and the PKS clade numbers correspond to previously described clade numbers [41]. The colored triangles indicate clades consisting of homologs located in putative SAM clusters, and black triangles indicate clades consisting of homologs that are not located in SAM clusters. For clades of SAM PKS genes, numbers in parentheses indicate number of homologs per clade. Numbers near branches are bootstrap values based on 1000 pseudoreplicates

BLAST and OrthoFinder analyses of predicted proteins from 343 genome sequences representing 186 *Fusarium* species identified 379 PKS genes that were closely related to the fumonisin PKS gene based on the branching pattern in the Orthofinder-inferred tree. In subsequent maximum likelihood analysis using IQ-Tree, all but eight of these PKSs were resolved as members of 10 clades, or orthologous groups, described in previous phylogenetic analyses of *Fusarium* PKS genes [41, 42]. These 10 PKS clades grouped within Reducing PKS Clade III (Fig. 2), a lineage of PKSs that synthesize or are predicted to synthesize linear, fully reduced polyketides [41]. We used both manual assessments and antiSMASH analysis to determine which of the 379 PKS genes were located in putative gene clusters that also included AT and SDR genes. This analysis revealed that PKS genes resolved into PKS Clades 33, 58, 59, and 63 as well as PKS Clade 24 (fumonisin PKS) were located in putative clusters that included an AT gene, and in all but eight cases an SDR gene. In addition, approximately half of the PKS genes in PKS Clade 11 were located in a putative cluster that included an AT and SDR gene. Putative clusters with this three-gene combination were sorted into orthologous groups using phylogenetic trees inferred separately from the predicted amino acid sequences of the PKS, AT and SDR genes. Although there were some topological differences between the resulting PKS and AT trees, the two trees were similar in that PKS genes resolved in the same clade were from gene clusters that included AT genes that were also resolved in the same clade (Additional file 1).

**Fig. 3:**
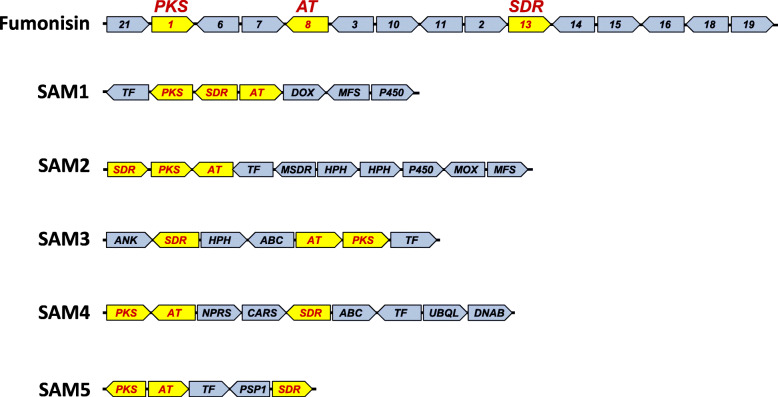
Organization of genes in the fumonisin biosynthetic gene cluster and five putative novel SAM biosynthetic gene clusters (clusters SAM1 – SAM5) identified in *Fusarium* genomes. Yellow arrows indicate the PKS, AT and SDR genes in each cluster, while the blue arrows indicate other genes in the clusters. The arrows point in the direction of transcription. For the fumonisin cluster, numbers in arrows correspond to the *FUM* gene number (e.g., *21* indicates gene *FUM21*). For the SAM1 – SAM5 clusters, the letters within blue arrows indicate the predicted functions of the genes, based on sequence homology of predicted proteins: TF, transcription factor; DOX, dioxygenase; MFS, major facilitator superfamily transporter; P450, cytochrome P450 monooxygenase; MSDR, mannitol 2-dehydrogenase; HPH, hypothetical protein (no function predicted); MOX, FAD-binding monooxygenase; ANK, ankyrin protein; ABC, ABC transporter; NRPS, nonribosomal peptide synthetase; CARS, carnosine synthase 1; UBQL, ubiquitin ligase; DNAB, acetate regulatory DNA-binding protein; PSP1, parasitic phase-specific protein

**Fig. 4:**
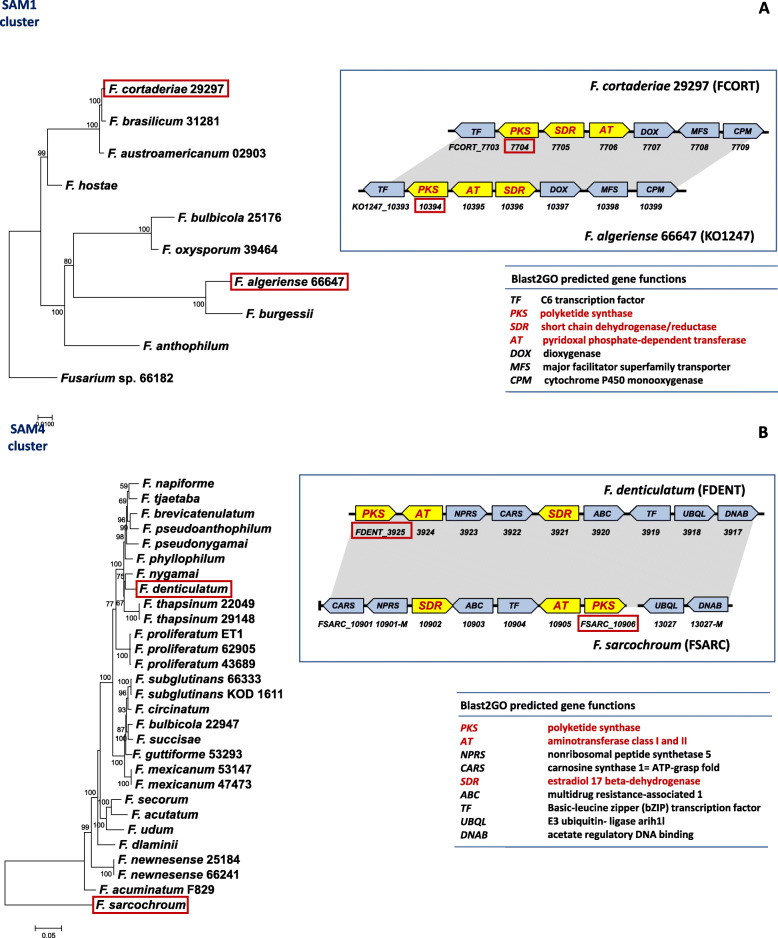
Comparison of the SAM1 (**a**) and SAM4 (**b**) cluster homologs in the species/strains of *Fusarium* in which they occur. The phylogenetic trees on the left were inferred by maximum likelihood analysis of alignments of nucleotide sequences of the PKS-gene coding regions from the respective clusters. Numbers near branches are bootstrap values based on 1000 pseudoreplicates. The diagram to the upper right depicts two different arrangements of genes in each cluster type. Yellow arrows indicate PKS, AT and SDR genes; and blue arrows indicate genes that encode other types of proteins. The arrows point in the direction of transcription. Numbers below arrows are locus tag numbers for the two species. Prefixes for locus tag number of genes for all species are shown in Additional file 3. The table on the bottom right indicates the functions of genes in the cluster based on sequence homology to genes of known function. Numbers after species names indicate strain numbers when more than one strain of a species was included in this study. Five-digit strain numbers without letters indicate NRRL strains

Comparisons of the gene content of the putative clusters using results of Blast2GO analysis revealed five distinct groups of clusters (Figs. 3 and 4, and Additional file 2). These five groups were consistent with the phylogenetic trees in that PKS and AT orthologs that were resolved in the same clade in the respective trees were located in gene clusters with the same or nearly the same gene content. We propose that the five groups of clusters are SAM biosynthetic gene clusters and will hereafter refer to them as the SAM1 – SAM5 clusters. In some cases, genes at the ends of antiSMASH-predicted clusters were not present in all orthologs of a cluster. Based on their absence in some species, we postulated that such genes were not part of the clusters and removed them from subsequent analyses. In addition, homologs of SAM cluster genes were located on different contigs in some species. This occurred at the highest frequency for homologs of the SAM2, SAM3 and fumonisin cluster genes (Additional file 3). We propose that in most cases dispersal of SAM cluster genes on multiple contigs is an artifact of genome sequence quality and/or assembly. This rationale is based on the highly fragmented nature of some genome sequences in which the SAM genes occurred on different contigs (Additional file 4). Further, although fumonisin cluster genes were dispersed on multiple contigs in some genome sequences, clustering of the genes is well-documented in multiple species of both *Fusarium* and *Aspergillus* [15, 33]. Because of evidence for dispersal of some other secondary metabolite gene clusters to multiple loci [43], further analyses (e.g., with higher quality sequence data) are required to confirm clustering of the SAM genes that were observed on different contigs in this study.

The approach described above identified 208 putative SAM cluster homologs, including fumonisin cluster homologs, from among the 343 genome sequences analyzed: 18 SAM1 cluster homologs, 34 SAM2 homologs, 16 SAM3 homologs, 61 SAM4 homologs, 36 SAM5 homologs, and 43 fumonisin cluster homologs. We also identified eight cluster homologs that had the same gene content as the SAM3 cluster except that they lacked an SDR gene (i.e., the SDR gene was either absent or truncated) (Additional file 3). Because of their similarity to SAM3 cluster homologs, we included these eight clusters in subsequent analyses of the SAM3 cluster.

### Distribution of putative SAM clusters

To assess the distribution of putative SAM clusters across the known breadth of phylogenetic diversity of *Fusarium*, we first inferred a species tree using 13 housekeeping genes mined from the same *Fusarium* genome sequences surveyed for SAM clusters. We then mapped the presence and absence of each SAM cluster to each species in the tree. Previous studies have resolved *Fusarium* into 23 multispecies lineages, known as species complexes, and four single-species lineages [44–47]. For brevity in this study, we have shortened species complex names; e.g., the *F. fujikuroi* and *F. sambucinum* species complexes have been abbreviated as the Fujikuroi and Sambucinum complexes, respectively.

**Fig. 5:**
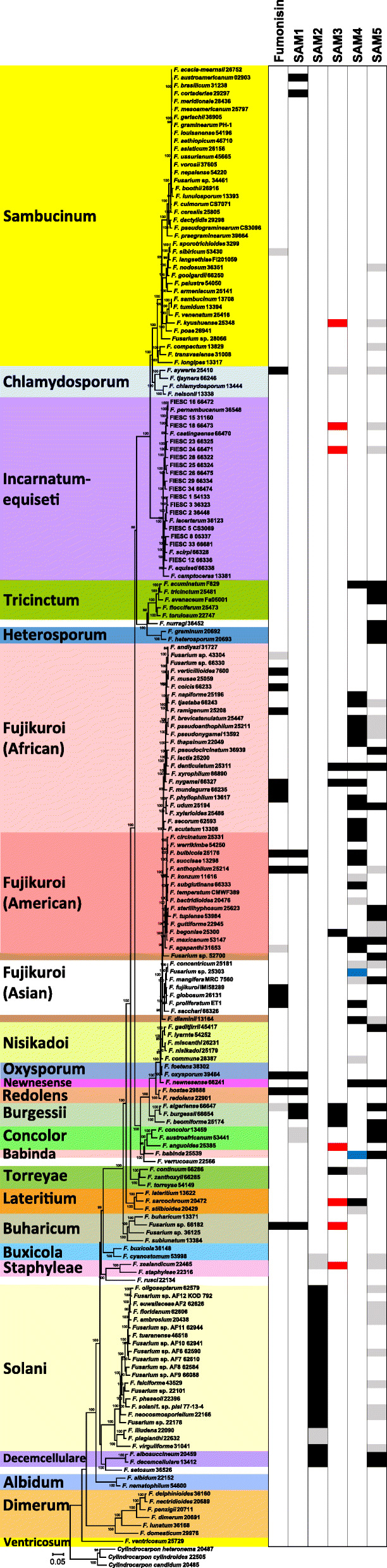
Distribution of the putative SAM1 – SAM5 clusters and the fumonisin cluster among 186 *Fusarium* species. The phylogenetic tree to the left is a species tree, and the boxes on the right indicate the presence and absence of each cluster in individual species. The species tree was inferred from concatenated alignments of coding regions of 13 housekeeping genes (Additional file 11). *Fusarium* species complexes are indicated with different colored boxes. The three previously described clades (African, American and Asian) within the *F. fujikuroi* complex are indicated with different shades of pink. Two single-species lineages (*F. dlaminii* and *Fusarium* sp. NRRL 52700) within the *F. fujikuroi* complex are indicated with brown boxes. For each cluster, a black box indicates the cluster is present; a red box indicates > 70% of cluster genes are present; a blue box indicates the PKS and AT genes are pseudogenized; gray indicates that < 50% cluster genes are present; and white indicates the cluster is absent

In the species tree inferred in the current study, taxa were resolved into exclusive and well-supported clades that corresponded to the 23 previously described *Fusarium* species complexes (Fig. 5). All species previously reported to be members of the same species complex were resolved within the same clade in the species tree. In addition, the Fujikuroi complex, which was the most deeply sampled complex in the current study, was further resolved into three well-supported clades that were consistent with the previously described African, American and Asian clades of the complex [48]. Also consistent with previous studies, *F. nurragi*, *F. rusci*, *F. setosum* and *F. verrucosum* were not nested within any of the species complexes in the species tree (Fig. 5). The relationships of most species complexes to one another were also consistent with previous studies. However, relationships of some of the earliest diverging complexes (e.g., Albidum, Decemcellulare, Dimerum, Solani and Staphyleae complexes) to one another differed from some previously reported species trees [46]. In addition, relationships of some species within some species complexes were not identical to previous studies. A notable example of this was the position of *F. dlaminii* within the Fujikuroi complex. In previous studies, this species was often resolved as a basal lineage of the African clade, but in the species tree in the current study, *F. dlaminii* was the most basal lineage of the entire Fujikuroi complex (Fig. 5). Nevertheless, given its general similarities to previously reported *Fusarium* species trees, we deemed the species tree inferred in the current study as a reasonable estimation of phylogenetic relationships that exist within *Fusarium*.

Mapping the presence and absence of the SAM1 – SAM5 and fumonisin clusters to taxa in the species tree revealed that each cluster had a unique pattern of distribution. In most cases the distribution was discontinuous in that closely related species often differed in the presence and absence of a given cluster, whereas some distantly related species had the same cluster (Fig. 5, Additional file 3). Each cluster was detected in 16–61 genomes representing 10–34 species and 2–12 species complexes. SAM3 was detected in the fewest genomes (16), but these genomes represented 15 species and six species complexes (Sambucinum, Fujikuroi, Oxysporum, Redolens, Burgessii and Buharicum complexes). In contrast, SAM4 was detected in the most genomes (61), which represented 33 species and five species complexes (Tricinctum, Fujikuroi, Newnesense, Babinda and Lateritium complexes). The SAM2 cluster was unique in that it exhibited an almost continuous distribution pattern in the Decemcellulare and Solani complexes. The SAM5 cluster was detected in 36 *Fusarium* genomes representing 28 species and 12 species complexes (Tricinctum, Heterosporum, Newnesense, Burgessii, Concolor, Babinda, Decemcellulare). One species (*F. nurragi*) with the SAM5 cluster has not been assigned to a species complex. Like other SAM clusters, the fumonisin cluster also exhibited a discontinuous distribution (Fig. 5), which has been described previously [18]. However, as far as we are aware, this is the first report of the occurrence of the fumonisin cluster in members of the Chlamydosporum (*F. aywerte*), Redolens (*F. hostae*) and Buharicum (*Fusarium* sp. 66182) complexes.

Sixty-nine of the *Fusarium* species examined in this study had at least one SAM cluster, including the fumonisin cluster (Fig. 5, Additional files 3 and 4). Fifteen of the 69 species had two SAM clusters, and five species had three SAM clusters. None of the species examined had more than three functional SAM clusters. However, *F. algeriense* had orthologs of the SAM1, SAM3 and SAM5 clusters as well as a degenerate ortholog of the fumonisin cluster, which lacked multiple genes essential for fumonisin biosynthesis. The SAM4 and SAM5 clusters as well as the SAM1 and fumonisin clusters co-occurred in five species, more often than any other two-cluster combination. The three-cluster combination of the SAM1, SAM3 and fumonisin clusters occurred in two species: *F. bulbicola* and *Fusarium* sp. 66182. The co-occurrence of three other SAM clusters was detected in three other species, but each of these species had a different combination of clusters (Fig. 5, Additional file 4). The co-occurrence of two or three SAM clusters was observed in multiple species complexes. For example, the co-occurrence of the SAM1 and fumonisin clusters was observed in the Fujikuroi, Oxysporum, Redolens and Buharicum species complexes, while the co-occurrence of the SAM1, SAM3 and fumonisin clusters occurred in the Fujikuroi and Buharicum complexes.

To our knowledge, AOD is the only SAM other than fumonisins that has been reported to be produced by a species of *Fusarium*, namely *F. avenaceum*. Examination of the distribution of SAM clusters indicated that the SAM5 cluster is the only SAM cluster present in the genome sequences of the *F. avenaceum* strains examined (Fig. 5, Additional file 3). Therefore, the SAM5 cluster is likely to be the AOD biosynthetic gene cluster.

### Contributors to discontinuous distribution of SAM clusters

The discontinuous distribution of the SAM clusters in *Fusarium* indicates that processes other than uniform vertical inheritance of the clusters has occurred during the evolutionary divergence of species. Two processes that could have contributed to the distribution are gene loss and horizontal gene transfer (HGT). That gene loss has occurred through deletion is suggested by the presence of partial SAM clusters in which two or more of the cluster genes were absent. Forty-four of the species examined in this study had partial SAM clusters in which 50% or more of the genes that occur in an intact cluster were absent (Fig. 5). Analysis of partial SAM1-SAM4 clusters indicated that in some of them one or more cluster genes had undergone pseudogenization. For example, the PKS, AT and SDR genes were pseudogenized in the SAM4 cluster orthologs in *F. babinda* and *Fusarium* sp. 25303 (Additional file 3). In some cases, individual species exhibited variation in the presence and absence of genes in a given cluster. For example, two *F. bulbicola* strains (NRRL 13618 and 25176) had an intact SAM1 cluster, while a third *F. bulbicola* strain (NRRL 22947) lacked all SAM1 cluster genes. Conversely, the fumonisin cluster was intact in strain NRRL 22947, but in the other two *F. bulbicola* strains, the fumonisin PKS and AT genes as well as a third gene (*FUM14*) essential for fumonisin biosynthesis were pseudogenized; i.e., the coding regions of the genes had mutations that resulted in one or more internal stop codons. We detected intact SAM5 clusters in 23 species, and apparent partial SAM5 clusters in 26 species. However, further examination of the apparent partial clusters revealed that they included a homolog of the PKS gene but no other SAM5 cluster genes. In phylogenetic trees inferred from SAM5 PKS homologs, homologs from apparent partial clusters formed a well-supported clade that was distinct from a well-supported clade formed by homologs from intact SAM5 clusters. In Fig. 2, these clades are labelled non-SAM PKSs and SAM5, respectively. These findings indicate that the apparent partial SAM5 cluster was more likely a non-SAM cluster that included a PKS gene that was closely related to but distinct from the SAM5 PKS gene.

We used previously described approaches [18, 49] to assess the potential contribution of HGT to the discontinuous distribution of SAM clusters among *Fusarium* species. The approaches were: 1) reconciliation analysis with the program NOTUNG, which infers HGT, duplication or loss of genes to reconcile branch conflicts between gene trees and species trees; 2) constraint analysis with the Shimodaira-Hasegawa and Approximately Unbiased (SH-AU) tests; and 3) estimates of synonymous site divergence. For these assessments, each SAM cluster was analyzed separately, and the analyses employed nucleotide sequence alignments and trees inferred from orthologs of the PKS and AT genes, as well as the SDR gene in analyses of the SAM1 and SAM2 clusters. For NOTUNG reconciliation analysis, we employed a species tree inferred from 13 housekeeping genes from a subset of 83 species (96 isolates) that had one or more SAM clusters (Additional file 5). The NOTUNG analysis inferred 6–11 HGT events among *Fusarium* species per cluster for the SAM1 and SAM3 – SAM5 clusters, but no HGT events for the SAM2 cluster (Tables 1 and 2, and Additional file 5). We used SH-AU tests to further assess the likelihood of NOTUNG-inferred HGT events. To do so, we generated SAM gene trees in which individual branches that conflicted with the species tree were constrained to match the species tree. We then used SH-AU tests to determine whether each constrained tree had a lower likelihood value than the unconstrained tree. The results of the SH-AU tests supported some but not all the NOTUNG-inferred HGT events (Tables 1 and 2). The lower number of HGT events supported by the SH-AU tests resulted in part because it was not possible to examine four of the NOTUNG-inferred HTG events by constraint analysis.

**Table 1:** Summary of evidence for horizontal gene transfer (HGT) events of SAM clusters among *Fusarium* species. Values in each column indicate numbers of transfer events supported by results of each analysis: NOTUNG reconciliation, constraint analysis using Shimodaira-Hasegawa and Approximately Unbiased (SH-AU) tests, and synonymous site divergence analysis

Cluster	NOTUNG	SH-AU^**a**^	Synonymous Site Divergence (***d***_***S***_)^**b**^
SAM1	6	4	3 (5)
SAM2	0	0	0
SAM3	7	6	7
SAM4	11	3	3
SAM5	6	4	2
Total	30	17	15 (17)

^a^ Results of the SH-AU tests were considered to be consistent with horizontal transfer when P values from both tests were < 0.01. Given tree topologies, we were not able to assess some of the NOTUNG-inferred HGT events with the constraint/SH-AU analysis. Thus, one event for SAM3 genes, two events for SAM4 genes, and one event for SAM5 genes were not assessed by constraint/SH-AU analysis

^b^ Except those within parentheses, values indicate the number of NOTUNG-inferred transfer events that were consistent with *d*_*S*_ values for SAM genes being less than the *d*_*S*_ values for housekeeping genes (ratio of *d*_*S*_ SAM genes to *d*_*S*_ housekeeping genes, *d*_*S*_ ratio, < 1.0). For SAM1, the value within parentheses indicates transfer events consistent with a *d*_*S*_ ratio of 1.0–1.5 (see Methods)

**Table 2:** Examples of putative horizontal gene transfer events of SAM clusters among species of *Fusarium* inferred initially by NOTUNG reconciliation analysis and supported (+) or not supported (−) by constraint analysis using Shimodaira-Hasegawa and Approximately Unbiased (SH-AU) tests and by synonymous site divergence analysis

Cluster	Potential Donor	Potential Recipient	NOTUNG	SH-AU^**a**^	Synonymous Site Divergence^**b**^
SAM1	*F. hostae*	*F. austroamericanum*-*F. brasilicum-F. cortaderia*	+	+	+
SAM1	*F. hostae*	*Fusarium* sp. 66182	+	nt	+
SAM1	*F. bulbicola*	*F. oxysporum*	+	+	+
SAM3	*F. burgessii*	FIESC18-FIESC24-*F. kyushuense*	+	+	+
SAM3	*F. denticulatum*	*F. begonia*	+	nt	+
SAM4	*F. pseudonygamai*	*F. phyllophilum*	+	+	+
SAM4	*F. newnesense*	*F. acuminatum*	+	nt	+
SAM4	*F. subglutinans*	*F. circinatum*	+	–	+
SAM5	*F. denticulatum*-*F. pseudcircinatum*- *F. udum*	*F. mangiferae*	+	+	+
SAM5	*F. newnesense*	*F. concolor*	+	+	+
SAM5	*F. tupiense*	*F. mexicanum*	+	–	–

^a^ Results of the SH-AU tests were considered to be consistent with horizontal transfer only when *P* values from both tests were < 0.01. nt indicates that, given the tree topology in question, it was not possible to test the branch conflict indicative of the transfer event

^b^ Results of the *d*_*S*_ analysis were considered to be consistent with horizontal transfer when, for a given pairwise combination of taxa, the *d*_*S*_ value for SAM genes was less than *d*_*S*_ for housekeeping genes

The use of estimates of number of synonymous changes per synonymous site (*d*_*S*_) to obtain further evidence of HGT events is based on the idea that divergence levels of horizontally transferred genes should reflect the length of time since transfer, whereas divergence of vertically inherited genes should reflect the length of time since speciation [18]. Because HGT would occur after speciation, *d*_*S*_ values for transferred genes are expected to be less than values for vertically inherited genes. We assumed that individual housekeeping genes that yield trees that mirror the species tree are likely to have been vertically inherited. Examination of *d*_*S*_ values provided evidence for multiple HGT events; i.e., in multiple pairwise comparisons of species, *d*_*S*_ values for SAM genes were less than *d*_*S*_ values for housekeeping genes. *d*_*S*_ values for SAM1, SAM4 and SAM5 genes supported only some of the NOTUNG-inferred HGT events, whereas *d*_*S*_ values for SAM3 genes provided support for all the NOTUNG-inferred HGT events (Tables 1 and 2; Additional files 5 and 6). Together, the NOTUNG, SH-AU, and synonymous site divergence analyses provided evidence for 15–30 HGT events of SAM clusters among *Fusarium* species. This in turn is consistent with the hypothesis that HGT has contributed to the distribution of SAM clusters in *Fusarium*.

### SAM clusters in other fungal genera

For an initial assessment of the occurrence of SAM clusters in fungi other than *Fusarium* (non-*Fusarium*), we used predicted protein sequences of representative PKS and AT genes from the *Fusarium* SAM1 – SAM5 and fumonisin clusters as queries in BLASTp analysis of the NCBI-NR fungal protein sequence database. From the top 100 hits obtained from both queries, we determined which of the corresponding PKS and AT genes from the same organism were located on the same chromosome and within five genes from one another. The genome sequences from these organisms were then subjected to antiSMASH analysis to further assess the gene content of the putative gene clusters. Our approach identified putative SAM clusters in 43 fungal species. However, only 26 of the clusters included an SDR gene. The 43 non-*Fusarium* species with putative SAM clusters were from 24 genera distributed among the classes Dothideomycetes, Eurotiomycetes, Lecanoromycetes and Sordariomycetes of the Ascomycota (Additional file 7). In phylogenetic trees inferred from separate analyses of the deduced amino acid sequences of the PKS, AT and SDR genes in the putative SAM clusters, the non-*Fusarium* sequences were generally resolved in clades that included the *Fusarium* SAM PKS, AT or SDR genes (Additional file 1). Within each of these clades, *Fusarium* sequences were monophyletic, and non-*Fusarium* sequences were sister or basal to the *Fusarium* sequences. The majority of non-*Fusarium* PKSs (20) resolved with the *Fusarium* SAM1 PKS clade. Nine non-*Fusarium* PKSs resolved with SAM3, and nine with SAM4, whereas only two non-*Fusarium* PKSs resolved with SAM2 and SAM5.

In phylogenetic trees inferred from PKS, AT or SDR sequences from each of the five SAM clades, sequences from the same genus tended to be monophyletic. Sequences from the same class were monophyletic in some cases but not in others. For example, in the SAM1 PKS tree, sequences from genera of the Eurotiomycetes (*Arthroderma*, *Aspergillus*, *Coccidioides*, *Penicillium* and *Trichophyton*) were monophyletic, whereas sequences from the three genera of the Sordariomycetes (*Fusarium*, *Hypoxylon* and *Metarhizium*) were not monophyletic (Additional file 1).

Examination of the gene content of SAM clusters suggested that metabolic products of some *Fusarium* and non-*Fusarium* clusters with PKS and AT in the same clade may be structurally different. For example, the *Metarhizium* SAM1-like cluster was most closely related to the *Fusarium* SAM1 cluster according to the phylogenetic trees. However, in addition to the PKS, AT and SDR gene, the *Fusarium* cluster included a dioxygenase and a cytochrome P450 monooxygenase gene, whereas the *Metarhizium* cluster included the dioxygenase gene but not the monooxygenase gene (Additional file 7). This suggests that synthesis of the metabolic product of the *Metarhizium* SAM1-like cluster included at least one less oxygenation reaction than synthesis of the metabolic product of the *Fusarium* SAM1 cluster. The *A. fumigatus* SAM1-like cluster included both dioxygenase and monooxygenase genes, but also one to three dehydrogenase genes in addition to the SDR gene. *A. fumigatus* is reported to produce the SAM sphingofungin [50]. Because the SAM1-like cluster is the only putative SAM cluster in the two *A. fumigatus* strains examined (Additional file 7) this cluster is most likely the sphingofungin biosynthetic gene cluster, as previously proposed [50].

### Predictions of amino acid substrates for SAM ATs

Serine palmitoyltransferase and the fumonisin AT are members of class II aminotransferases (α-oxoamine synthases). This class of enzymes catalyzes condensation of an amino acid and an acyl compound (e.g., palmitoyl-CoA). Functional analysis of members of this group of aminotransferases indicate that the amino acid corresponding to position 83 in the enzyme aminolevulinic acid synthase (ALAS) affects specificity for the amino acid substrate used in the condensation reaction [51–53]. Results of comparative sequence analysis of orthologs of the fumonisin AT (Fum8) are consistent with these findings. That is, the position in Fum8 orthologs corresponding to ALAS position 83 (e.g., position 580 in the *F. verticillioides* Fum8) is an alanine in FB-producing species, which use alanine as a substrate in fumonisin biosynthesis, and a valine in FC-producing species, which use glycine as a substrate in fumonisin biosynthesis [18]. In the current study, we used this information to predict the amino acid substrates of the SAM1 – SAM5 ATs (Table 3). For example, the SAM1 and SAM2 AT and 8-amino-7-oxononanoate synthase (AONS) exhibit high levels of amino acid identity in the 10-amino acid region that includes the position corresponding to ALAS position 83. In all three ATs, there is a serine at this position (Table 3). Because AONS uses alanine as an amino acid substrate, we predicted that the SAM1 and SAM2 AT enzymes also use alanine as a substrate. If this is indeed the case, then the end of the putative SAM1 and SAM2 metabolites would be more similar to FBs (Fig. 1); i.e., there would be a terminal methyl group next to the amine. Using the same rationale, we made similar predictions for amino acid substrates in biosynthesis of the metabolic products of the SAM3, SAM4 and SAM5 clusters (Table 3). The predicted amino acid substrate for the SAM5 AT is alanine. This is consistent with our proposal that the SAM5 cluster is the AOD biosynthetic gene cluster, because the AOD structure indicates that alanine is the amino acid substrate used in AOD biosynthesis (Fig. 1).

**Table 3:**
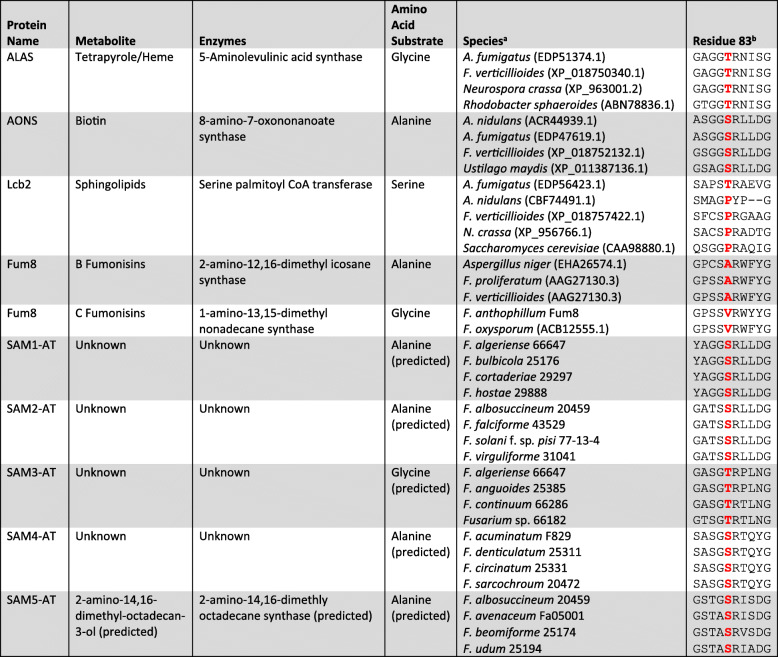
Predicted amino acid sequences of the residue-83 region of selected class II aminotransferases (AT) involved in primary metabolism (ALAS, AONS and Lcb2) and the fumonisin and SAM1 – SAM5 ATs. The amino acid at residue 83 is highlighted with bold red text

^a^ Deduced amino acid sequence data for ALAS, AONS, Lcb2 and Fum8 were obtained from the NCBI GenBank database. GenBank accession numbers for the proteins are indicated in parentheses after the species names

^b^ The abbreviated letters for standard amino acids are used and the residues correspond to amino acids at 83 were highlighted in red (e.g. *A* Alanine, *V* Valine, *S* Serine, *T* Threonine, *P* Proline)

### SAM5 cluster and AOD production

If the SAM5 cluster is the AOD biosynthetic gene cluster, only fusaria with this cluster should be able to produce AOD; whereas fusaria that lack the cluster should not be able to produce AOD. To test this, we examined AOD production by liquid chromatography-mass spectrometry (LC-MS) in 28 *Fusarium* strains, representing 18 species that have the SAM5 cluster and five species that do not have the cluster. The levels of AOD detected in culture extracts of strains that had the cluster varied markedly; extracts from approximately half of the strains had levels of AOD ranging from 3.6 to 30.7 ng/mL, whereas extracts from the other half had levels of AOD that were below the level of detection (< 0.05 ng/mL) to 0.7 ng/mL (Table 4). AOD was detected in culture extracts of two strains of *F. avenaceum*, the species from which AOD was first described [8]. In addition, AOD was not detected in culture extracts from the five species that lack the SAM5 cluster (Table 4).

**Table 4:** Production of 2-Amino-14,16-dimethyl-octadecan-3-ol (AOD) in *Fusarium* strains that have or do not have the SAM5 cluster

Species Complex	Strain No.	Species	SAM5 cluster	AOD (ng/mL)
Babinda	NRRL 25533	*F. babinda*	YES	0.0
	NRRL 25539	*F. babinda*	YES	23.7
Burgessii	NRRL 66647 (KOD 1247)	*F. algeriense*	YES	3.9
	NRRL 66648 (KOD 1248)	*F. algeriense*	YES	11.2
	NRRL 25174	*F. beomiforme*	YES	0.3
Concolor	NRRL 13459	*F. concolor*	YES	4.8
Decemcellulare	NRRL 13412	*F. decemcellulare*	YES	0.0
Fujikuroi - African	NRRL 25311	*F. denticulatum*	YES	3.7
	NRRL 36939	*F. pseudocircinatum*	YES	0.7
	NRRL 25194	*F. udum*	YES	0.2
Fujikuroi - American	NRRL 53147	*F. mexicanum*	YES	0.5
	NRRL 25623	*F. sterilihyphosum*	YES	0.0
	NRRL 47473	*F. mexicanum*	YES	0.2
	NRRL 53293	*Fuarium* sp.	NO	0.0
Fujikuroi - Asian	NRRL 25181	*F. concentricum*	NO	0.0
	NRRL 43689	*F. fractiflexum*	NO	0.0
Fujikuroi-basal	NRRL 52700	*Fuarium* sp.	YES	11.6
Heterosporum	NRRL 20692	*F. graminum*	YES	0.1
	NRRL 20693	*F. heterosporum*	YES	0.0
Nisikadoi	NRRL 45417	*F. gaditjirrii*	YES	10.6
Newnesense	NRRL 66241	*F. newnesense*	YES	0.6
	NRRL 25184	*F. newnesense*	YES	5.6
Sambucinum	NRRL 25348	*F. kyushuense*	NO	0.0
Solani	NRRL 31041	*F. virguliforme*	NO	0.0
Tricinctum	NRRL 62622	*F. acuminatum*	YES	10.2
	NRRL 13321	*F. avenaceum*	YES	9.5
	NRRL 54939	*F. avenaceum*	YES	30.7
	NRRL 25481	*F. trincinctum*	YES	27.3

**Fig. 6:**
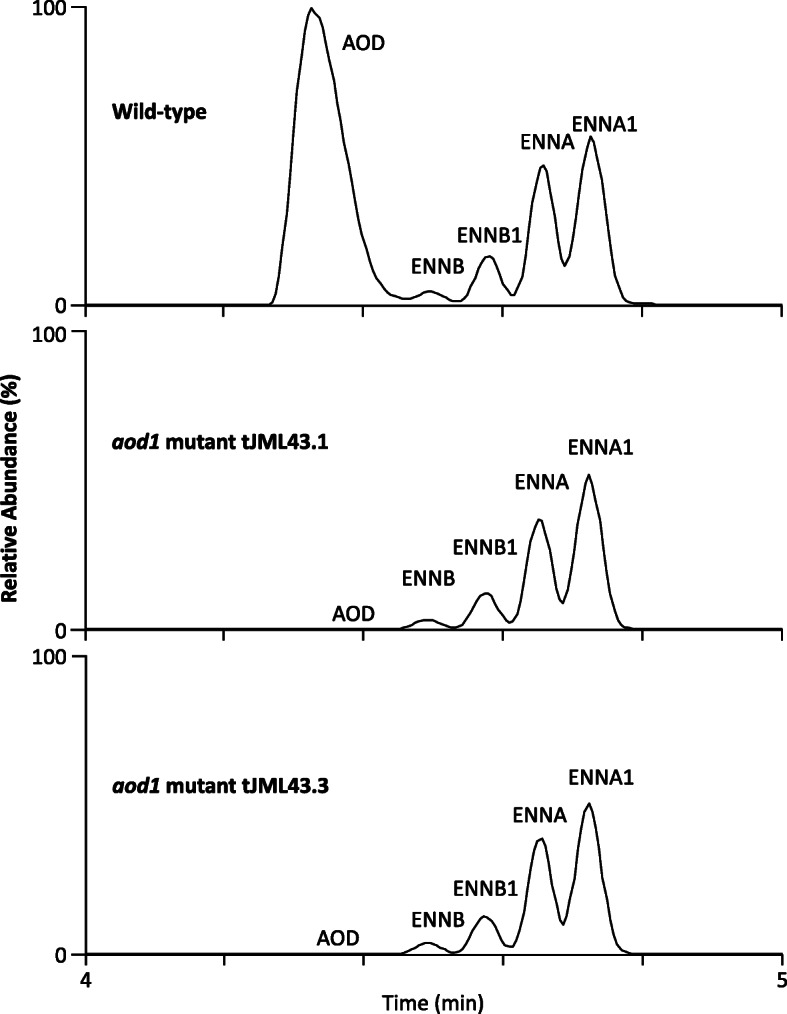
Liquid chromatography-mass spectrometry analysis of AOD and enniatin production in the wild-type (NRRL 25481) and *aod1* mutant strains (tJML43.1 and tJML43.3) of *F. babinda*. The labels ENNA, ENNA1, ENNB and ENNB indicate peaks corresponding to four enniatin analogs

To further test the hypothesis that the SAM5 cluster is the AOD biosynthetic gene cluster, we deleted the SAM5 PKS gene in strains of two distantly related species of *Fusarium* (i.e., *F. babinda* and *F. tricinctum*) and then assessed the ability of the resulting deletion mutants to produce AOD (Additional file 8). LC-MS analysis of two independent mutants of each species revealed that deletion of the SAM5 PKS gene (hereafter *AOD1*) resulted in loss of AOD production (Fig. 6; Additional file 8). In contrast, *AOD1* deletion did not affect the ability of either species to produce four analogs of enniatins, a family of *Fusarium* mycotoxins that are synthesized via a nonribosomal peptide synthetase. We contend that loss of AOD production combined with retention of enniatin production in two independent mutants of two distantly related *Fusarium* species is strong evidence that the *AOD1* gene is required for AOD biosynthesis. Therefore, we conclude that the SAM5 cluster is the AOD biosynthetic gene cluster.

## Discussion

Disruption of sphingolipid metabolism by SAMs is detrimental in the case of fumonisin mycotoxins but could also be beneficial if medical and other applications for SAMs can be developed [36–38]. Fungi are a potential source of pharmaceutical SAMs, and SAM production has been reported in species of the Dothideomycetes, Eurotiomycetes and Sordariomycetes, three fungal classes that include numerous species that are important to agriculture, medicine and fermentation industries. Except for fumonisins, however, there is a paucity of information on SAM biosynthesis in fungi. In this study, therefore, we developed a method to identify fungal SAM biosynthetic genes. The method was based on the current understanding of fumonisin biosynthesis and its similarities to sphinganine biosynthesis, as well as the tendency for fungal secondary metabolite biosynthetic genes to be clustered. Using the method, we identified five putative SAM biosynthetic gene clusters in *Fusarium*. We also identified putative SAM clusters in species in 24 other fungal genera from the Dothideomycetes, Eurotiomycetes, Lecanoromycetes and Sordariomycetes.

### Potential metabolic products of SAM clusters

Deletion analysis of the SAM5 PKS gene *AOD1* provided evidence that the SAM5 cluster is the AOD biosynthetic gene cluster. Although our analyses did not demonstrate that the metabolic products of the SAM1 – SAM4 clusters are SAMs, given the precedence of fumonisin biosynthesis and the results of the *AOD1* deletion analysis, we conclude that SAMs are the most likely products of the four clusters. Nevertheless, future chemical and gene-function analyses are required to demonstrate that the clusters are involved in SAM biosynthesis. The SAM1-like cluster in *Aspergillus* species is perhaps the best candidate for such an analysis because of evidence linking the cluster to sphingofungin. This analysis would be facilitated by analytical methods that have already been developed for sphingofungin [12].

Two lines of evidence connect the SAM1-like cluster in *A. fumigatus* to sphingofungin production: 1) the SAM1-like cluster is the only SAM cluster detected in *A. fumigatus*, a species that has been previously reported to produce sphingofungins [12]; and 2) the gene content of the SAM1-like cluster is largely consistent with the structure of sphingofungins (Fig. 1). From the structure of sphingofungin C and and the predicted functions of the genes in the SAM1-like cluster in *A. fumigatus*, we have proposed a sphingofungin C biosynthetic pathway (Additional file 9). Functional analyses of genes in the putative sphingofungin cluster are required to demonstrate that the cluster is indeed responsible for sphingofungin formation and to assess the accuracy of the proposed pathway.

From our results, we cannot draw firm conclusions as to the structures of the metabolic products of the SAM1 – SAM4 clusters. Nevertheless, as noted above, the products are likely to be SAMs. Furthermore, the unique amino acid sequences of the SAM1 – SAM4 PKSs suggest that each of the four PKSs could have unique activities that lead to formation of four polyketides that differ in carbon-chain length and/or in the presence and position of methyl groups along the carbon chain. Differences in gene content of the clusters provides additional clues about possible structures. For example, the relatively few enzyme-encoding genes in the SAM1 – SAM4 clusters suggest that the metabolic products have relatively simple structures compared to fumonisins. The fumonisin cluster includes 10 enzyme-encoding genes in addition to the PKS, AT and SDR genes, although four of the genes are not required for formation of the wild-type complement of *F. verticillioides* fumonisins. By contrast, the SAM1 – SAM4 clusters have 0–3 enzyme-encoding genes in addition to the PKS, AT and SDR genes. The SAM1 cluster has one dioxygenase and one monooxygenase gene in addition to the PKS, AT and SDR genes. Because these two classes of enzymes typically catalyze oxygenation reactions [54, 55], it is possible that the metabolic product(s) of the SAM1 cluster includes oxygen atoms attached to positions along the carbon chain of a sphinganine-like molecule.

The amino acid residue at position 83 of SAM ATs can also provide clues about structures of the metabolic products of the SAM clusters. Based on our current understanding, we predicted that alanine is the preferred substrate for the SAM1, SAM2, SAM4 and SAM5 ATs, whereas glycine is the preferred substrate for the SAM3 AT (Table 3). Thus, even though the SAM3 and SAM5 clusters do not include any known enzyme-encoding genes other than PKS, AT and SDR genes, we propose that metabolic products of the two clusters could be structurally distinct because of the potential for differences in activities of the SAM3 and SAM5 PKSs and in the amino acid substrate specificities of the SAM3 and SAM5 ATs.

Despite the absence of an SDR gene in eight homologs of the *Fusarium* SAM3 cluster and almost half of the SAM clusters in other fungi (Additional files 3 and 7), we considered these clusters to be SAM clusters because of their relationships to SAM clusters with SDR genes, and because of evidence indicating a lack of specificity of SDRs involved in sphinganine and fumonisin biosynthesis. Although deletion of the *F. verticillioides* fumonisin SDR gene (*FUM13*) markedly reduced formation of 3-hydroxyl fumonisins, formation was not completely blocked; 10% of the fumonisin analogs produced by *fum13* deletion mutants had a 3-hydroxyl, while 90% had a 3-keto group [56]. In addition, the *F. verticillioides FUM13* homolog could partially complement a strain of *Saccharomyces cerevisiae* in which the 3-ketosphinganine reductase gene was inactivated [57]. Thus, although some of the putative SAM clusters identified in the current study lack a functional SDR gene, it is possible that these clusters yield a SAM product if another SDR can compensate for the absence of an SDR gene in the clusters.

### Potential ecological significance of SAM clusters

Because of the essential roles of sphingolipids in membrane structure and signal transduction in eukaryotic cells, SAM production is likely a mechanism that organisms use to manipulate their hosts, competitors, pathogens and/or predators. AAL toxins exemplify this idea. Production of this family of SAMs by the fungus *A. arborescens* contributes to virulence of the fungus on tomato genotypes that lack the AAL toxin resistance gene [1]. Although multiple studies indicate that fumonisin production does not affect the ability of *F. verticillioides* to cause maize ear rot, production can enhance the ability of the fungus to cause maize seedling blight under some environmental conditions [22, 58–60]. In addition, there is evidence that fumonisin production contributes to the ability of *F. verticillioides* strains to compete with one another in maize seedlings [61]. Thus, the metabolic products of the novel SAM clusters described in this study could provide an ecological advantage to fungi by enhancing their ability to compete with other microorganisms or cause plant disease. The widespread occurrence of SAM clusters in phylogenetically diverse species of *Fusarium* suggests that the ability to inhibit sphingolipid metabolism in competitors and/or hosts via SAM production is also widespread. However, the widespread absence of SAM clusters among fusaria indicates that the ability to inhibit sphingolipid metabolism is not an essential trait. The same situation likely applies to other fungal genera as well given the low frequency of occurrence of SAM clusters in fungi represented in the NCBI database.

The presence of two or three SAM clusters per genome of some *Fusarium* species suggests that the species can produce two or three structurally distinct SAMs. Although the ecological significance of production of multiple SAMs by one species is not known, there are several possibilities. For example, production of multiple SAMs could provide redundancy of function. That is, because different SAM structures can affect different sphingolipid biosynthetic enzymes [3], production of structurally distinct SAMs could allow a species to inhibit multiple enzymes, which could in turn result in more effective or more fine-tuned manipulation of sphingolipid biosynthesis. Another possibility is that in a species that produces two structurally distinct SAMs, one SAM could enhance competition with one organism, while the other could enhance competition with a different organism(s). Yet another possibility is that different SAMs may function more effectively under different environmental conditions encountered by an organism or during different phases of its lifecycle.

### Evolution of SAM clusters

The phylogenetic relationships of the SAM PKS, AT and SDR genes provide insights in the evolutionary histories of the SAM clusters. For example, while all the PKS genes in the *Fusarium* SAM clusters were members of the previously described Reducing PKS Clade III [41], PKS genes from different SAM clusters were not necessarily more closely related to one another than they were to PKS genes that were not in SAM clusters. This is exemplified by PKS Clade 11, which is comprised of two smaller clades. One of the smaller clades consists exclusively of SAM5 PKS genes, whereas the other smaller clade consists of PKS genes from a non-SAM cluster (Fig. 2). This finding indicates the SAM5 PKS is more closely related to a non-SAM PKS than it is to the other SAM PKSs examined in this study. Likewise, the SAM1 – SAM5 PKS genes along with the non-SAM PKS genes in PKS Clade 42 form a large and well-supported clade that excludes the fumonisin PKS gene (Fig. 2).

A relatively distant relationship of the fumonisin cluster to other *Fusarium* SAM clusters is also consistent with differences in SAM AT genes. As noted above, SAM ATs are members of the enzyme family class II aminotransferases. Enzymes in this family can consist of a single protein (e.g., aminolevulinic acid synthase) or of two protein subunits (e.g., the Lcb1 and Lcb2 subunits of serine palmitoyltransferase) [62, 63]. The sizes and sequences of the SAM1 – SAM5 ATs indicate that they are members of the single-protein type of class II aminotransferases. In contrast, the sequence and size of the fumonisin AT (Fum8) suggest that it is a fusion of Lcb1- and Lcb2-like subunits [26, 64]. Thus, characteristics of the SAM PKS and AT genes suggest that the fumonisin biosynthetic gene clusters has an evolutionary history that is distinct from the other SAM clusters.

Results of phylogenetic analyses reported in the current study are consistent with the SAM1 – SAM5 clusters diverging from a common ancestral SAM cluster. However, analyses of more extensive datasets of PKSs and ATs from diverse fungi could provide further insights in support of or against this hypothesis. PKS and AT genes from each *Fusarium* SAM cluster are more closely related to genes in SAM clusters from other fungal genera than they are to genes from the other *Fusarium* SAM clusters (Additional file 1). Further, *Fusarium* homologs are among the more recently diverging lineages in trees inferred from SAM PKSs, ATs and SDRs. These findings suggest that if there is a common ancestral SAM cluster of the *Fusarium* SAM1 – SAM5 clusters, the ancestral cluster did not assemble in *Fusarium*, and that the individual SAM clusters (SAM1 – SAM5) diverged from one another before they were introduced into *Fusarium*.

The discontinuous distribution of the SAM clusters in *Fusarium* was such that for most species with a SAM cluster, there were at least as many closely related species that lacked the cluster as there were that had the cluster (Fig. 5). The SAM2 cluster was the exception; it exhibited a nearly continuous distribution in the Solani and Decemcellulare complexes, the only two species complexes in which it was detected. The almost continuous distribution of the SAM2 cluster suggests an almost continuous selection for the SAM2 cluster during divergence of members of the Solani and Decemcellulare complexes. In contrast, the discontinuous distribution of the other SAM clusters and the evidence for multiple losses of SAM clusters in *Fusarium* (Fig. 5) suggest intermittent selection of the other clusters.

Results of NOTUNG, SH-AU and divergence analyses in this study provided support for 15–30 HGT events of SAM clusters among *Fusarium* species (Table 1). Such events have likely contributed to the discontinuous distribution of the clusters in *Fusarium*. The presence of homologous SAM clusters in the Dothideomycetes, Eurotiomycetes, Lecanoromycetes and Sordariomycetes indicate that HGT of SAM clusters could have occurred between fungal genera and even between classes of fungi. Together, the inferences of HGT of SAM clusters among fusaria reported in the current study and inferences of HGT of PKS and nonribosomal peptide synthetase genes between members of the *F. incarnatum*-*equiseti* species complex and other lineages of *Fusarium* [49] provide evidence that HGT has significantly impacted the distribution of secondary metabolite biosynthetic genes and, therefore, production of secondary metabolites in *Fusarium*.

Results of synonymous site divergence analysis contributed to inferences of HGT by revealing the presence of relatively closely related SAM gene homologs in relatively distantly related species (Tables 1 and 2; Additional file 6). Such results were typically consistent with conflicts between the SAM gene trees and the species tree (Additional file 5). In some cases, however, conflicts between SAM gene trees and the species tree were associated with unexpectedly high estimates of synonymous divergence among homologs of SAM genes. Two examples of this were the divergence estimates for SAM4 genes in *F. phyllophilum* and *F. udum*, which are members of the African clade of the Fujikuroi complex, and the divergence estimates for fumonisin genes in *F. anthophilum* and *F. bulbicola*, which are members of the American clade of the Fujikuroi complex (Fig. 5, Additional files 5 and 6). The presence of relatively distantly related genes in closely related species has been attributed to multiple causes; e.g., interspecies hybridization and incomplete lineage sorting of ancestral alleles [18, 65]. Here, we propose a scenario in which the presence of distantly related SAM genes in closely related *Fusarium* species could result from loss and HGT. In the scenario, a SAM cluster present in an ancestral *Fusarium* species was differentially lost during species divergence such that some descendant species had the cluster and others did not. Among the descendant species that lacked the cluster, one re-acquired the cluster from a distantly related species via HGT. As a result, some closely related descendent species have distantly related homologs of the same SAM cluster: one homolog inherited vertically from the common ancestral species, and the other homolog acquired by HGT from a distantly related species. A variation of this scenario is that two closely related species lacking a SAM cluster acquired it via HGT from distantly related donors, again resulting in the presence of distantly related cluster homologs in closely related species. Both scenarios are consistent with the discontinuous distribution of SAM clusters. The latter scenario was one of two alternative hypotheses used to explain distantly related homologs of the fumonisin clusters in closely related members of the Fujikuroi complex [18].

### Are there alternative SAM biosynthetic pathways?

The structures of fungal SAMs that have been described indicate that they are derived from linear 16- or 18-carbon-long acyl molecules (Fig. 1). To our knowledge, the only fungal enzymes that catalyze synthesis of such molecules are fatty acid synthases (FASes) and PKSs. Our approach to identify SAM clusters assumed that PKSs are responsible for synthesis of the linear acyl precursors of SAMs. However, we cannot exclude the possibility that biosynthesis of some other SAMs could employ a FAS rather than a PKS. The requirement of an FAS for formation of aflatoxins, for example, demonstrates that FASes can participate in biosynthesis of secondary metabolites [66]. Fungal FASes are composed of α and β subunits that are encoded by different genes. Aflatoxin-producing species of *Aspergillus* have two sets of FAS genes: 1) the *fasA* and *fasB* genes required for synthesis of fatty acids used in primary metabolism; and 2) the *aflA* and *aflB* genes required for synthesis of a fully reduced, six-carbon chain used to initiate aflatoxin biosynthesis [67, 68]. The requirement of an FAS for synthesis of aflatoxins indicates that FASes could be involved in synthesis of other secondary metabolites as well. Identification of fungal gene clusters that include FAS gene homologs as well as AT and SDR genes would provide evidence for alternative SAM biosynthetic pathways that use FASes rather than PKSes. *Aspergillus fumigatus* has only one set of FAS genes, which are closely related to the primary metabolic FAS genes in other *Aspergillus* species [50]. This indicates that the long-chain backbone of sphingofungin is most likely not derived from a FAS, but more likely from the SAM1-like PKS noted above. In contrast, *F. avenaceum* has two sets of FAS genes [69]. The first set is closely related and the second set is distantly related to the primary metabolic FAS genes in other *Fusarium* species. There is also an amino transferase gene located near the second set of *F. avenaceum* FAS genes. However, the region of the *F. avenaceum* genome with the second set of FAS genes and the aminotransferase gene is highly similar to genes in the apicidin biosynthetic gene cluster [69]. Apicidin is not a SAM, but instead is a nonribosomal peptide with an eight-carbon-long linear carbon chain attached to it. Therefore, the second set of FAS genes in *F. avenaceum* are more likely to be part of the apicidin biosynthetic gene cluster than an AOD cluster [32]. This conclusion is consistent with previous reports of apicidin production in *F. avenaceum* [69].

## Conclusion

Decades of chemical analyses have identified numerous fungal secondary metabolites that have pharmaceutical and agricultural applications. More recent analyses of hundreds of fungal genome sequences indicate that fungi have the genetic potential to produce far more secondary metabolites than indicated by earlier chemical analyses. This points to the tremendous potential contributions of fungal secondary metabolites to medicine, agriculture and other human endeavors. As a result, there are significant efforts to exploit fungal genome sequence data in order to identify novel secondary metabolites [70, 71]. Predictions of structural features of potential metabolic products of biosynthetic gene clusters can aid in the elucidation of chemical structures via MS and nuclear magnetic resonance-based methods [72]. Thus, identification of putative SAM clusters and predictions of structural features of the corresponding SAMs has potential to contribute to identification of novel secondary metabolites.

The status of fumonisins as one of the mycotoxin families of most concern to food and feed safety necessitates additional control measures to reduce the presence of these mycotoxins in food and feed crops. Understanding the role of production of trichothecene mycotoxins in the ecology of *Fusarium* species has provided important insights into methods to control trichothecene contamination in wheat and barley [73, 74]. The role(s) that fumonisins play in the ecology of fusaria is not understood as well as the role of trichothecene production. We posit that improved understanding of the chemical diversity of SAMs produced by fungi as well as the roles that SAMs play in fungal ecology could provide insights into approaches to control fumonisin contamination. The results of the current study have potential to contribute to such efforts. Furthermore, some fusaria that have SAM clusters other than or in addition to the fumonisin cluster are significant plant pathogens. For example, *F. circinatum* has the SAM4 cluster and causes pitch canker of pine; *F. udum* has the SAM4 and SAM5 clusters and causes Fusarium wilt of chick pea; and *F. virgiliforme* has the SAM2 cluster and causes soybean sudden death syndrome (Fig. 5) [75]. Identifying the metabolic products of the SAM clusters and determining the role of SAM production in the ecology of these fungi could provide insight into methods to control the crop diseases caused by the fungi.

The presence of PKS, nonribosomal peptide synthetase (NRPS), and/or terpene synthase genes in biosynthetic gene clusters provides an indication of whether the metabolic products of the clusters are derived from a polyketide, nonribosomal peptide and/or terpene. Sequence-based assessments of PKS domain content or NRPS modular content can provide additional information as to the structures of the metabolic products of the clusters. For example, PKSs with ketoreductase, dehydratase and enoyl reductase domains tend to synthesize more linear polyketides, whereas PKSs that lack these domains typically synthesize aromatic polyketides [39, 41, 42]. The presence of other classes of genes in a cluster can provide additional clues about structural features of the metabolic products of clusters [76]. In the current study, we used these ideas to develop a method to identify fungal gene clusters that are likely to be responsible for synthesis of inhibitors of sphingolipid metabolism. It is possible that improved understanding of the genetics and biochemistry of other classes of metabolic inhibitors will yield similar methods to identify novel biosynthetic gene clusters and in turn novel analogs of the inhibitors. Thus, the approach used in this study has potential to expand the repertoire of metabolic inhibitors available for medical and other applications.

## Methods

### Fungal strains and genome sequences

The 343 *Fusarium* strains examined in this study are listed in Additional file 10. Genome sequences of 30 strains have been reported previously, and were obtained from the Joint Genome Institute, Munich Information Center for Protein Sequences (MIPS), or National Center for Biotechnology Information (NCBI). Genome sequences for 10 strains were generated using a HiSeq Illumina sequencing platform at the Beijing Genome Institute (Hong Kong). The remaining genome sequence data were generated in-house at the USDA ARS NCAUR using MiSeq Illumina (Illumina, Inc.) (292 genomes) and Ion Torrent PGM™ (Thermo Fisher Scientific Inc.) (11 genomes) sequencing platforms. To prepare genomic DNA for sequencing, fungal mycelia were grown in liquid GYP medium (2% glucose, 1% peptone, and 0.3% yeast extract) for 2–3 days, harvested by filtration, lyophilized, and ground to a powder. Genomic DNA was then extracted using the ZR Fungal/Bacterial DNA MiniPrep kit (Zymo Research, Irvine, CA), the Qiagen Genomic-Tip 20/G protocol, or a previously described chloroform-phenol-based method [77] To prepare DNA sequencing libraries, we used the Nextera XT DNA library Preparation Kit for the MiSeq platform and the NEBNext Fast DNA Fragmentation & Library Prep Set for the Ion Torrent PGM™ platform. Sequence reads were imported into CLC Genomics Workbench version 8.0–12.0 (CLC bio-Qiagen, Aarhus, Denmark), and then screened against genome sequences of 84 bacterial species in order to remove contaminating DNA introduced by reagents. The reads were then trimmed to remove low-quality data and then assembled using the following parameter settings in CLC Genomics Workbench: word size = 20; bubble size = 50; minimum contig length = 500; auto-detect paired distances = checked; and perform scaffolding = checked). Whole genome sequence data generated during this study have deposited at DDBJ/ENA/GenBank under accessions: JAADJF000000000, JAADJG000000000, JAADYS000000000, JAAFOW000000000, JAAGWO000000000, JAAGWP000000000, JAAGWQ000000000, JAALXH000000000, JAALXI000000000, JAALXJ000000000, JAALXK000000000, JAALXL000000000, JAALXM000000000, JAALXN000000000, JAAMOD000000000, JAANQP000000000, JAAOAG000000000, JAAOAH000000000, JAAOAI000000000, JAAOAJ000000000, JAAOAK000000000, JAAOAL000000000, JAAOAM000000000, JAAOAN000000000, JAAOAO000000000, JAAOAP000000000, JAAOAQ000000000, JAAOAR000000000, JAAOAS000000000, JAAOAT000000000, JAAOAU000000000, JAAOAV000000000, JAAOAW000000000, JAAOAX000000000, JAAOAY000000000, JAAQPE000000000, JAAQPF000000000, JAAQPG000000000, JAAQRH000000000, JAAQRI000000000, JAAQRM000000000, JABCJS000000000, JABCJT000000000, JABCJU000000000, JABCJV000000000, JABCJW000000000, JABCJX000000000, JABCJY000000000, JABCJZ000000000, JABCKB000000000, JABCKC000000000, JABCKD000000000, JABCKE000000000, JABCQV000000000, JABEEJ000000000, JABEEK000000000, JABEEL000000000, JABEEM000000000, JABEEN000000000, JABEEO000000000, JABEEP000000000, JABEEU000000000, JABELF000000000, JABEVY000000000, JABEXW000000000, JABEYC000000000, JABFAI000000000, JABFAK000000000, JABSTN000000000.

### Gene prediction and functional annotation

Gene prediction for 303 *Fusarium* genomes was performed with the program AUGUSTUS trained for *F. graminearum* using ab initio gene prediction method [78, 79]. The gene models were functionally annotated using the Blast2GO software incorporated with Basic Local Alignment Search Tool (BLAST) analysis [80] against the NCBI-NR protein database [81, 82] and/or manual BLAST analysis and annotation by aligning genomic DNA sequences using the program MEGA 7.0 [83]. Each predicted gene in a genome was assigned a locus tag designation, which consisted a five-letter (or digit) prefix indicative of the species name or strain designation, an underscore, and the 5-digit number generated by AUGUSTUS (Additional files 3 and 10).

### Identification of putative SAM gene clusters

Two parallel approaches were used to identify PKS, AT and SDR genes potentially involved in SAM biosynthesis. First, *F. verticillioides* homologs of the fumonisin PKS (*FUM1*), AT (*FUM8*) and SDR (*FUM13*) genes were used as query sequences in BLASTn and BLASTx analysis of predicted mRNA and amino acid sequences in the genome sequences of the 343 *Fusarium* strains. Because Augustus-predicted genes that are located the same contig are numbered sequentially, we used the BLAST results and custom Perl scripts to parse PKS, AT and SDR genes that were located within five genes of one another along the same contig. In the second approach, all predicted protein sequences from the *Fusarium* genome sequences were subjected to OrthoFinder analysis [84] in batches of 10–15 genome sequences. Predicted proteins from *F. fujikuroi*, which also has fumonisin biosynthetic genes, were included in each batch as a reference genome. Sequences that were closely related to the deduced amino acid sequence of the fumonisin PKS, AT and SDR were parsed from the OrthoFinder output data, and subjected to HMMPfam analysis [85, 86] to confirm the identity of the parsed sequences as PKSs, ATs or SDRs, respectively. Genome sequences with one or more PKS, AT and SDR genes identified by BLAST and or OrthoFinder analysis were subjected to antiSMASH analysis (version 3.0 and 4.0) to determine whether any of the PKS, AT and SDR genes from the same genome sequence were located in the same antiSMASH-predicted gene cluster [87–89]. The genome sequences data were also subjected to Blast2GO analysis [82] to help predict functions of cluster genes. We also confirmed functions of some genes by manual BLASTx/BLASTp analysis against the non-redundant (NR) protein sequence database at NCBI [90].

The PKS, AT and SDR gene sequences that occurred in antiSMASH-predicted gene clusters were aligned using MUSCLE as implemented in MEGA 7.0 [83] and/or using MAFFT [91]. The resulting alignments were subjected to maximum likelihood tree-building analysis using IQ-Tree with ultrafast bootstrapping [92] to assess the relationships of members of each of the three gene families. The order and orientation of genes in the antiSMASH-predicted clusters were also examined manually using the program Sequencher (Gene Code Corp.). The IQ-Tree-inferred PKS, AT and SDR trees as well as comparisons of the predicted gene clusters were then used to identify homologous gene clusters. PKS, AT and SDR gene sequences from orthologous clusters were then analyzed separately using the maximum likelihood tree-building analysis in IQ-Tree [92].

To identify putative SAM biosynthetic gene clusters in fungi other than *Fusarium*, we used PKS, AT and SDR gene sequences from selected homologs of each putative *Fusarium* SAM cluster as query sequences in BLASTx analysis against the NR fungal protein sequence database at NCBI. For species in the NCBI database that yielded hits with a high BLAST score (E-value < 1 × 10^− 50^) for the PKS and AT genes, we determined whether the genes were located near one another using Locus Tag designations as well as manual examination of the positions of the genes on contigs. Strains/species with PKS, AT and, in some cases, SDR genes located within five genes of one another on a contig are indicated in Additional file 7. Predicted protein sequences of these non-*Fusarium* PKS, AT and SDR sequences were aligned with the *Fusarium* sequences noted above using MAFFT, and the resulting alignments were subjected to maximum likelihood tree-building analysis using IQ-Tree.

### Species phylogeny

Species phylogenies were inferred from coding region sequences of 13 housekeeping genes (Additional file 11) mined from a local database of *Fusarium* genome sequences described above using the BLASTn and/or BLASTx functions in CLC Genomics Workbench. Coding region sequences that differed substantially from reference sequences of *F. avenaceum*, *F. fujikuroi*, *F. graminearum*, *F. oxysporum*, and *F. solani* f. sp. *pisi* were examined and manually annotated as necessary. Orthologs of each housekeeping gene were first aligned using MAFFT [91], and then the resulting alignments were concatenated using SequenceMatrix [93]. The concatenated alignment was then subjected to maximum likelihood analysis with 1000 bootstrap replicates using IQ-Tree 1.6.8 [92].

A preliminary species tree was inferred from the entire set of 343 *Fusarium* strains along with three *Cylindrocarpon* strains, which served as an outgroup. This tree included multiple strains of some *Fusarium* species as well as multiple strains for which the species identity had not been determined. The topology of the preliminary tree suggested that some strains for which the species identity was not determined were the same species as other known species included in the analysis, while other strains appeared to be phylogenetically-distinct novel species. We then used sequence comparisons of the elongation factor 1-α gene (*TEF1*) as an initial and rapid method to assess whether unidentified strains were likely to be novel species. *TEF1* as well as *RPB1* and *RPB2*, which encode subunits of RNA polymerase, are routinely used to determine species identities of *Fusarium* isolates [46, 94]. In this analysis, we considered two strains to be the same species if their *TEF1* nucleotide sequences (including introns) were > 99.5% identical, but potentially different species if their *TEF1* sequences were < 99.5% identical. Strains for which the sequence identity was < 99.5% to any other strain/species included in the study were then subjected to a more rigorous phylogenetic analysis using *TEF1*, *RPB1* and *RPB2*. Using this approach we estimated that our data of 343 genome sequences corresponded to 186 species. However, more detailed analyses are required to confirm that some strains are indeed novel species.

### Assessments of potential horizontal gene transfer events

Three methods were used to assess potential horizontal gene transfer (HGT) events of putative SAM clusters as previously described [18, 49]. The first method was reconciliation analysis using the program NOTUNG version 2.9 [95, 96]. The species tree used in NOTUNG analysis was inferred by maximum likelihood analysis of concatenated coding regions of 13 HK genes (Additional file 11) from 96 fusaria that had one or more putative SAM clusters. Gene trees in the NOTUNG analysis were inferred by maximum likelihood analysis of coding region sequences of the PKS, AT and SDR genes from SAM clusters; each SAM cluster was analyzed separately. For SAM3, only PKS and AT genes were used because not all SAM3 cluster homologs had an SDR gene. If there were no branch conflicts among the PKS, AT and SDR trees for a given SAM cluster, gene trees were inferred from concatenated alignments of the three genes. If there were branch conflicts in the trees, we analyzed alignments of both individual and concatenated gene sequences. Each NOTUNG-inferred HGT event was assessed by: 1) constraint analysis using Shimodaira-Hasegawa and Approximately Unbiased tests [97, 98]; and 2) estimates of the number of synonymous differences per synonymous site (*d*_*S*_ values) in the PKS, AT and SDR genes from a given cluster versus 13 housekeeping genes [65, 99].

In general, *d*_*S*_ values were considered to be consistent with horizontal transfer when *d*_*S*_ values for SAM genes were less than *d*_*S*_ values for housekeeping genes (ratio of *d*_*S*_ SAM genes to *d*_*S*_ housekeeping genes, *d*_*S*_ ratio, < 1.0). However, we also considered that a *d*_*S*_ ratio of 1.0–1.5 could be consistent with ancient horizontal transfer between distantly related species (i.e., species that were not members of the same species complex or sister species complexes). Our rational for considering this less stringent *d*_*S*_ ratio is based on the observation that secondary metabolite genes tend to diverge more rapidly than housekeeping genes [49]. A low *d*_*S*_ ratio would be expected for a recent transfer event, but over time, possibly through multiple speciation events, as the transferred genes diverge from their ancestral genes the *d*_*S*_ ratio would be expected to increase. Given sufficient time, the *d*_*S*_ ratio could exceed 1.0, but still be less than a ratio resulting from vertical inheritance, because the length of time of gene divergence would still be less for horizontal transfer than for vertical inheritance.

### Analysis of AOD and enniatin production

Analysis of AOD and enniatin production was adapted from a previously described method [8]. Strains of *Fusarium* were grown on V-8 juice agar [100] for 1 week, and then a ~ 3-mm^2^ piece of the resulting culture was excised and transferred to cracked maize kernel medium (2.5 g cracked maize kernels and 2.0 mL distilled water combined in a 4-dram glass vial and autoclaved for 20 min at 120 °C). Inoculated vials were loosely capped to allow for air exchange, and then incubated in the dark at room temperature for 10 days. The resulting cultures were extracted in the vials with 6 ml of methanol for 3 h with shaking at 100 rpm followed by filtration through Whatman grade 2 V qualitative filter papers (GE Healthcare, Chicago, IL, USA). Detection and quantification of AOD was done with a liquid chromatography-mass spectrometry (LC-MS) system, consisting of a Thermo Dionex Ultimate 3000 chromatography system coupled to a Thermo QExactive high resolution tandem mass spectrometer (ThermoScientific). The LC system employed a 50 mm × 2 mm Luna C18 column (Phenomenex) and a solvent system consisting of 40 to 95% aqueous methanol gradient over 5 min with a flow rate of 0.6 mL/min. The MS system was equipped with an electrospray ionization interface operated in positive ionization mode. Ten-μL aliquots of the methanol extract were injected into the LC. Observation of the [M + H] + ion (m/z 314) was used in the detection of AOD in comparison to an analytical standard. Control of the LC-MS system and evaluation of acquired data was done with the Thermo Xcalibur LC-MS software. Detection and quantification of AOD and enniatins was based on comparisons of retention times, masses and mass spectra to standards.

### Deletion of SAM5 PKS gene

We used a minor modification of a previously described method [101] to generate deletion mutants of the SAM5 cluster PKS gene (hereafter *AOD1*) in *F. babinda* and *F. tricinctum*. Briefly, 1.5 kb of DNA flanking the 5′ end (upstream fragment) and 1.4–1.5 kb of DNA flanking the 3′ end (downstream fragment) of the *AOD1* coding region were PCR amplified from genomic DNA prepared from a wild-type strain of each species; i.e., *F. babinda* strain NRRL 25539 and *F. tricinctum* strain NRRL 25481. Fusion PCR was then used to fuse the upstream and downstream fragments to a PCR-amplified hygromycin B resistance gene (*hygB*, 1.5 kb) to form the *AOD1* deletion cassette for each species as shown in Additional file 8. *hygB* was amplified from plasmid pJML31.1, which was constructed by amplifying *hygB* (GenBank Accession no. HM623915) from plasmid pA-Hyg-OSCAR [102] and cloning the resulting amplicon into the commercial vector pCR-XL-TOPO (Invitrogen). The amplified deletion cassettes were cloned into pCR-XL-TOPO to yield plasmid pJML43.1 with a 4.0 kb *F. babinda AOD1* deletion construct and plasmid pJML42.1 with a 3.9 kb *F. tricinctum AOD1* deletion construct. The presence of the deletion constructs in these plasmids was confirmed by digestion with restriction enzyme *Eco*RI. Each deletion cassette was PCR amplified from the plasmid for use in protoplast-mediated transformation with the wild-type strain of the corresponding species. Protocols for generating protoplasts and transformation were essentially as previously described [103]. Identification of *AOD1* deletion mutants (*aod1* mutants) from among hygromycin-resistant transformants was done by diagnostic PCR that assessed the presence of the deletion construct and absence of *AOD1* in transformants (Additional file 8). Based on the diagnostic PCR, we selected two *F. babinda* (strains tJML43.1 and tJML43.3) and two *F. tricinctum* (strains tJML42.21 and tJML42.23) *aod1* mutants for analysis of AOD production using the cracked maize kernel culture conditions and LC-MS method described above. All PCR primers used to generate the mutants or in diagnostic PCR are shown in Additional file 8.

## Supplementary information

**Additional file 1. **Phylogenies inferred by maximum likelihood analysis of alignment of predicted amino acid sequences of SAM polyketide synthase (PKS, **Additional file 1 Figure 1**), aminotransferase (AT, **Additional file 1 Figure 2**) and short-chain dehydrogenase reductase (SDR, **Additional file 1 Figure 3**) genes from *Fusarium* species and other ascomycetous fungi. The six phylogenetically distinct clades are color-coded according to genes in SAM cluster and labeled with known fumonisin and novel SAM1-SAM5 to the right of the tree. The prefix for protein designations for PKS, AT and SDR gene homologs from other ascomycetous fungi corresponding to NCBI GenBank accession are listed in Additional file 6 and the locus tag numbers for *Fusarium* genes are listed in Additional file 3.

**Additional file 2. **Organization of the genes and flanking genes in putative SAM clusters: SAM2 (**Additional file 2 Figure 1**), SAM3 (**Additional file 2 Figure 2**)*,* SAM5 (**Additional file 2 Figure 3**) and *FUM* (**Additional file 2 Figure 4**) from two representative *Fusarium* strains (red-outlined box) in phylogenetic tree inferred by maximum likelihood analysis of nucleotide sequences of PKS gene coding regions with 1000 bootstrap. Colored arrows represent genes and flanking genes of each SAM clusters. The direction of the arrows indicates direction of gene transcriptions. Yellow arrows indicate PKS, SDR and AT genes; and colors indicate genes predicted to have other functions based on sequence homology. The prefixes for locus tag number of gene or flanking gene designations corresponding to each SAM clusters are listed in Additional file 3.

**Additional file 3. **Putative SAM clusters in selected species/strains of *Fusarium*. The first sheet of the Excel file provides summary information for 147 representative SAM cluster homologs out of the 208 clusters identified during this study. The information includes the arrangement, contig information, and orientation of genes in the clusters in 110 strains representing 85 species of *Fusarium*. Our analysis included 2–19 strains of some species (e.g., *F. avenaceum* and *F. proliferatum*). Additional file 3 includes information for a maximum of three representative strains per species. In the file, each SAM cluster is highlighted with a different color: yellow for the fumonisin cluster; magenta for SAM1, orange for SAM2, purple for SAM3, green for SAM4, blue for SAM5. Gene functions were predicted by Blast2GO. The symbol of ψ indicates a pseudogene. Rows highlighted with red indicate errors in AUGUSTUS-predicted gene annotation (e.g., automated prediction indicated that two different genes were one gene). Other sheets in the Excel file summarize information for each SAM cluster separately.

**Additional file 4. **Distribution of SAM clusters in 87 representative *Fusarium* genome sequences.

**Additional file 5. **Phylogenetic tree from NOTUNG reconciliation analysis inferred horizontal transfer events for each SAM cluster, SAM1 (**Additional file 5 Figure 1**), SAM2 (**Additional file 5 Figure 2**), SAM3 (**Additional file 5 Figure 3**), SAM4 (**Additional file 5 Figure 4**), and SAM5 (**Additional file 5 Figure 5**). In the NOTUNG analysis, the species tree was inferred from concatenated alignments of the coding region sequences of 13 housekeeping genes from 96 representative *Fusarium* strains (**Additional file 5 Figure 6**).

**Additional file 6. **Comparisons of synonymous site divergence estimates (*d*_*S*_) of concatenated nucleotide sequences of 13 housekeeping genes and the PKS, AT, and SDR genes from the *Fusarium* SAM clusters. Results for each SAM cluster are presented in a different sheet of the Excel file. Graphs shown in each spreadsheet show the *d*_*S*_ ratios; i.e., *d*_*S*_ value for SAM gene divided by *d*_*S*_ value for housekeeping genes.

**Additional file 7. **Information on putative SAM clusters in ascomycetous fungi other than *Fusarium*.

**Additional file 8. **Deletion analysis of the SAM5 PKS gene, *AOD1*, in *F. babinda* NRRL 25539 and *F. tricinctum* strain NRRL 25481.

**Additional file 9. **Proposed sphingofungin C biosynthetic pathway inferred by comparison of the chemical structure of sphingofungin C and the content of enzyme-encoding genes in the putative sphingofungin biosynthetic gene cluster. Proposed functions of genes in the putative sphingofungin biosynthetic gene cluster based on sequence homology to genes of known function (**Additional file 9 Table 1**). Arrangement of genes in the putative sphingofungin biosynthetic gene cluster. Arrows represent genes and point in the direction of transcription. The designations for the 13 genes in the cluster are Afu3g14670 – Afu3g14770, Afu3g14790 and Afug14800. However, only the last three digits of these designations are shown above the genes in the figure. Genes encoding the PKS, AT and SDR enzymes are indicated (**Additional file 9 Figure 1**). Proposed sphingofungin C biosynthetic pathway (**Additional file 9 Figure 2**).

**Additional file 10. ***Fusarium* strains examined in this study and sources of genome sequence data. NCAUR indicates USDA National Center for Agricultural Utilization Research, NCBI indicates National Center for Biotechnology Information, MIPS indicates Munich Information Center for Protein Sequences, and BGI indicates Beijing Genome Institute (Hong Kong).

**Additional file 11..** Housekeeping genes used to infer a species phylogeny. For trees shown in Fig. 5, all concatenated sequences of the coding regions of 13 housekeeping genes were used.

## Data Availability

Whole genome sequence data generated during this study have deposited at DDBJ/ENA/GenBank under accessions: JAADJF000000000, JAADJG000000000, JAADYS000000000, JAAFOW000000000, JAAGWO000000000, JAAGWP000000000, JAAGWQ000000000, JAALXH000000000, JAALXI000000000, JAALXJ000000000, JAALXK000000000, JAALXL000000000, JAALXM000000000, JAALXN000000000, JAAMOD000000000, JAANQP000000000, JAAOAG000000000, JAAOAH000000000, JAAOAI000000000, JAAOAJ000000000, JAAOAK000000000, JAAOAL000000000, JAAOAM000000000, JAAOAN000000000, JAAOAO000000000, JAAOAP000000000, JAAOAQ000000000, JAAOAR000000000, JAAOAS000000000, JAAOAT000000000, JAAOAU000000000, JAAOAV000000000, JAAOAW000000000, JAAOAX000000000, JAAOAY000000000, JAAQPE000000000, JAAQPF000000000, JAAQPG000000000, JAAQRH000000000, JAAQRI000000000, JAAQRM000000000, JABCJS000000000, JABCJT000000000, JABCJU000000000, JABCJV000000000, JABCJW000000000, JABCJX000000000, JABCJY000000000, JABCJZ000000000, JABCKB000000000, JABCKC000000000, JABCKD000000000, JABCKE000000000, JABCQV000000000, JABEEJ000000000, JABEEK000000000, JABEEL000000000, JABEEM000000000, JABEEN000000000, JABEEO000000000, JABEEP000000000, JABEEU000000000, JABELF000000000, JABEVY000000000, JABEXW000000000, JABEYC000000000, JABFAI000000000, JABFAK000000000, JABSTN000000000. As of June 2020, GenBank had not yet released data for some of the accession numbers indicated above. These data can be accessed via the following links: JAAOAI000000000: https://drive.google.com/file/d/1y7qGTaVCkDpHGes4rQGXItw0yxADWVTP/view?usp=sharing JAAOAJ000000000: https://drive.google.com/file/d/1upFEUzrqojnn_ZLV07j3vk31vyp6MfI8/view?usp=sharing

## Abbreviations

AAL toxin: *Alternaria alternate* f. sp. *lycopercisi* toxin
ALAS: Aminolevulinic acid synthase
AOD: 2-Amino-14,16-dimethyloctadecan-3-ol
AONS: Amino-7-oxononanoate synthase
ARS: Agriculture Research Service
AT: Aminotransferase
BLAST: Basic Local Alignment Search Tool
C: Carbon atom (e.g., C12 = carbon atom 12)
*d*_*S*_: Synonymous changes per synonymous site
FAS: Fatty acid synthase
FB: B-series fumonisin
FB_1_: Fumonisin B1
FB_2_: Fumonisin B2
FB_3_: Fumonisin B3
FB_4_: Fumonisin B4
FC: C-series fumonisin
*FUM*: Fumonisin biosynthetic gene
HGT: Horizontal gene transfer
HK: Housekeeping
LC: Liquid chromatography
LC-MS: Liquid chromatography-mass spectrometry
MIPS: Munich Information Center for Protein Sequences
MS: Mass spectrometry
NCAUR: National Center for Agricultural Utilization Research
NCBI: National Center for Biotechnology Information
NR: Non-redundant
NRPS: Nonribosomal peptide synthetase
PCR: Polymerase chain reaction
SAM: Sphinganine-analog metabolite
SDR: Short-chain dehydrogenase reductase
SH-AU: Shimodaira-Hasegawa and Approximately Unbiased
USDA: United States Department of Agriculture
