# Correction to: Identification and distribution of gene clusters required for synthesis of sphingolipid metabolism inhibitors in diverse species of the filamentous fungus *Fusarium*

**DOI:** 10.1186/s12864-020-07135-3

**Published:** 2020-10-15

**Authors:** Hye-Seon Kim, Jessica M. Lohmar, Mark Busman, Daren W. Brown, Todd A. Naumann, Hege H. Divon, Erik Lysøe, Silvio Uhlig, Robert H. Proctor

**Affiliations:** 1 U. S. Department of Agriculture, Agriculture Research Service, National Center for Agricultural Utilization Research, Peoria, IL USA; 2 Norwegian Veterinary Institute, Oslo, Norway; 3 Norwegian Institute of Bioeconomy Research, Ås, Norway

**Correction to: BMC Genomics 21, 510 (2020)**

**https://doi.org/10.1186/s12864-020-06896-1**

Following publication of the original article [1], the authors reported that in the original version of the manuscript, they did not include Erik Lysøe as a coauthor. He should have been included because of an important analysis that he did earlier in the study using his unpublished genome sequence data.

Erik Lysøe has been included in the authorship list, and the Authors’ Contribution declaration has been updated to include the contribution of Erik Lysøe.

The original article has been updated.
