# Supplementary material for: Identification and distribution of gene clusters required for synthesis of sphingolipid metabolism inhibitors in diverse species of the filamentous fungus Fusarium
Source: BMC Genomics. 2020 Jul 23;21:510. doi: 10.1186/s12864-020-06896-1 (PMC7376913; doi:10.1186/s12864-020-06896-1)
Supplement: Supplementary file 1 — Additional file 1. Phylogenies inferred by maximum likelihood analysis of alignment of predicted amino acid sequences of SAM polyketide synthase (PKS, Additional file 1 Figure 1), aminotransferase (AT, Additional file 1 Figure 2) and short-chain dehydrogenase reductase (SDR, Additional file 1 Figure 3) genes from Fusarium species and other ascomycetous fungi. The six phylogenetically distinct clades are color-coded according to genes in SAM cluster and labeled with known fumonisin and novel SAM1-SAM5 to the right of the tree. The prefix for protein designations for PKS, AT and SDR gene homologs from other ascomycetous fungi corresponding to NCBI GenBank accession are listed in Additional file 6 and the locus tag numbers for Fusarium genes are listed in Additional file 3. [file 12864_2020_6896_MOESM1_ESM.pptx]

## Slide 1
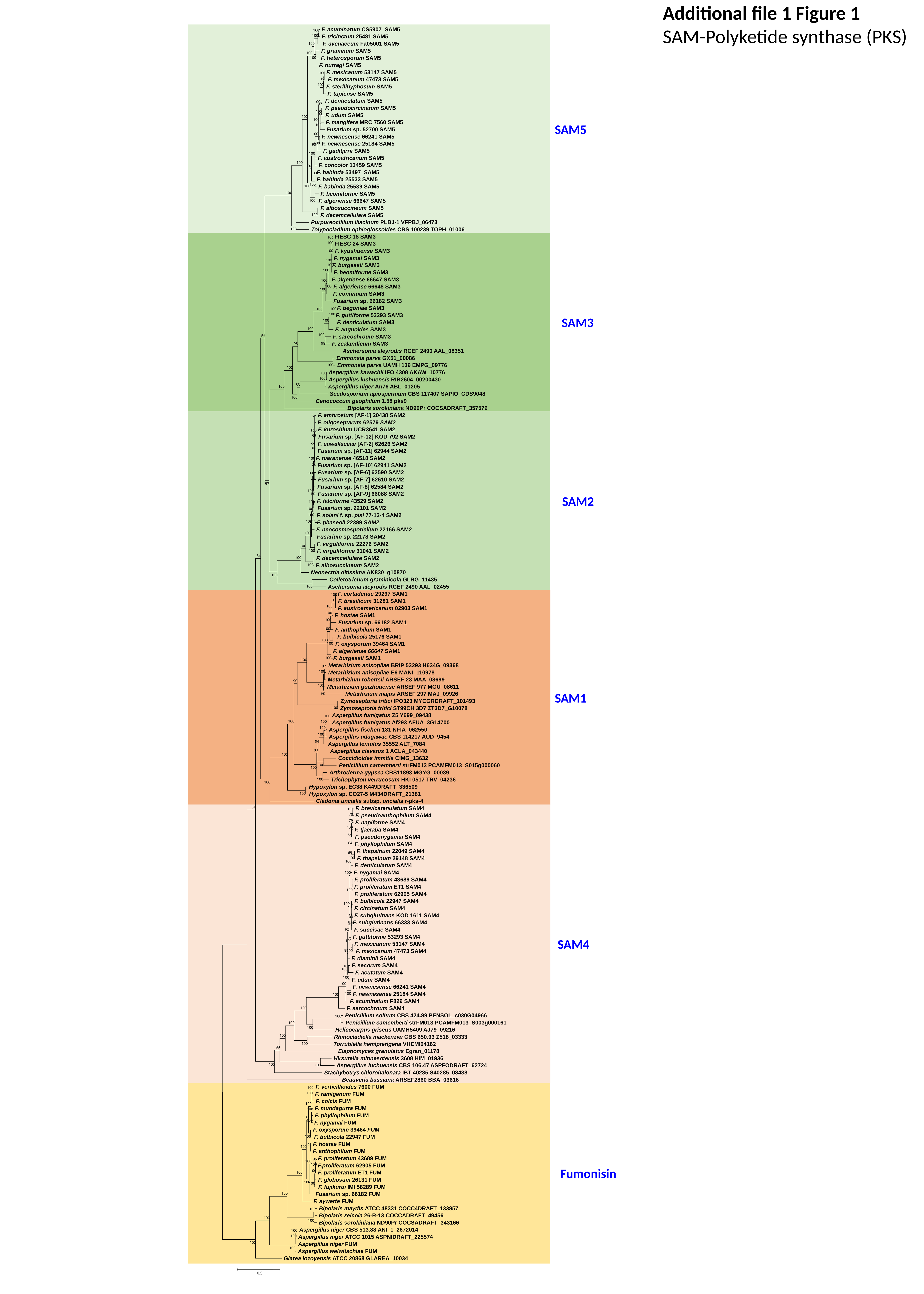

Additional file 1 Figure 1
SAM-Polyketide synthase (PKS)
 F. acuminatum CS5907 SAM5
 F. tricinctum 25481 SAM5
 F. avenaceum Fa05001 SAM5
 F. graminum SAM5
 F. heterosporum SAM5
 F. nurragi SAM5
 F. mexicanum 53147 SAM5
 F. mexicanum 47473 SAM5
 F. sterilihyphosum SAM5
 F. tupiense SAM5
 F. denticulatum SAM5
 F. pseudocircinatum SAM5
 F. udum SAM5
 F. mangifera MRC 7560 SAM5
 Fusarium sp. 52700 SAM5
 F. newnesense 66241 SAM5
 F. newnesense 25184 SAM5
 F. gaditjirrii SAM5
F. austroafricanum SAM5
 F. concolor 13459 SAM5
F. babinda 53497 SAM5
F. babinda 25533 SAM5
 F. babinda 25539 SAM5
 F. beomiforme SAM5
F. algeriense 66647 SAM5
 F. albosuccineum SAM5
 F. decemcellulare SAM5
 Purpureocillium lilacinum PLBJ-1 VFPBJ_06473
 Tolypocladium ophioglossoides CBS 100239 TOPH_01006
 FIESC 18 SAM3
 FIESC 24 SAM3
 F. kyushuense SAM3
 F. nygamai SAM3
F. burgessii SAM3
 F. beomiforme SAM3
F. algeriense 66647 SAM3
 F. algeriense 66648 SAM3
 F. continuum SAM3
 Fusarium sp. 66182 SAM3
 F. begoniae SAM3
F. guttiforme 53293 SAM3
 F. denticulatum SAM3
 F. anguoides SAM3
 F. sarcochroum SAM3
 F. zealandicum SAM3
 Aschersonia aleyrodis RCEF 2490 AAL_08351
 Emmonsia parva GX51_00086
 Emmonsia parva UAMH 139 EMPG_09776
 Aspergillus kawachii IFO 4308 AKAW_10776
 Aspergillus luchuensis RIB2604_00200430
 Aspergillus niger An76 ABL_01205
 Scedosporium apiospermum CBS 117407 SAPIO_CDS9048
 Cenococcum geophilum 1.58 pks9
 Bipolaris sorokiniana ND90Pr COCSADRAFT_357579
 F. ambrosium [AF-1] 20438 SAM2
 F. oligoseptarum 62579 SAM2
 F. kuroshium UCR3641 SAM2
 Fusarium sp. [AF-12] KOD 792 SAM2
 F. euwallaceae [AF-2] 62626 SAM2
 Fusarium sp. [AF-11] 62944 SAM2
F. tuaranense 46518 SAM2
 Fusarium sp. [AF-10] 62941 SAM2
 Fusarium sp. [AF-6] 62590 SAM2
 Fusarium sp. [AF-7] 62610 SAM2
 Fusarium sp. [AF-8] 62584 SAM2
 Fusarium sp. [AF-9] 66088 SAM2
 F. falciforme 43529 SAM2
 Fusarium sp. 22101 SAM2
100
100
100
100
100
100
96
100
100
87
100
83
100
100
100
100
100
90
100
100
100
100
100
100
100
100
100
100
100
100
100
100
100
100
100
100
100
100
100
100
100
100
100
84
98
95
100
100
100
100
83
100
100
62
81
100
93
99
100
100
74
100
41
97
100
98
100
100
100
100
100
100
100
100
100
100
100
100
100
100
100
100
100
 F. newnesense 66241 SAM4
 F. newnesense 25184 SAM4
 F. acuminatum F829 SAM4
 F. sarcochroum SAM4
 Penicillium solitum CBS 424.89 PENSOL_c030G04966
 Penicillium camemberti strFM013 PCAMFM013_S003g000161
 Helicocarpus griseus UAMH5409 AJ79_09216
 Rhinocladiella mackenziei CBS 650.93 Z518_03333
 Torrubiella hemipterigena VHEMI04162
 Elaphomyces granulatus Egran_01178
 Hirsutella minnesotensis 3608 HIM_01936
 Aspergillus luchuensis CBS 106.47 ASPFODRAFT_62724
 Stachybotrys chlorohalonata IBT 40285 S40285_08438
 Beauveria bassiana ARSEF2860 BBA_03616
 F. verticillioides 7600 FUM
 F. ramigenum FUM
 F. coicis FUM
 F. mundagurra FUM
 F. phyllophilum FUM
 F. nygamai FUM
 F. oxysporum 39464 FUM
 F. bulbicola 22947 FUM
 F. hostae FUM
 F. anthophilum FUM
 F. proliferatum 43689 FUM
 F.proliferatum 62905 FUM
 F. proliferatum ET1 FUM
 F. globosum 26131 FUM
 F. fujikuroi IMI 58289 FUM
 Fusarium sp. 66182 FUM
 F. aywerte FUM
 Bipolaris maydis ATCC 48331 COCC4DRAFT_133857
 Bipolaris zeicola 26-R-13 COCCADRAFT_49456
 Bipolaris sorokiniana ND90Pr COCSADRAFT_343166
 Aspergillus niger CBS 513.88 ANI_1_2672014
 Aspergillus niger ATCC 1015 ASPNIDRAFT_225574
 Aspergillus niger FUM
 Aspergillus welwitschiae FUM
 Glarea lozoyensis ATCC 20868 GLAREA_10034
F. solani f. sp. pisi 77-13-4 SAM2
F. phaseoli 22389 SAM2
 F. neocosmosporiellum 22166 SAM2
 Fusarium sp. 22178 SAM2
 F. virguliforme 22276 SAM2
 F. virguliforme 31041 SAM2
 F. decemcellulare SAM2
 F. albosuccineum SAM2
 Neonectria ditissima AK830_g10870
 Colletotrichum graminicola GLRG_11435
 Aschersonia aleyrodis RCEF 2490 AAL_02455
 F. cortaderiae 29297 SAM1
 F. brasilicum 31281 SAM1
 F. austroamericanum 02903 SAM1
 F. hostae SAM1
 Fusarium sp. 66182 SAM1
 F. anthophilum SAM1
 F. bulbicola 25176 SAM1
 F. oxysporum 39464 SAM1
F. algeriense 66647 SAM1
F. burgessii SAM1
 Metarhizium anisopliae BRIP 53293 H634G_09368
 Metarhizium anisopliae E6 MANI_110978
 Metarhizium robertsii ARSEF 23 MAA_08699
 Metarhizium guizhouense ARSEF 977 MGU_08611
 Metarhizium majus ARSEF 297 MAJ_09926
 Zymoseptoria tritici IPO323 MYCGRDRAFT_101493
 Zymoseptoria tritici ST99CH 3D7 ZT3D7_G10078
 Aspergillus fumigatus Z5 Y699_09438
 Aspergillus fumigatus Af293 AFUA_3G14700
 Aspergillus fischeri 181 NFIA_062550
 Aspergillus udagawae CBS 114217 AUD_9454
 Aspergillus lentulus 35552 ALT_7084
 Aspergillus clavatus 1 ACLA_043440
 Coccidioides immitis CIMG_13632
 Penicillium camemberti strFM013 PCAMFM013_S015g000060
 Arthroderma gypsea CBS11893 MGYG_00039
 Trichophyton verrucosum HKI 0517 TRV_04236
 Hypoxylon sp. EC38 K449DRAFT_336509
 Hypoxylon sp. CO27-5 M434DRAFT_21381
 Cladonia uncialis subsp. uncialis r-pks-4
 F. brevicatenulatum SAM4
 F. pseudoanthophilum SAM4
 F. napiforme SAM4
 F. tjaetaba SAM4
 F. pseudonygamai SAM4
 F. phyllophilum SAM4
 F. thapsinum 22049 SAM4
 F. thapsinum 29148 SAM4
 F. denticulatum SAM4
 F. nygamai SAM4
 F. proliferatum 43689 SAM4
 F. proliferatum ET1 SAM4
 F. proliferatum 62905 SAM4
 F. bulbicola 22947 SAM4
 F. circinatum SAM4
 F. subglutinans KOD 1611 SAM4
F. subglutinans 66333 SAM4
 F. succisae SAM4
F. guttiforme 53293 SAM4
 F. mexicanum 53147 SAM4
 F. mexicanum 47473 SAM4
 F. dlaminii SAM4
 F. secorum SAM4
 F. acutatum SAM4
 F. udum SAM4
84
100
100
100
100
100
97
100
90
100
98
100
100
100
100
100
100
94
93
100
100
100
100
100
100
61
100
79
79
100
64
64
69
100
100
100
100
100
49
98
100
100
51
92
100
99
100
100
100
100
100
100
100
100
100
100
100
100
100
99
100
100
100
100
100
100
100
100
100
99
100
96
100
100
100
100
100
100
100
100
100
100
100
100
100
100
0.5
SAM5
SAM3
SAM2
SAM1
SAM4
Fumonisin

## Slide 2
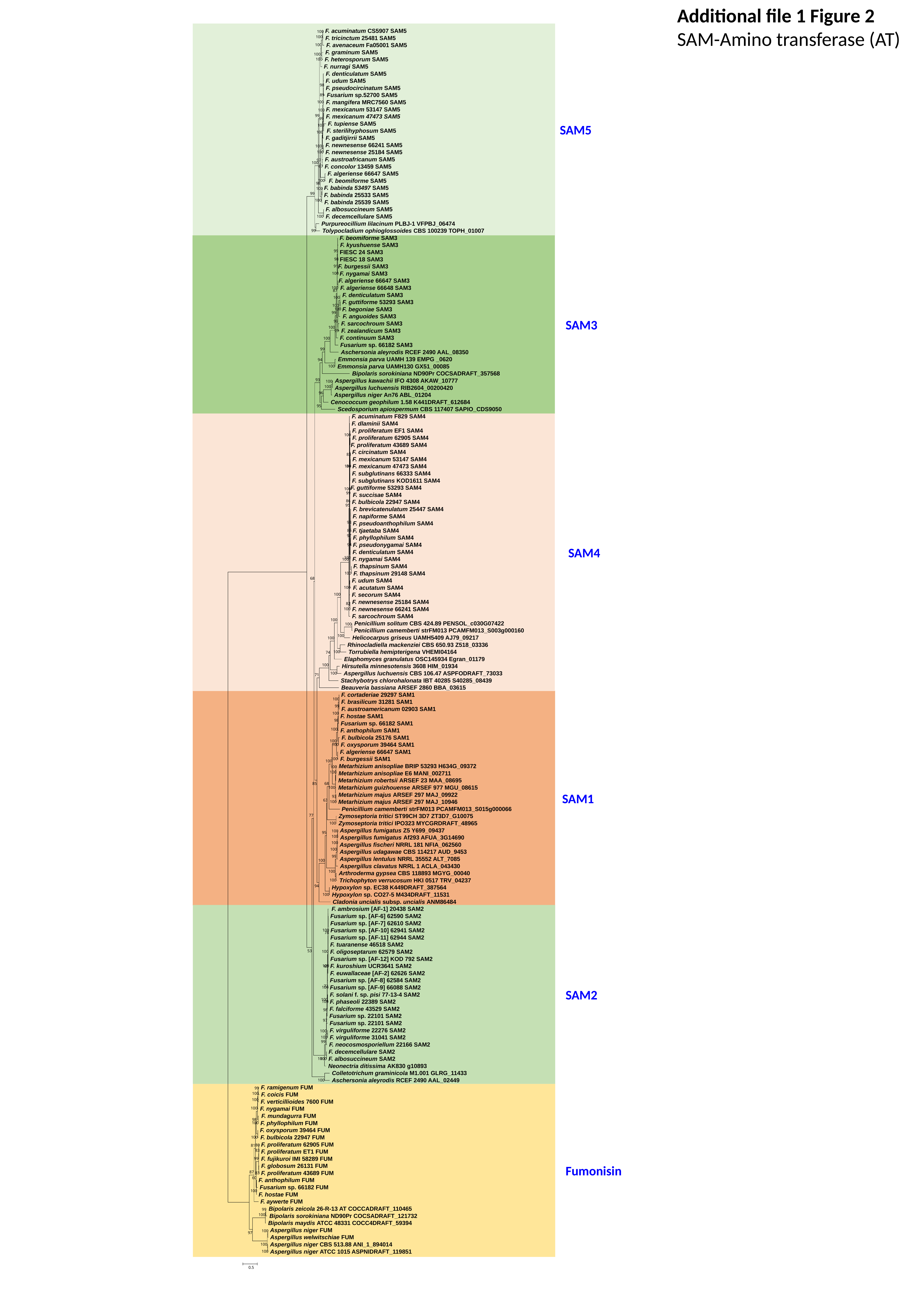

Additional file 1 Figure 2
SAM-Amino transferase (AT)
 F. acuminatum CS5907 SAM5
 F. tricinctum 25481 SAM5
 F. avenaceum Fa05001 SAM5
 F. graminum SAM5
 F. heterosporum SAM5
 F. nurragi SAM5
 F. denticulatum SAM5
 F. udum SAM5
 F. pseudocircinatum SAM5
 Fusarium sp.52700 SAM5
 F. mangifera MRC7560 SAM5
 F. mexicanum 53147 SAM5
 F. mexicanum 47473 SAM5
 F. tupiense SAM5
 F. sterilihyphosum SAM5
 F. gaditjirrii SAM5
 F. newnesense 66241 SAM5
 F. newnesense 25184 SAM5
 F. austroafricanum SAM5
 F. concolor 13459 SAM5
 F. algeriense 66647 SAM5
 F. beomiforme SAM5
 F. babinda 53497 SAM5
 F. babinda 25533 SAM5
 F. babinda 25539 SAM5
 F. albosuccineum SAM5
 F. decemcellulare SAM5
 Purpureocillium lilacinum PLBJ-1 VFPBJ_06474
 Tolypocladium ophioglossoides CBS 100239 TOPH_01007
 F. beomiforme SAM3
 F. kyushuense SAM3
 FIESC 24 SAM3
 FIESC 18 SAM3
F. burgessii SAM3
 F. nygamai SAM3
F. algeriense 66647 SAM3
 F. algeriense 66648 SAM3
 F. denticulatum SAM3
 F. guttiforme 53293 SAM3
 F. begoniae SAM3
 F. anguoides SAM3
 F. sarcochroum SAM3
 F. zealandicum SAM3
 F. continuum SAM3
 Fusarium sp. 66182 SAM3
 Aschersonia aleyrodis RCEF 2490 AAL_08350
 Emmonsia parva UAMH 139 EMPG _0620
 Emmonsia parva UAMH130 GX51_00085
 Bipolaris sorokiniana ND90Pr COCSADRAFT_357568
 Aspergillus kawachii IFO 4308 AKAW_10777
 Aspergillus luchuensis RIB2604_00200420
 Aspergillus niger An76 ABL_01204
 Cenococcum geophilum 1.58 K441DRAFT_612684
 Scedosporium apiospermum CBS 117407 SAPIO_CDS9050
 F. acuminatum F829 SAM4
 F. dlaminii SAM4
 F. proliferatum EF1 SAM4
 F. proliferatum 62905 SAM4
F. proliferatum 43689 SAM4
 F. circinatum SAM4
 F. mexicanum 53147 SAM4
 F. mexicanum 47473 SAM4
 F. subglutinans 66333 SAM4
 F. subglutinans KOD1611 SAM4
F. guttiforme 53293 SAM4
 F. succisae SAM4
 F. bulbicola 22947 SAM4
 F. brevicatenulatum 25447 SAM4
 F. napiforme SAM4
 F. pseudoanthophilum SAM4
 F. tjaetaba SAM4
100
100
100
100
100
98
89
100
100
99
99
100
100
100
100
97
100
67
100
98
100
99
100
100
99
99
98
91
100
100
87
100
100
100
99
99
100
83
86
100
100
99
86
95
98
85
97
94
100
100
100
100
100
82
100
100
100
100
100
100
74
100
100
71
100
99
100
98
100
100
100
100
100
100
100
85
68
100
93
63
100
100
100
95
100
100
100
99
100
100
100
94
100
 F. falciforme 43529 SAM2
 Fusarium sp. 22101 SAM2
 Fusarium sp. 22101 SAM2
 F. virguliforme 22276 SAM2
 F. virguliforme 31041 SAM2
 F. neocosmosporiellum 22166 SAM2
 F. decemcellulare SAM2
 F. albosuccineum SAM2
 Neonectria ditissima AK830 g10893
 Colletotrichum graminicola M1.001 GLRG_11433
 Aschersonia aleyrodis RCEF 2490 AAL_02449
 F. ramigenum FUM
 F. coicis FUM
 F. verticillioides 7600 FUM
 F. nygamai FUM
 F. mundagurra FUM
 F. phyllophilum FUM
 F. oxysporum 39464 FUM
 F. bulbicola 22947 FUM
 F. proliferatum 62905 FUM
 F. proliferatum ET1 FUM
 F. fujikuroi IMI 58289 FUM
 F. globosum 26131 FUM
 F. proliferatum 43689 FUM
 F. anthophilum FUM
 Fusarium sp. 66182 FUM
 F. hostae FUM
 F. aywerte FUM
 Bipolaris zeicola 26-R-13 AT COCCADRAFT_110465
 Bipolaris sorokiniana ND90Pr COCSADRAFT_121732
 Bipolaris maydis ATCC 48331 COCC4DRAFT_59394
 Aspergillus niger FUM
 Aspergillus welwitschiae FUM
 Aspergillus niger CBS 513.88 ANI_1_894014
 Aspergillus niger ATCC 1015 ASPNIDRAFT_119851
99
100
100
99
94
100
93
100
100
96
95
 F. phyllophilum SAM4
 F. pseudonygamai SAM4
 F. denticulatum SAM4
 F. nygamai SAM4
 F. thapsinum SAM4
 F. thapsinum 29148 SAM4
 F. udum SAM4
 F. acutatum SAM4
 F. secorum SAM4
 F. newnesense 25184 SAM4
 F. newnesense 66241 SAM4
 F. sarcochroum SAM4
 Penicillium solitum CBS 424.89 PENSOL_c030G07422
 Penicillium camemberti strFM013 PCAMFM013_S003g000160
 Helicocarpus griseus UAMH5409 AJ79_09217
 Rhinocladiella mackenziei CBS 650.93 Z518_03336
 Torrubiella hemipterigena VHEMI04164
 Elaphomyces granulatus OSC145934 Egran_01179
 Hirsutella minnesotensis 3608 HIM_01934
 Aspergillus luchuensis CBS 106.47 ASPFODRAFT_73033
 Stachybotrys chlorohalonata IBT 40285 S40285_08439
 Beauveria bassiana ARSEF 2860 BBA_03615
 F. cortaderiae 29297 SAM1
 F. brasilicum 31281 SAM1
 F. austroamericanum 02903 SAM1
 F. hostae SAM1
 Fusarium sp. 66182 SAM1
 F. anthophilum SAM1
 F. bulbicola 25176 SAM1
 F. oxysporum 39464 SAM1
 F. algeriense 66647 SAM1
 F. burgessii SAM1
 Metarhizium anisopliae BRIP 53293 H634G_09372
 Metarhizium anisopliae E6 MANI_002711
 Metarhizium robertsii ARSEF 23 MAA_08695
 Metarhizium guizhouense ARSEF 977 MGU_08615
 Metarhizium majus ARSEF 297 MAJ_09922
 Metarhizium majus ARSEF 297 MAJ_10946
 Penicillium camemberti strFM013 PCAMFM013_S015g000066
 Zymoseptoria tritici ST99CH 3D7 ZT3D7_G10075
 Zymoseptoria tritici IPO323 MYCGRDRAFT_48965
 Aspergillus fumigatus Z5 Y699_09437
 Aspergillus fumigatus Af293 AFUA_3G14690
 Aspergillus fischeri NRRL 181 NFIA_062560
 Aspergillus udagawae CBS 114217 AUD_9453
 Aspergillus lentulus NRRL 35552 ALT_7085
 Aspergillus clavatus NRRL 1 ACLA_043430
 Arthroderma gypsea CBS 118893 MGYG_00040
 Trichophyton verrucosum HKI 0517 TRV_04237
 Hypoxylon sp. EC38 K449DRAFT_387564
 Hypoxylon sp. CO27-5 M434DRAFT_11531
 Cladonia uncialis subsp. uncialis ANM86484
 F. ambrosium [AF-1] 20438 SAM2
 Fusarium sp. [AF-6] 62590 SAM2
 Fusarium sp. [AF-7] 62610 SAM2
 Fusarium sp. [AF-10] 62941 SAM2
 Fusarium sp. [AF-11] 62944 SAM2
 F. tuaranense 46518 SAM2
 F. oligoseptarum 62579 SAM2
 Fusarium sp. [AF-12] KOD 792 SAM2
 F. kuroshium UCR3641 SAM2
 F. euwallaceae [AF-2] 62626 SAM2
 Fusarium sp. [AF-8] 62584 SAM2
 Fusarium sp. [AF-9] 66088 SAM2
 F. solani f. sp. pisi 77-13-4 SAM2
 F. phaseoli 22389 SAM2
68
77
100
78
53
100
100
65
72
100
100
100
98
97
100
100
99
100
100
100
99
100
100
100
98
100
100
80
81
83
99
87
83
60
100
99
100
100
97
100
100
0.5
SAM5
SAM3
SAM4
SAM1
SAM2
Fumonisin

## Slide 3
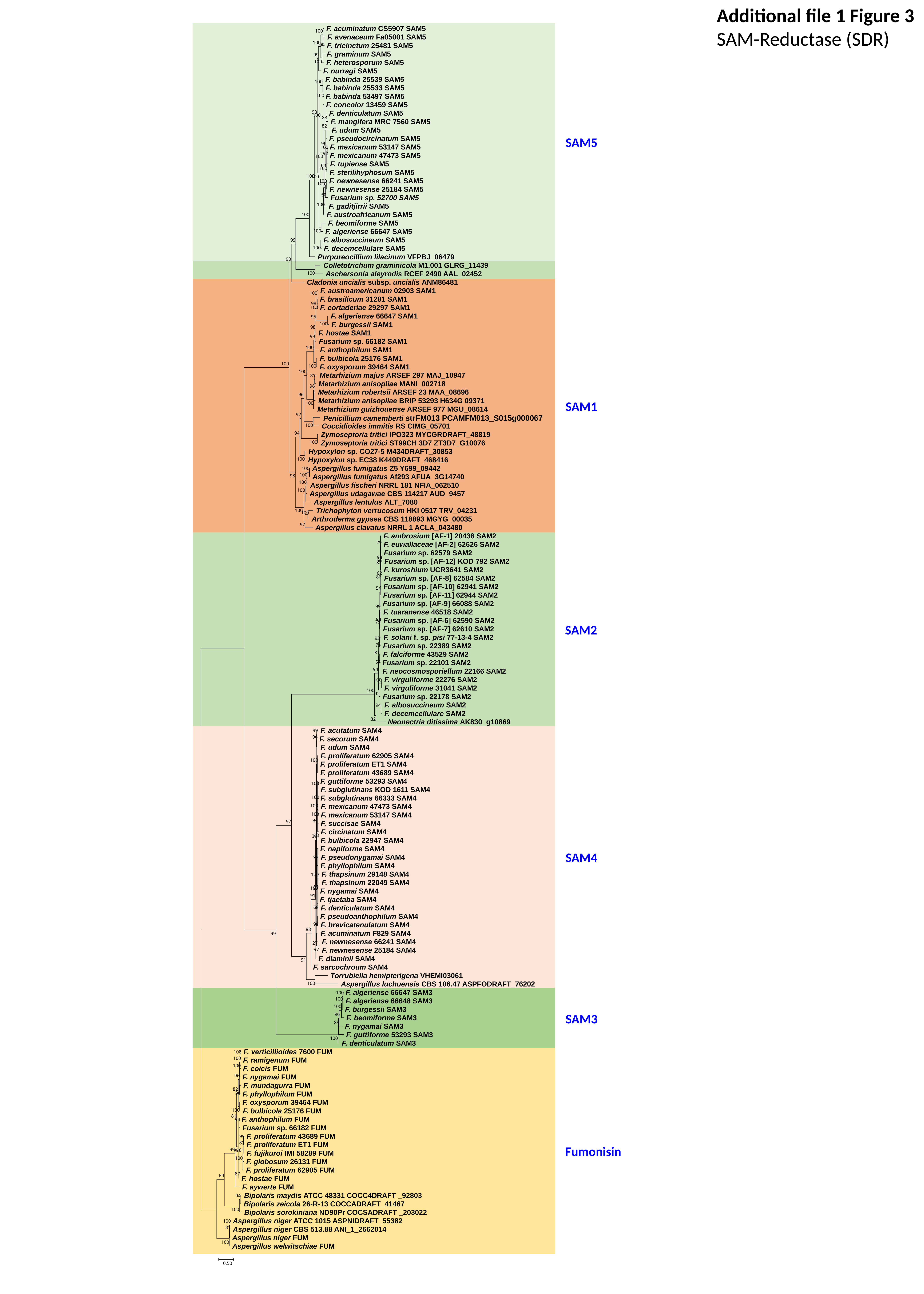

Additional file 1 Figure 3
SAM-Reductase (SDR)
 F. acuminatum CS5907 SAM5
 F. avenaceum Fa05001 SAM5
 F. tricinctum 25481 SAM5
 F. graminum SAM5
 F. heterosporum SAM5
 F. nurragi SAM5
 F. babinda 25539 SAM5
 F. babinda 25533 SAM5
 F. babinda 53497 SAM5
 F. concolor 13459 SAM5
 F. denticulatum SAM5
 F. mangifera MRC 7560 SAM5
 F. udum SAM5
 F. pseudocircinatum SAM5
 F. mexicanum 53147 SAM5
 F. mexicanum 47473 SAM5
 F. tupiense SAM5
 F. sterilihyphosum SAM5
 F. newnesense 66241 SAM5
 F. newnesense 25184 SAM5
 Fusarium sp. 52700 SAM5
 F. gaditjirrii SAM5
 F. austroafricanum SAM5
 F. beomiforme SAM5
 F. algeriense 66647 SAM5
 F. albosuccineum SAM5
 F. decemcellulare SAM5
 Purpureocillium lilacinum VFPBJ_06479
 Colletotrichum graminicola M1.001 GLRG_11439
 Aschersonia aleyrodis RCEF 2490 AAL_02452
 Cladonia uncialis subsp. uncialis ANM86481
 F. austroamericanum 02903 SAM1
 F. brasilicum 31281 SAM1
 F. cortaderiae 29297 SAM1
 F. algeriense 66647 SAM1
 F. burgessii SAM1
 F. hostae SAM1
 Fusarium sp. 66182 SAM1
 F. anthophilum SAM1
 F. bulbicola 25176 SAM1
 F. oxysporum 39464 SAM1
 Metarhizium majus ARSEF 297 MAJ_10947
 Metarhizium anisopliae MANI_002718
 Metarhizium robertsii ARSEF 23 MAA_08696
 Metarhizium anisopliae BRIP 53293 H634G 09371
 Metarhizium guizhouense ARSEF 977 MGU_08614
 Penicillium camemberti strFM013 PCAMFM013_S015g000067
 Coccidioides immitis RS CIMG_05701
 Zymoseptoria tritici IPO323 MYCGRDRAFT_48819
 Zymoseptoria tritici ST99CH 3D7 ZT3D7_G10076
 Hypoxylon sp. CO27-5 M434DRAFT_30853
 Hypoxylon sp. EC38 K449DRAFT_468416
 Aspergillus fumigatus Z5 Y699_09442
 Aspergillus fumigatus Af293 AFUA_3G14740
 Aspergillus fischeri NRRL 181 NFIA_062510
 Aspergillus udagawae CBS 114217 AUD_9457
 Aspergillus lentulus ALT_7080
 Trichophyton verrucosum HKI 0517 TRV_04231
 Arthroderma gypsea CBS 118893 MGYG_00035
 Aspergillus clavatus NRRL 1 ACLA_043480
 F. ambrosium [AF-1] 20438 SAM2
 F. euwallaceae [AF-2] 62626 SAM2
 Fusarium sp. 62579 SAM2
 Fusarium sp. [AF-12] KOD 792 SAM2
 F. kuroshium UCR3641 SAM2
 Fusarium sp. [AF-8] 62584 SAM2
 Fusarium sp. [AF-10] 62941 SAM2
 Fusarium sp. [AF-11] 62944 SAM2
100
100
100
99
100
100
100
99
100
83
82
96
100
92
100
64
100
100
100
100
100
50
100
100
100
99
100
90
100
100
98
100
95
100
98
99
100
100
100
100
81
96
96
100
92
100
94
100
100
100
100
98
100
100
100
100
97
29
98
56
82
62
86
 Fusarium sp. [AF-9] 66088 SAM2
 F. tuaranense 46518 SAM2
 Fusarium sp. [AF-6] 62590 SAM2
 Fusarium sp. [AF-7] 62610 SAM2
 F. solani f. sp. pisi 77-13-4 SAM2
 Fusarium sp. 22389 SAM2
 F. falciforme 43529 SAM2
 Fusarium sp. 22101 SAM2
 F. neocosmosporiellum 22166 SAM2
 F. virguliforme 22276 SAM2
 F. virguliforme 31041 SAM2
 Fusarium sp. 22178 SAM2
 F. albosuccineum SAM2
 F. decemcellulare SAM2
 Neonectria ditissima AK830_g10869
 F. acutatum SAM4
 F. secorum SAM4
 F. udum SAM4
 F. proliferatum 62905 SAM4
 F. proliferatum ET1 SAM4
 F. proliferatum 43689 SAM4
 F. guttiforme 53293 SAM4
 F. subglutinans KOD 1611 SAM4
 F. subglutinans 66333 SAM4
 F. mexicanum 47473 SAM4
 F. mexicanum 53147 SAM4
 F. succisae SAM4
 F. circinatum SAM4
 F. bulbicola 22947 SAM4
 F. napiforme SAM4
 F. pseudonygamai SAM4
 F. phyllophilum SAM4
 F. thapsinum 29148 SAM4
 F. thapsinum 22049 SAM4
 F. nygamai SAM4
 F. tjaetaba SAM4
 F. denticulatum SAM4
 F. pseudoanthophilum SAM4
 F. brevicatenulatum SAM4
 F. acuminatum F829 SAM4
 F. newnesense 66241 SAM4
 F. newnesense 25184 SAM4
 F. dlaminii SAM4
F. sarcochroum SAM4
 Torrubiella hemipterigena VHEMI03061
 Aspergillus luchuensis CBS 106.47 ASPFODRAFT_76202
 F. algeriense 66647 SAM3
 F. algeriense 66648 SAM3
 F. burgessii SAM3
 F. beomiforme SAM3
 F. nygamai SAM3
 F. guttiforme 53293 SAM3
 F. denticulatum SAM3
 F. verticillioides 7600 FUM
 F. ramigenum FUM
 F. coicis FUM
 F. nygamai FUM
 F. mundagurra FUM
 F. phyllophilum FUM
 F. oxysporum 39464 FUM
 F. bulbicola 25176 FUM
 F. anthophilum FUM
 Fusarium sp. 66182 FUM
 F. proliferatum 43689 FUM
 F. proliferatum ET1 FUM
 F. fujikuroi IMI 58289 FUM
 F. globosum 26131 FUM
54
99
59
79
93
75
81
64
94
100
100
92
94
82
99
96
100
100
100
100
100
94
97
98
36
97
100
87
100
91
68
98
88
99
27
97
91
100
100
100
100
96
88
100
100
100
100
96
82
96
100
81
94
99
82
99
81
99
100
 F. proliferatum 62905 FUM
87
69
 F. hostae FUM
 F. aywerte FUM
 Bipolaris maydis ATCC 48331 COCC4DRAFT _92803
94
 Bipolaris zeicola 26-R-13 COCCADRAFT_41467
100
 Bipolaris sorokiniana ND90Pr COCSADRAFT _203022
 Aspergillus niger ATCC 1015 ASPNIDRAFT_55382
100
81
 Aspergillus niger CBS 513.88 ANI_1_2662014
 Aspergillus niger FUM
100
 Aspergillus welwitschiae FUM
0.50
SAM5
SAM1
SAM2
SAM4
SAM3
Fumonisin
