# Supplementary material for: Identification and distribution of gene clusters required for synthesis of sphingolipid metabolism inhibitors in diverse species of the filamentous fungus Fusarium
Source: BMC Genomics. 2020 Jul 23;21:510. doi: 10.1186/s12864-020-06896-1 (PMC7376913; doi:10.1186/s12864-020-06896-1)
Supplement: Supplementary file 2 — Additional file 2. Organization of the genes and flanking genes in putative SAM clusters: SAM2 (Additional file 2 Figure 1), SAM3 (Additional file 2 Figure 2), SAM5 (Additional file 2 Figure 3) and FUM (Additional file 2 Figure 4) from two representative Fusarium strains (red-outlined box) in phylogenetic tree inferred by maximum likelihood analysis of nucleotide sequences of PKS gene coding regions with 1000 bootstrap. Colored arrows represent genes and flanking genes of each SAM clusters. The direction of the arrows indicates direction of gene transcriptions. Yellow arrows indicate PKS, SDR and AT genes; and colors indicate genes predicted to have other functions based on sequence homology. The prefixes for locus tag number of gene or flanking gene designations corresponding to each SAM clusters are listed in Additional file 3. [file 12864_2020_6896_MOESM2_ESM.pptx]

## Slide 1
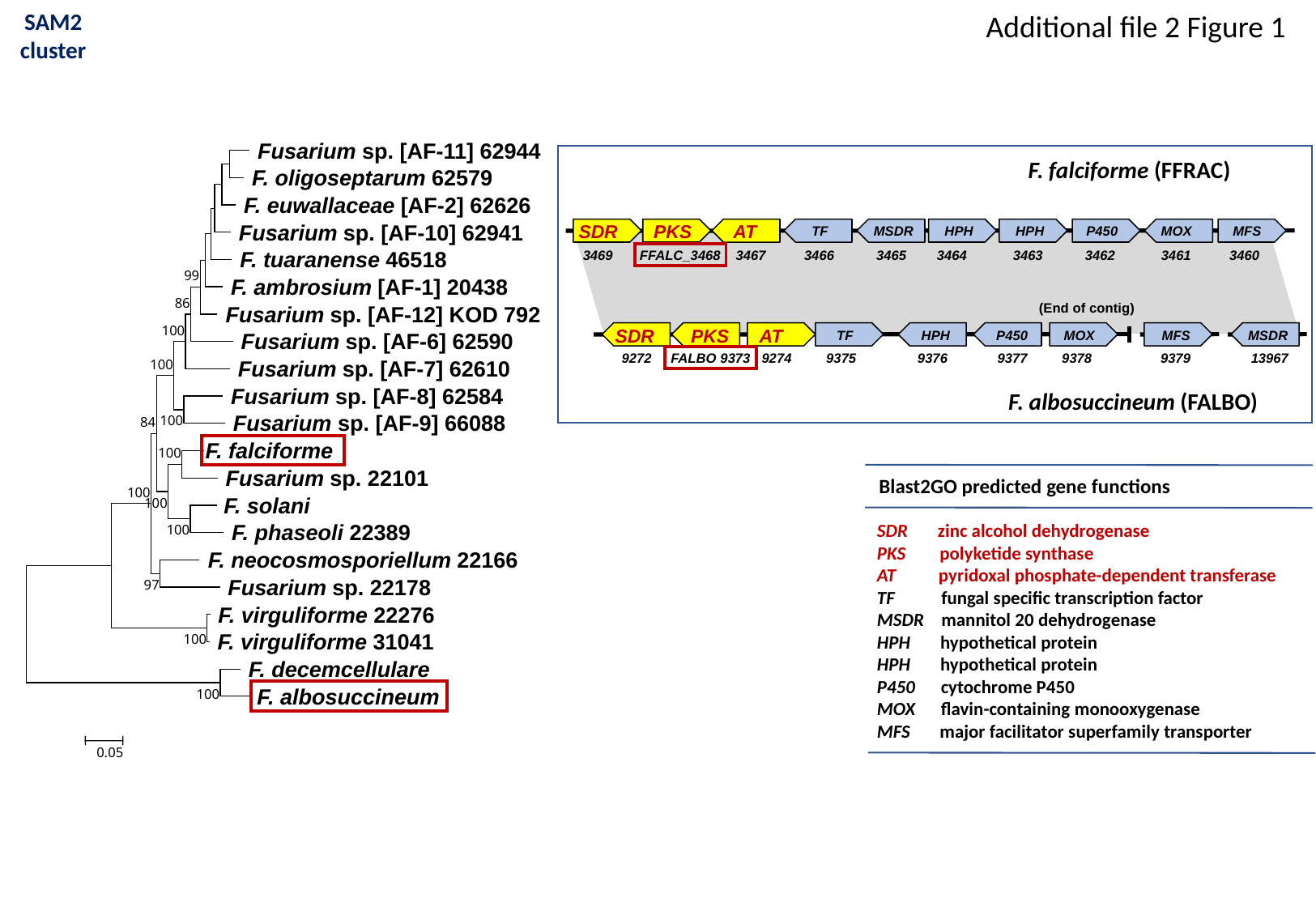

SAM2 cluster
Additional file 2 Figure 1
 Fusarium sp. [AF-11] 62944
 F. oligoseptarum 62579
 F. euwallaceae [AF-2] 62626
 Fusarium sp. [AF-10] 62941
 F. tuaranense 46518
99
 F. ambrosium [AF-1] 20438
86
 Fusarium sp. [AF-12] KOD 792
100
 Fusarium sp. [AF-6] 62590
 Fusarium sp. [AF-7] 62610
100
 Fusarium sp. [AF-8] 62584
 Fusarium sp. [AF-9] 66088
100
84
F. falciforme
100
 Fusarium sp. 22101
100
 F. solani
100
 F. phaseoli 22389
100
 F. neocosmosporiellum 22166
 Fusarium sp. 22178
97
 F. virguliforme 22276
 F. virguliforme 31041
100
 F. decemcellulare
 F. albosuccineum
100
0.05
F. falciforme (FFRAC)
SDR
PKS
AT
AT
TF
MSDR
HPH
HPH
P450
MOX
MFS
3469 FFALC_3468 3467 3466 3465 3464 3463 3462 3461 3460
(End of contig)
SDR
PKS
AT
TF
HPH
P450
MOX
MFS
MSDR
9272 FALBO 9373 9274 9375 9376 9377 9378
9379
13967
F. albosuccineum (FALBO)
SDR zinc alcohol dehydrogenase
PKS polyketide synthase
AT pyridoxal phosphate-dependent transferase
TF fungal specific transcription factor
MSDR mannitol 20 dehydrogenase
HPH hypothetical protein
HPH hypothetical protein
P450 cytochrome P450
MOX flavin-containing monooxygenase
MFS major facilitator superfamily transporter
Blast2GO predicted gene functions

## Slide 2
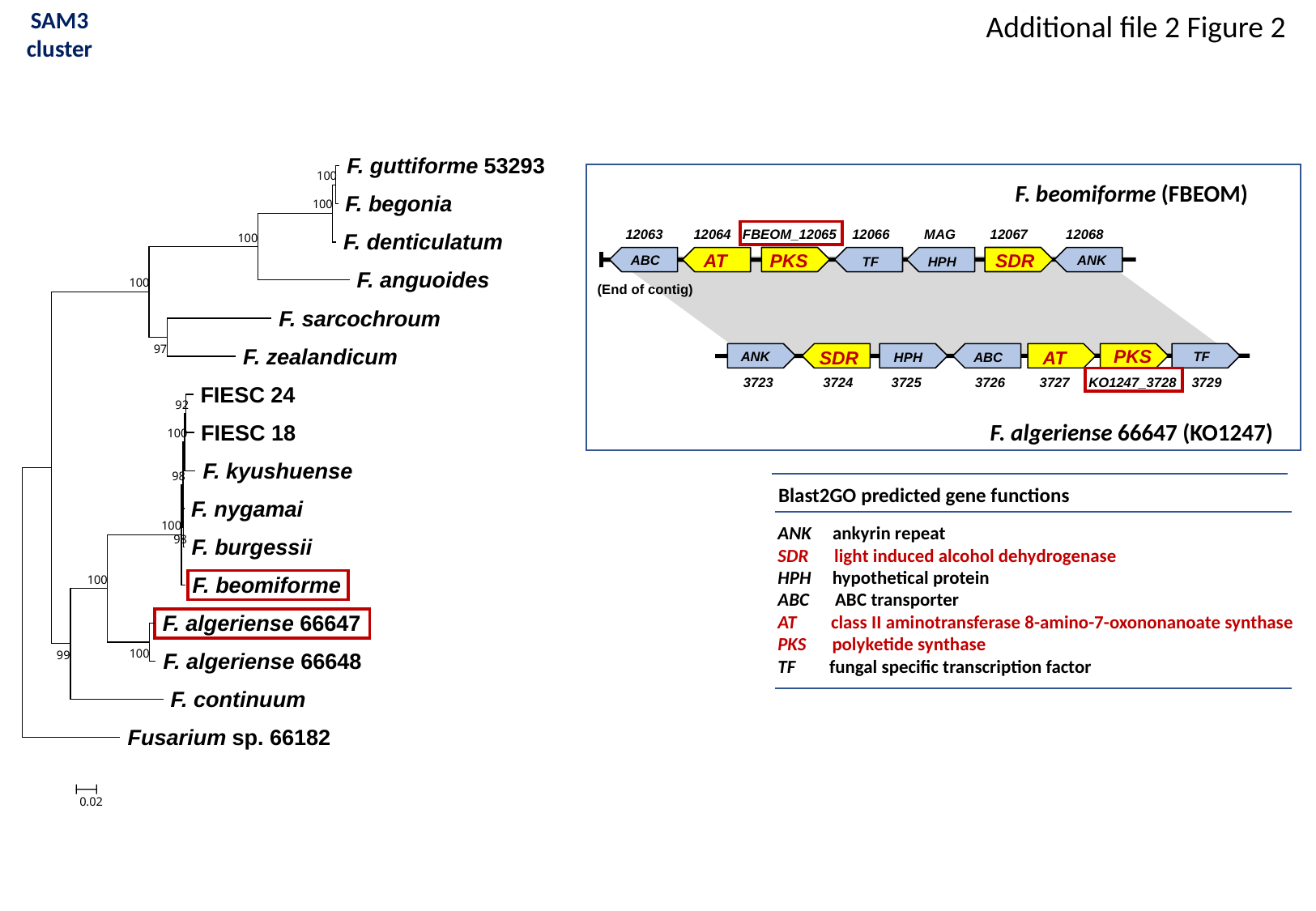

SAM3 cluster
Additional file 2 Figure 2
 F. guttiforme 53293
100
 F. begonia
100
 F. denticulatum
100
 F. anguoides
100
 F. sarcochroum
97
 F. zealandicum
 FIESC 24
92
 FIESC 18
100
 F. kyushuense
98
 F. nygamai
100
98
 F. burgessii
 F. beomiforme
100
 F. algeriense 66647
100
 F. algeriense 66648
99
 F. continuum
 Fusarium sp. 66182
0.02
F. beomiforme (FBEOM)
 12063 12064 FBEOM_12065 12066 MAG 12067 12068
AT
PKS
SDR
ABC
ANK
TF
HPH
(End of contig)
PKS
SDR
AT
ANK
TF
ABC
HPH
3723 3724 3725 3726 3727 KO1247_3728 3729
F. algeriense 66647 (KO1247)
Blast2GO predicted gene functions
ANK ankyrin repeat
SDR light induced alcohol dehydrogenase
HPH hypothetical protein
ABC ABC transporter
AT class II aminotransferase 8-amino-7-oxononanoate synthase
PKS polyketide synthase
TF fungal specific transcription factor

## Slide 3
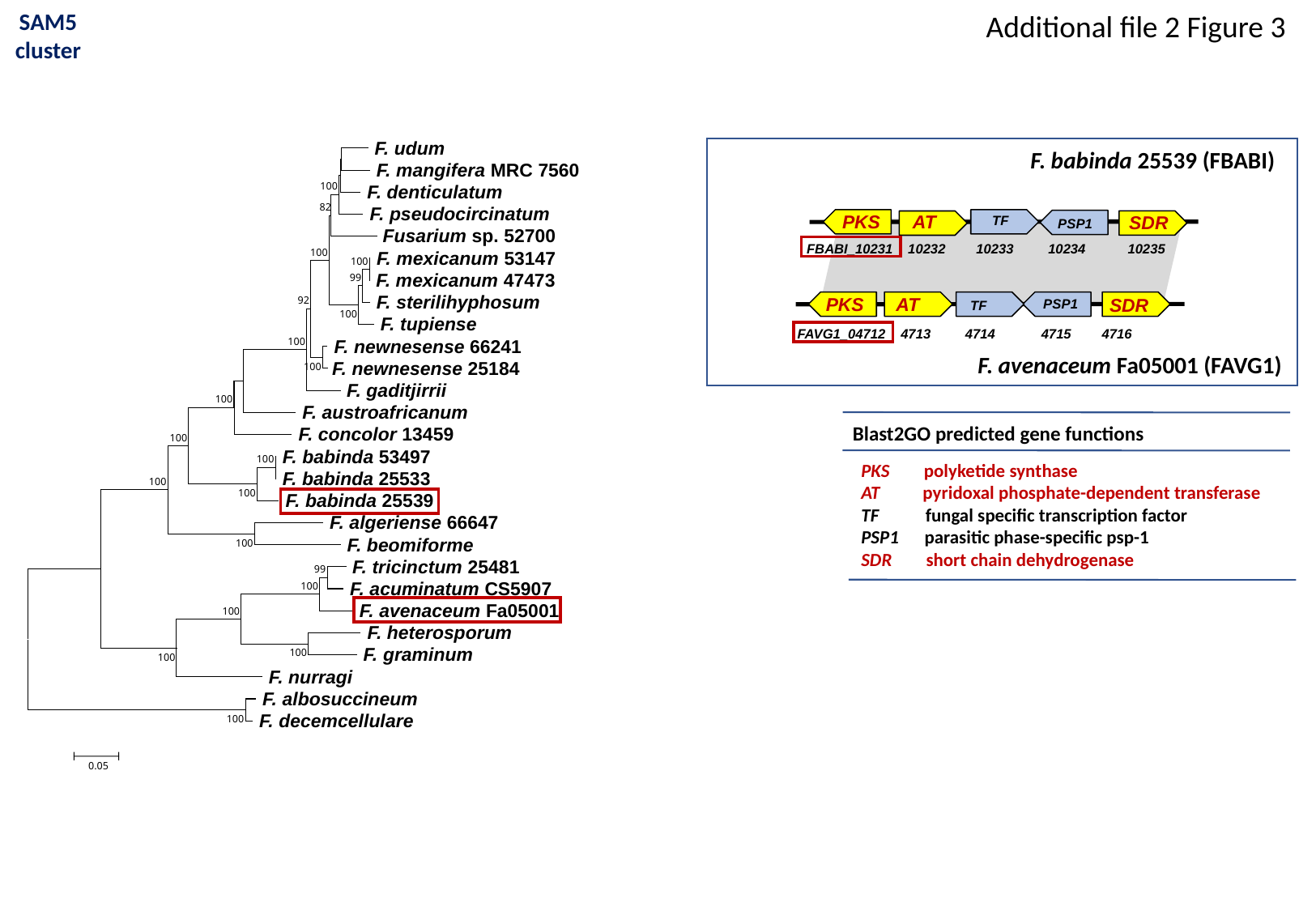

SAM5 cluster
Additional file 2 Figure 3
 F. udum
100
82
100
100
99
92
100
100
100
100
100
100
100
100
100
99
100
100
100
100
100
0.05
 F. mangifera MRC 7560
 F. denticulatum
 F. pseudocircinatum
 Fusarium sp. 52700
 F. mexicanum 53147
 F. mexicanum 47473
 F. sterilihyphosum
 F. tupiense
 F. newnesense 66241
F. newnesense 25184
 F. gaditjirrii
 F. austroafricanum
 F. concolor 13459
 F. babinda 53497
 F. babinda 25533
 F. babinda 25539
 F. algeriense 66647
 F. beomiforme
 F. tricinctum 25481
 F. acuminatum CS5907
 F. avenaceum Fa05001
 F. heterosporum
 F. graminum
 F. nurragi
 F. albosuccineum
 F. decemcellulare
F. babinda 25539 (FBABI)
PKS
AT
SDR
TF
PSP1
FBABI_10231 10232 10233 10234 10235
PKS
AT
SDR
PSP1
TF
FAVG1_04712 4713 4714 4715 4716
F. avenaceum Fa05001 (FAVG1)
Blast2GO predicted gene functions
PKS polyketide synthase
AT pyridoxal phosphate-dependent transferase
TF fungal specific transcription factor
PSP1 parasitic phase-specific psp-1
SDR short chain dehydrogenase

## Slide 4
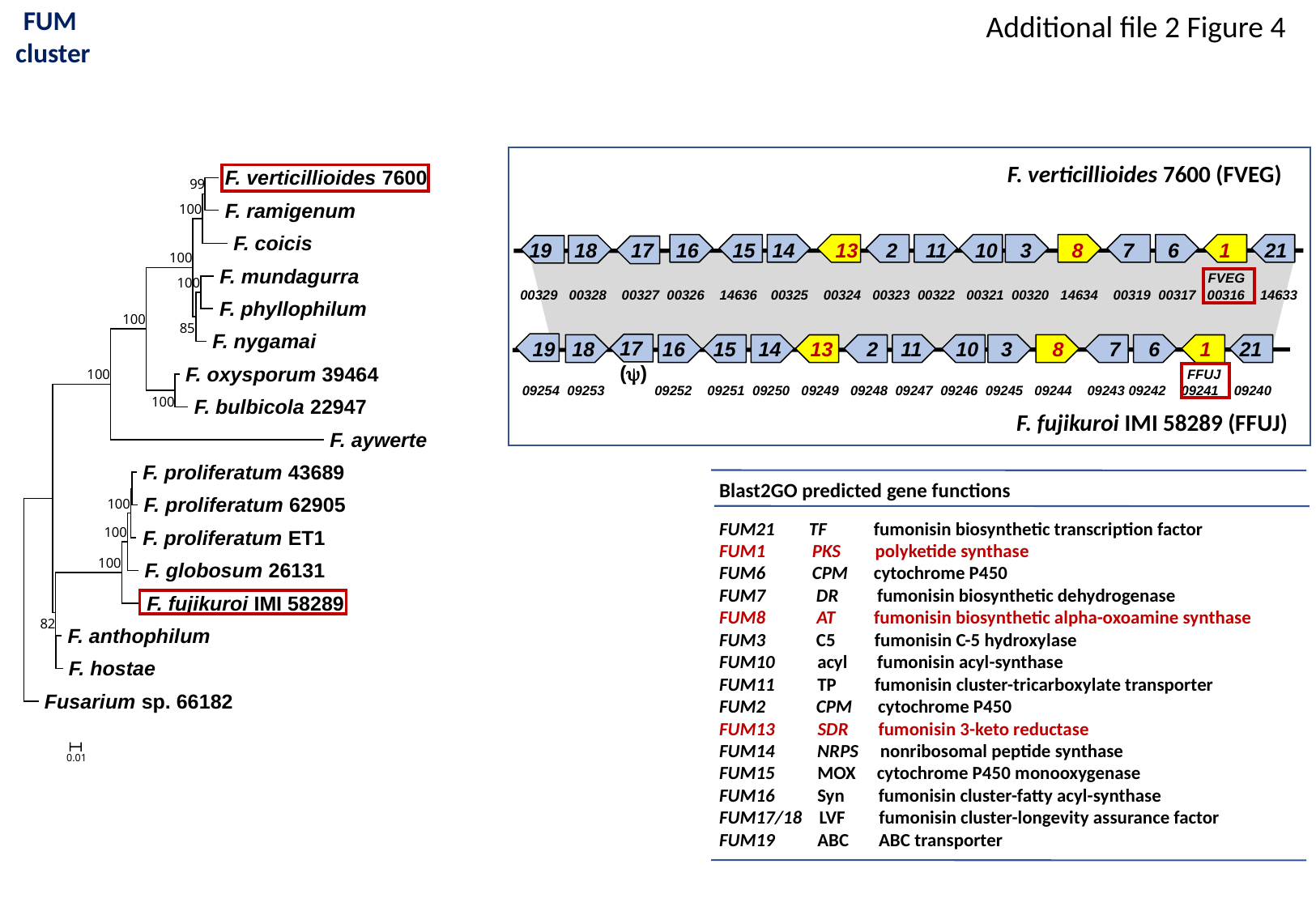

FUM
 cluster
Additional file 2 Figure 4
 F. verticillioides 7600
99
 F. ramigenum
100
 F. coicis
100
 F. mundagurra
100
 F. phyllophilum
100
85
 F. nygamai
 F. oxysporum 39464
100
100
 F. bulbicola 22947
 F. aywerte
 F. proliferatum 43689
 F. proliferatum 62905
100
100
 F. proliferatum ET1
100
 F. globosum 26131
 F. fujikuroi IMI 58289
82
 F. anthophilum
 F. hostae
 Fusarium sp. 66182
0.01
F. verticillioides 7600 (FVEG)
19 18 17 16 15 14 13 2 11 10 3 8 7 6 1 21
FVEG
00329 00328 00327 00326 14636	 00325 00324 00323 00322 00321 00320 14634 00319 00317 00316 14633
17
()
19 18 16 15 14 13 2 11 10 3 8 7 6 1 21
FFUJ
09254 09253 09252 09251 09250 09249 09248 09247 09246 09245 09244 09243 09242 09241 09240
F. fujikuroi IMI 58289 (FFUJ)
Blast2GO predicted gene functions
FUM21 TF fumonisin biosynthetic transcription factor
FUM1 PKS polyketide synthase
FUM6 CPM cytochrome P450
FUM7 DR fumonisin biosynthetic dehydrogenase
FUM8 AT fumonisin biosynthetic alpha-oxoamine synthase
FUM3 C5	 fumonisin C-5 hydroxylase
FUM10 acyl fumonisin acyl-synthase
FUM11 TP fumonisin cluster-tricarboxylate transporter
FUM2 CPM cytochrome P450
FUM13 SDR fumonisin 3-keto reductase
FUM14 NRPS nonribosomal peptide synthase
FUM15 MOX cytochrome P450 monooxygenase
FUM16 Syn fumonisin cluster-fatty acyl-synthase
FUM17/18 LVF fumonisin cluster-longevity assurance factor
FUM19 ABC ABC transporter
