# Supplementary material for: Identification and distribution of gene clusters required for synthesis of sphingolipid metabolism inhibitors in diverse species of the filamentous fungus Fusarium
Source: BMC Genomics. 2020 Jul 23;21:510. doi: 10.1186/s12864-020-06896-1 (PMC7376913; doi:10.1186/s12864-020-06896-1)
Supplement: Supplementary file 4 — Additional file 4. Distribution of SAM clusters in 87 representative Fusarium genome sequences. [file 12864_2020_6896_MOESM4_ESM.docx]

**Additional file 4.** Distribution of SAM clusters in 87 representative *Fusarium* genome sequences.

|  |  |  | **Distribution^a^** | | | | | | |  | | | | |
| --- | --- | --- | --- | --- | --- | --- | --- | --- | --- | --- | --- | --- | --- | --- |
| **Species Complex** | **Species** | **Strain^b^** | **FUM** | **SAM1** | **SAM2** | **SAM3** | **SAM4** | **SAM5** | **BioProject** | | **GenBank Genome Sequence Accession^c^** | **N50** | **No. Contigs** | **Genome Size (Mb)** |
| Sambucinum | *F. austroamericanum* | NRRL 02903 |  |  |  |  |  |  | PRJNA436741 | | JAAMOD000000000* | 108,815 | 900 | 36.9 |
| Sambucinum | *F. brasilicum* | NRRL 31281 |  |  |  |  |  |  | PRJNA436741 | | JABCJS000000000* | 145,992 | 569 | 36.8 |
| Sambucinum | *F. cortaderiae* | NRRL 29297 |  |  |  |  |  |  | PRJNA436741 | | JABCJT000000000* | 172,928 | 546 | 36.7 |
| Sambucinum | *F. kyushuense* | NRRL 25348 |  |  |  | **•** |  |  | PRJNA436741 | | JABCJU000000000* | 251,145 | 325 | 36.0 |
| Chlamydosporum | *F. aywerte* | NRRL 25410 |  |  |  |  |  |  | PRJNA596283 | | JABCQV000000000* | 111,181 | 911 | 36.0 |
| Incarnatum-equiseti | *F. luffae* (FIESC 18) | NRRL 66473 |  |  |  | **•** |  |  | PRJNA436969 | | JABCJV000000000* | 80,658 | 1049 | 37.4 |
| Incarnatum-equiseti | FIESC 24 | NRRL 66471 |  |  |  | **•** |  |  | PRJNA436969 | | JAALXH000000000* | 85,566 | 1049 | 37.8 |
| Tricinctum | *F. acuminatum* | CS5907 |  |  |  |  |  |  | PRJEB1737 | | CBMG000000000 | 30684 | 3907 | 44.0 |
| Tricinctum | *F. acuminatum* | F829 |  |  |  |  |  |  | PRJNA596296 | | JABEEU000000000* | 38,483 | 4443 | 46.9 |
| Tricinctum | *F. tricinctum* | NRRL 25481 |  |  |  |  |  |  | PRJNA596296 | | JAALXJ000000000* | 87,772 | 1033 | 41.0 |
| Tricinctum | *F. avenaceum* | NRRL 54939 = Fa05001 |  |  |  |  |  |  | PRJNA253730 | | JPYM00000000 | 1,436,644 | 99 | 41.6 |
| Heterosporum | *F. graminum* | NRRL 20692 |  |  |  |  |  |  | PRJNA596285 | | JAAGWP000000000* | 86,148 | 977 | 35.0 |
| Heterosporum | *F. heterosporum* | NRRL 20693 |  |  |  |  |  |  | PRJNA596285 | | JAAGWQ000000000* | 175,877 | 793 | 35.7 |
| Not assigned | *F. nurragi* | NRRL 36452 |  |  |  |  |  |  | PRJNA596294 | | JAALXI000000000* | 114,524 | 854 | 35.5 |
| Fujikuroi – African | *F. coicis* | NRRL 66233 |  |  |  |  |  |  | PRJNA565749 | | JAAOAJ000000000* | 83,421 | 1267 | 42.7 |
| Fujikuroi – African | *F. verticillioides* | FGSC 7600 |  |  |  |  |  |  | PRJNA15553 | | AAIM00000000 | 4,246,266 | 24 | 41.9 |
| Fujikuroi – African | *F. napiforme* | NRRL 25196 |  |  |  |  |  |  | PRJNA565749 | | JAAOAO000000000* | 78,351 | 1411 | 42.0 |
| Fujikuroi – African | *F. tjaetaba* | NRRL 66243 |  |  |  |  |  |  | PRJNA565749 | | JAAQRI000000000* | 151,678 | 867 | 43.1 |
| Fujikuroi – African | *F. ramigenum* | NRRL 25208 |  |  |  |  |  |  | PRJNA565749 | | JAAOAT000000000* | 87,353 | 1578 | 46.5 |
| Fujikuroi – African | *F. brevicatenulatum* | NRRL 25447 |  |  |  |  |  |  | PRJNA565749 | | JABEEJ000000000* | 61,030 | 1.764 | 42.5 |
| Fujikuroi – African | *F. pseudoanthophilum* | NRRL 25211 |  |  |  |  |  |  | PRJNA565749 | | JAAOAR000000000* | 62,432 | 1816 | 42.8 |
| Fujikuroi – African | *F. pseudonygamai* | NRRL 13592 |  |  |  |  |  |  | PRJNA565749 | | JAAQRH000000000* | 50,630 | 2619 | 42.2 |
| Fujikuroi – African | *F. thapsinum* | NRRL 22049 |  |  |  |  |  |  | PRJNA565749 | | JAAOAX000000000* | 324,635 | 446 | 40.9 |
| Fujikuroi – African | *F. denticulatum* | NRRL 25311 |  |  |  |  |  |  | PRJNA565749 | | JAAOAK000000000* | 109,226 | 909 | 43.2 |
| Fujikuroi – African | *F. pseudocircinatum* | NRRL 36939 |  |  |  |  |  |  | PRJNA565749 | | JAAOAS000000000* | 118,013 | 1041 | 43.3 |
| Fujikuroi – African | *F. nygamai* | NRRL 66327 |  |  |  |  |  |  | PRJNA565749 | | JAAOAP000000000* | 59,475 | 2802 | 48.5 |
| Fujikuroi – African | *F. mundagurra* | NRRL 66235 |  |  |  |  |  |  | PRJNA565749 | | JAAOAN000000000* | 113,243 | 1616 | 49.1 |
| Fujikuroi – African | *F. phyllophilum* | NRRL 13617 |  |  |  |  |  |  | PRJNA565749 | | JAAOAQ000000000* | 87,347 | 1628 | 43.4 |
| Fujikuroi – African | *F. udum* | NRRL 25194 |  |  |  |  |  |  | PRJNA565749 | | JAAQPG000000000* | 43,406 | 2354 | 44.6 |
| Fujikuroi – African | *F. secorum* | NRRL 62593 |  |  |  |  |  |  | PRJNA565749 | | JABEEM000000000* | 16,830 | 8297 | 49.6 |
| Fujikuroi – African | *F. acutatum* | NRRL 13308 |  |  |  |  |  |  | PRJNA565749 | | JAADJF000000000* | 116,016 | 983 | 43.3 |
| Fujikuroi – American | *F. bulbicola* | NRRL 22947 |  |  |  |  |  |  | PRJNA565749 | | JAAOAH000000000* | 29,796 | 3116 | 42.3 |
| Fujikuroi – American | *F. bulbicola* | NRRL 25176 |  |  |  |  |  |  | PRJNA565749 | | JAAOAI000000000* | 55,043 | 1736 | 43.6 |
| Fujikuroi – American | *F. circinatum* | NRRL 25331 |  |  |  |  |  |  | PRJNA565749 | | JAAQPE000000000* | 96,195 | 1223 | 42.6 |
| Fujikuroi – American | *F. succisae* | NRRL 13298 |  |  |  |  |  |  | PRJNA565749 | | JAAOAW000000000* | 329,061 | 538 | 45.4 |
| Fujikuroi – American | *F. anthophilum* | NRRL 25214 |  |  |  |  |  |  | PRJNA565749 | | JABEVY000000000* | 124,798 | 1118 | 45.7 |
| Fujikuroi – American | *F. subglutinans* | NRRL 66333 |  |  |  |  |  |  | PRJNA565749 | | JAAOAV000000000* | 181,117 | 905 | 44.2 |
| Fujikuroi – American | *F. begonia* | NRRL 25300 |  |  |  |  |  |  | PRJNA565749 | | JAAOAG000000000* | 98,470 | 1002 | 43.9 |
| Fujikuroi – American | *F. guttiforme* | NRRL 53293 |  |  |  |  |  |  | PRJNA565749 | | JABSTN000000000* | 80,435 | 1385 | 43.3 |
| Fujikuroi – American | *F. mexicanum* | NRRL 53147 |  |  |  |  |  |  | PRJNA565749 | | JAAOAM000000000* | 146,662 | 958 | 44.0 |
| Fujikuroi – American | *F. tupiense* | NRRL 53984 |  |  |  |  |  |  | PRJNA565749 | | JABEEO000000000* | 88,403 | 1665 | 45.5 |
| Fujikuroi – American | *F. sterilihyphosum* | NRRL 25623 |  |  |  |  |  |  | PRJNA565749 | | JAAOAU000000000* | 74,938 | 2178 | 46.9 |
| Fujikuroi – American | *F. agapanthi* | NRRL 31653 |  |  |  |  |  |  | PRJNA565749 | | LUFC00000000 | 43,289 | 2350 | 41.3 |
| Fujikuroi – basal lineage | *Fusarium* sp. | NRRL 52700 |  |  |  |  |  |  | PRJNA565749 | | JAAQRM000000000* | 417243 | 528 | 43.0 |
| Fujikuroi – Asian | *F. proliferatum* | ET1 |  |  |  |  |  |  | PRJNA576857 | | FJOF00000000 | 782,754 | 221 | 45.2 |
| Fujikuroi – Asian | *F. globosum* | NRRL 26131 |  |  |  |  |  |  | PRJNA565749 | | JAAQPF000000000* | 71,375 | 1696 | 44.6 |
| Fujikuroi – Asian | *F. Fujikuroi* | IMI58289 |  |  |  |  |  |  | PRJNA322155 | | GCF_900079805.1 | 1,182,607 | 65 | 43.8 |
| Fujikuroi – Asian | *Fusarium* sp. | NRRL 25303 |  |  |  |  |  |  | PRJNA565749 | | JAAOAY000000000* | 102,206 | 941 | 43.7 |
| Fujikuroi – Asian | *F. mangiferae* | MRC 7560 |  |  |  |  |  |  | PRJEB9887 | | FCQH00000000 | 100,034 | 2060 | 48.6 |
| Fujikuroi – basal lineage | *F. dlaminii* | NRRL 13164 |  |  |  |  |  |  | PRJNA565749 | | JAAOAL000000000* | 478,666 | 484 | 44.1 |
| Nisikadoi | *F. gaditjirrii* | NRRL 45417 |  |  |  |  |  |  | PRJNA596290 | | JABFAI000000000* | 134,166 | 835 | 41.9 |
| Oxysporum | *F. oxysporum* | NRRL 39464 |  |  |  |  |  |  | PRJNA596293 | | JAAFOW000000000* | 18,508 | 5286 | 47.0 |
| Newnesense | *F. newnesense* | NRRL 66241 |  |  |  |  |  |  | PRJNA416189 | | JABCJW000000000* | 23,488 | 5464 | 48.7 |
| Redolens | *F. hostae* | NRRL 29888 |  |  |  |  |  |  | PRJNA485357 | | JABCJX000000000* | 54,891 | 3090 | 46.0 |
| Burgessii | *F. algeriense* | NRRL 66647 |  |  |  |  |  |  | PRJNA416189 | | PVPZ00000000 | 42,571 | 3535 | 48.9 |
| Burgessii | *F. burgessii* | NRRL 66654 |  |  |  |  |  |  | PRJNA416189 | | PVQA00000000 | 58,099 | 3437 | 49.3 |
| Burgessii | *F. beomiforme* | NRRL 25174 |  |  |  |  |  |  | PRJNA416189 | | PVQB00000000 | 77,355 | 1869 | 46.5 |
| Concolor | *F. austroafricanum* | NRRL 53441 |  |  |  |  |  |  | PRJNA485357 | | JAADJG000000000* | 56,596 | 2869 | 46.2 |
| Concolor | *F. concolor* | NRRL 13459 |  |  |  |  |  |  | PRJNA485357 | | JABCJY000000000* | 54,123 | 3182 | 49.6 |
| Concolor | *F. anguoides* | NRRL 25385 |  |  |  | **•** |  |  | PRJNA485357 | | JAALXK000000000* | 110,646 | 744 | 38.9 |
| Babinda | *F. babinda* | NRRL 53497 |  |  |  |  |  |  | PRJNA485357 | | JABCJZ000000000* | 145,770 | 883 | 44.2 |
| Babinda | *F. babinda* | NRRL 25533 |  |  |  |  |  |  | PRJNA485357 | | JAALXL000000000* | 110,403 | 1090 | 43.8 |
| Torreyae | *F. continuum* | NRRL 66286 |  |  |  |  |  |  | PRJNA488083 | | JABCKB000000000* | 63,250 | 1748 | 38.3 |
| Lateritium | *F. sarcochroum* | NRRL 20472 |  |  |  | **•** |  |  | PRJNA596286 | | JABEXW000000000* | 55,061 | 1849 | 46.5 |
| buharicum | *Fusarium* sp. | NRRL 66182 |  |  |  | **•** |  |  | PRJNA596286 | | JABFAK000000000* | 64,063 | 1110 | 33.3 |
| Staphyleae | *F. zealandicum* | NRRL 22465 |  |  |  | **•** |  |  | PRJNA596292 | | JABEYC000000000* | 43,137 | 1836 | 33.5 |
| Decemcellulare | *F. albosuccineum* | NRRL 20459 |  |  |  |  |  |  | PRJNA596284 | | JAADYS000000000* | 23,353 | 4197 | 50.9 |
| Decemcellulare | *F. decemcellulare* | NRRL 13412 |  |  |  |  |  |  | PRJNA596284 | | JAAGWO000000000* | 35,459 | 3482 | 53.7 |
| Solani | *F. oligoseptarum* | NRRL 62579 |  |  |  |  |  |  | PRJNA389173 | | NKCK00000000 | 55867 | 3145 | 48.8 |
| Solani | *Fusarium sp.* [AF-12] | NRRL 62957 |  |  |  |  |  |  | PRJNA511036 | | JAALXM000000000* | 3,722 | 18524 | 43.8 |
| Solani | *F. euwallaceae* [AF-2] | NRRL 62626 = UCR1854 |  |  |  |  |  |  | PRJNA341909 | | MIKF00000000 | 64,414 | 3437 | 50.3 |
| Solani | *F. ambrosium* [AF-1] | NRRL 20438 |  |  |  |  |  |  | PRJNA389173 | | NIZV00000000 | 39,278 | 3919 | 49.3 |
| Solani | *Fusarium sp.* [AF-11] | NRRL 62944 |  |  |  |  |  |  | PRJNA511036 | | JABCKC000000000* | 49,850 | 2972 | 49.2 |
| Solani | *F. tuaranense* | NRRL 46518 |  |  |  |  |  |  | PRJNA511036 | | JABEEN000000000* | 32646 | 3981 | 48.9 |
| Solani | *Fusarium* sp. [AF-10] | NRRL 62941 |  |  |  |  |  |  | PRJNA511036 | | JAALXN000000000* | 28,117 | 4545 | 48.1 |
| Solani | *F. kuroshium* | UCR3641 |  |  |  |  |  |  | PRJNA389173 | | SRR5712560.1 | 91,081 | 1414 | 46.6 |
| Solani | *Fusarium* sp. [AF-6] | NRRL 62590 |  |  |  |  |  |  | PRJNA389173 | | NKCJ00000000 | 35,733 | 3394 | 44.0 |
| Solani | *Fusarium* sp*.* [AF-7] | NRRL 62610 |  |  |  |  |  |  | PRJNA511036 | | JABCKE000000000* | 6971 | 12070 | 46.1 |
| Solani | *Fusarium* sp. [AF-8] | NRRL 62584 |  |  |  |  |  |  | PRJNA389173 | | NKCI00000000 | 59,384 | 2582 | 47.6 |
| Solani | *Fusarium* sp. [AF-9] | NRRL 66088 |  |  |  |  |  |  | PRJNA511036 | | JABCKD000000000* | 46,149 | 4445 | 48.7 |
| Solani | *F. falciforme* | NRRL 43529 |  |  |  |  |  |  | PRJNA511036 | | JABEEK000000000* | 33,039 | 3427 | 48.2 |
| Solani | *Fusarium* sp. | NRRL 22101 |  |  |  |  |  |  | PRJNA511036 | | JABELF000000000* | 29,270 | 4875 | 65.6 |
| Solani | *F. solani f.* sp. *pisi* | 77-13-4 |  |  |  |  |  |  | PRJNA16586 | | ACJF00000000 | 1,273,910 | 209 | 51.3 |
| Solani | *F. neocosmosporiellum* | NRRL 22166 |  |  |  |  |  |  | PRJNA511036 | | SSHR00000000 | 29,423 | 5509 | 54.1 |
| Solani | *F. phaseoli* | NRRL 22396 |  |  |  |  |  |  | PRJNA511036 | | JABEEL000000000* | 91,209 | 1484 | 46.1 |
| solnai | *Fusarium* sp. | NRRL 22178 |  |  |  |  |  |  | PRJNA511036 | | JAANQP000000000* | 76,035 | 1682 | 45.5 |
| Solani | *F. virguliforme* | NRRL 31041 |  |  |  |  |  |  | PRJNA511036 | | JABEEP000000000* | 30,734 | 3854 | 45.4 |

**^a^** A gray cell indicates that a cluster is present in a given strain/species, whereas a white cell indicates that a cluster is absent. In the SAM3 column, a red dot indicates that the cluster does not include a short-chain dehydrogenase reductase (SDR) gene.

**^b^** Strain designations that include the NRRL prefix were from the ARS (NRRL) Culture Collection at the US Department of Agriculture in Peoria, Illinois. Origins of other strains were as follows: 77-13-4 – Laboratory of Hans VanEtten, University of Arizona; CS5907 – Commonwealth Scientific and Industrial Research Organisation (CSIRO) Plant Industry, Brisbane, Australia; ET1 - Laboratory of Bettina Tudzynski, University of Münster, Germany; F829 – Laboratory of Todd J. Ward, USDA ARS NCAUR; Fa05001 Laboratory of Erik Lysoe, Norwegian Institute of Agricultural and Environmental Research, Ås, Norway; FGSC 7600 – Fungal Genetics Stock Center, Kansas State University; IMI58289 – Culture Collection of the Centre for Agriculture and Bioscience International (CABI), United Kingdom (through laboratory of Bettina Tudzynski, University of Münster, Germany); MRC 7560 – South African Medical Research Council, Programme on Mycotoxins and Experimental Carcinogenesis (now at Institute of Biomedical and Microbial Biotechnology, Cape Peninsula University of Technology, South Africa); and UCR3641 – Laboratory of Jason Stajich, University of California-Riverside. [AF-] designations are Ambrosia *Fusarium* Clade designations as previously described [1].

**^c^** An asterisk (*) next to accession numbers indicates genome sequence data that were generated and submitted to the GenBank/NCBI database as part of the current study.

**Reference**

1. Aoki T, Smith JA, Kasson MT, Freeman S, Geiser DM, Geering ADW, O'Donnell K: **Three novel Ambrosia *Fusarium* Clade species producing clavate macroconidia known (*F. floridanum* and *F. obliquiseptatum*) or predicted (*F. tuaranense*) to be farmed by *Euwallacea* spp. (Coleoptera: Scolytinae) on woody hosts**. *Mycologia* 2019, **111**(6):919-935.
