# Supplementary material for: Identification and distribution of gene clusters required for synthesis of sphingolipid metabolism inhibitors in diverse species of the filamentous fungus Fusarium
Source: BMC Genomics. 2020 Jul 23;21:510. doi: 10.1186/s12864-020-06896-1 (PMC7376913; doi:10.1186/s12864-020-06896-1)
Supplement: Supplementary file 5 — Additional file 5. Phylogenetic tree from NOTUNG reconciliation analysis inferred horizontal transfer events for each SAM cluster, SAM1 (Additional file 5 Figure 1), SAM2 (Additional file 5 Figure 2), SAM3 (Additional file 5 Figure 3), SAM4 (Additional file 5 Figure 4), and SAM5 (Additional file 5 Figure 5). In the NOTUNG analysis, the species tree was inferred from concatenated alignments of the coding region sequences of 13 housekeeping genes from 96 representative Fusarium strains (Additional file 5 Figure 6). [file 12864_2020_6896_MOESM5_ESM.pptx]

## Slide 1
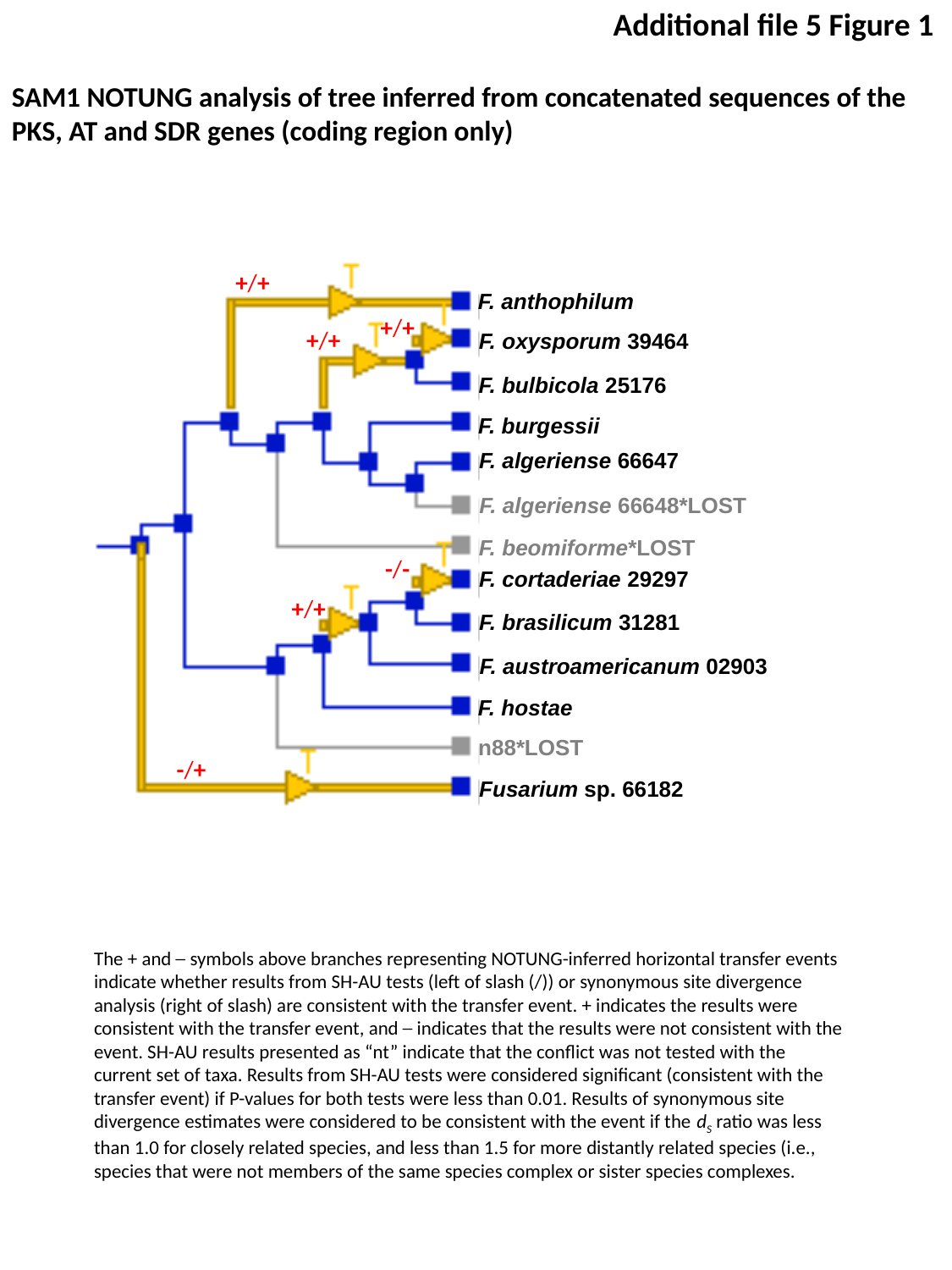

Additional file 5 Figure 1
SAM1 NOTUNG analysis of tree inferred from concatenated sequences of the PKS, AT and SDR genes (coding region only)
+/+
+/+
+/+
-/-
+/+
-/+
 F. anthophilum
 F. oxysporum 39464
 F. bulbicola 25176
 F. burgessii
 F. algeriense 66647
 F. algeriense 66648*LOST
 F. beomiforme*LOST
 F. cortaderiae 29297
 F. brasilicum 31281
 F. austroamericanum 02903
 F. hostae
 n88*LOST
 Fusarium sp. 66182
The + and ─ symbols above branches representing NOTUNG-inferred horizontal transfer events indicate whether results from SH-AU tests (left of slash (/)) or synonymous site divergence analysis (right of slash) are consistent with the transfer event. + indicates the results were consistent with the transfer event, and ─ indicates that the results were not consistent with the event. SH-AU results presented as “nt” indicate that the conflict was not tested with the current set of taxa. Results from SH-AU tests were considered significant (consistent with the transfer event) if P-values for both tests were less than 0.01. Results of synonymous site divergence estimates were considered to be consistent with the event if the dS ratio was less than 1.0 for closely related species, and less than 1.5 for more distantly related species (i.e., species that were not members of the same species complex or sister species complexes.

## Slide 2
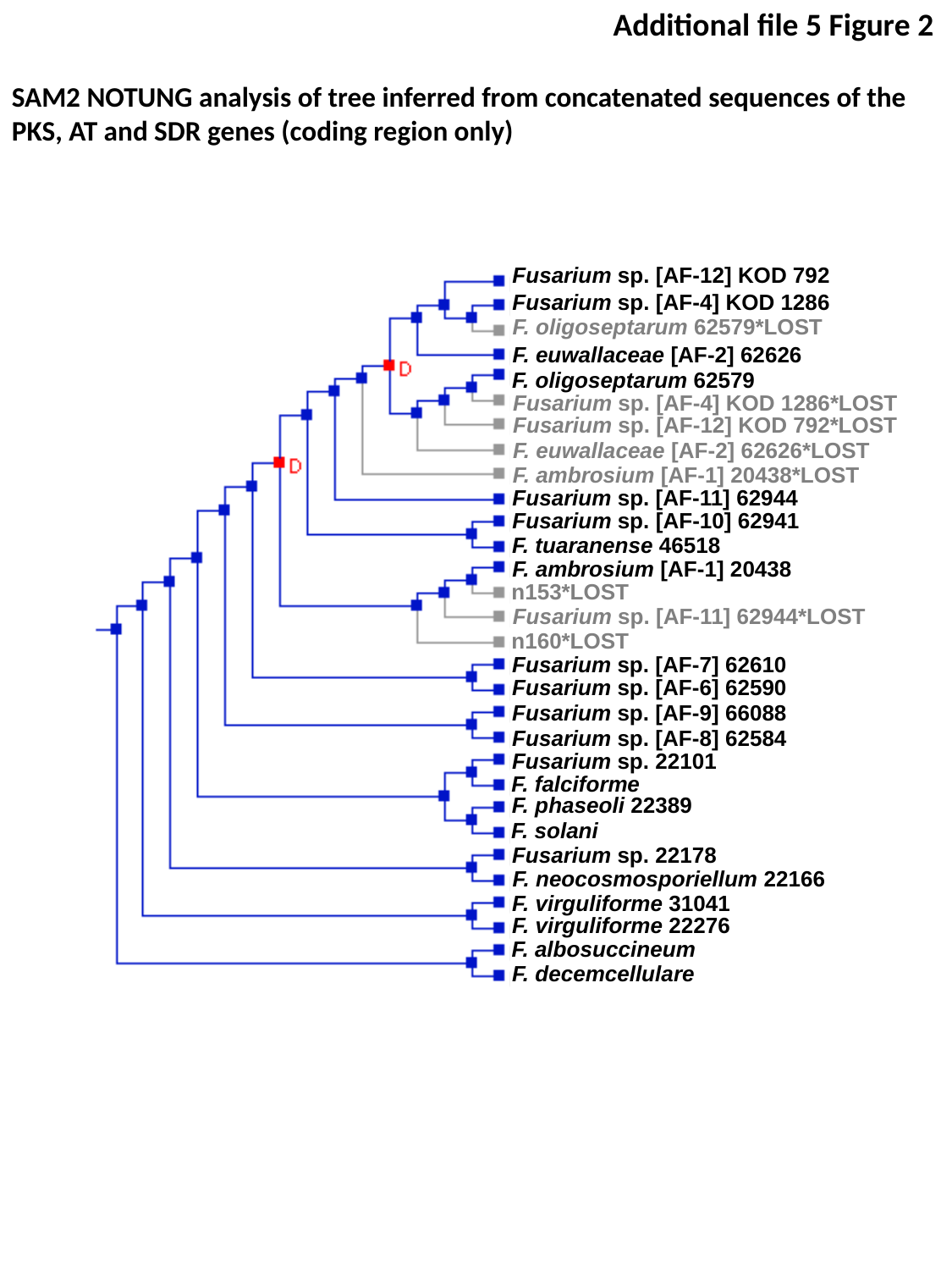

Additional file 5 Figure 2
SAM2 NOTUNG analysis of tree inferred from concatenated sequences of the PKS, AT and SDR genes (coding region only)
 Fusarium sp. [AF-12] KOD 792
 Fusarium sp. [AF-4] KOD 1286
 F. oligoseptarum 62579*LOST
 F. euwallaceae [AF-2] 62626
 F. oligoseptarum 62579
 Fusarium sp. [AF-4] KOD 1286*LOST
 Fusarium sp. [AF-12] KOD 792*LOST
 F. euwallaceae [AF-2] 62626*LOST
 F. ambrosium [AF-1] 20438*LOST
 Fusarium sp. [AF-11] 62944
 Fusarium sp. [AF-10] 62941
 F. tuaranense 46518
 F. ambrosium [AF-1] 20438
 n153*LOST
 Fusarium sp. [AF-11] 62944*LOST
 n160*LOST
 Fusarium sp. [AF-7] 62610
 Fusarium sp. [AF-6] 62590
 Fusarium sp. [AF-9] 66088
 Fusarium sp. [AF-8] 62584
 Fusarium sp. 22101
 F. falciforme
 F. phaseoli 22389
 F. solani
 Fusarium sp. 22178
 F. neocosmosporiellum 22166
 F. virguliforme 31041
 F. virguliforme 22276
 F. albosuccineum
 F. decemcellulare

## Slide 3
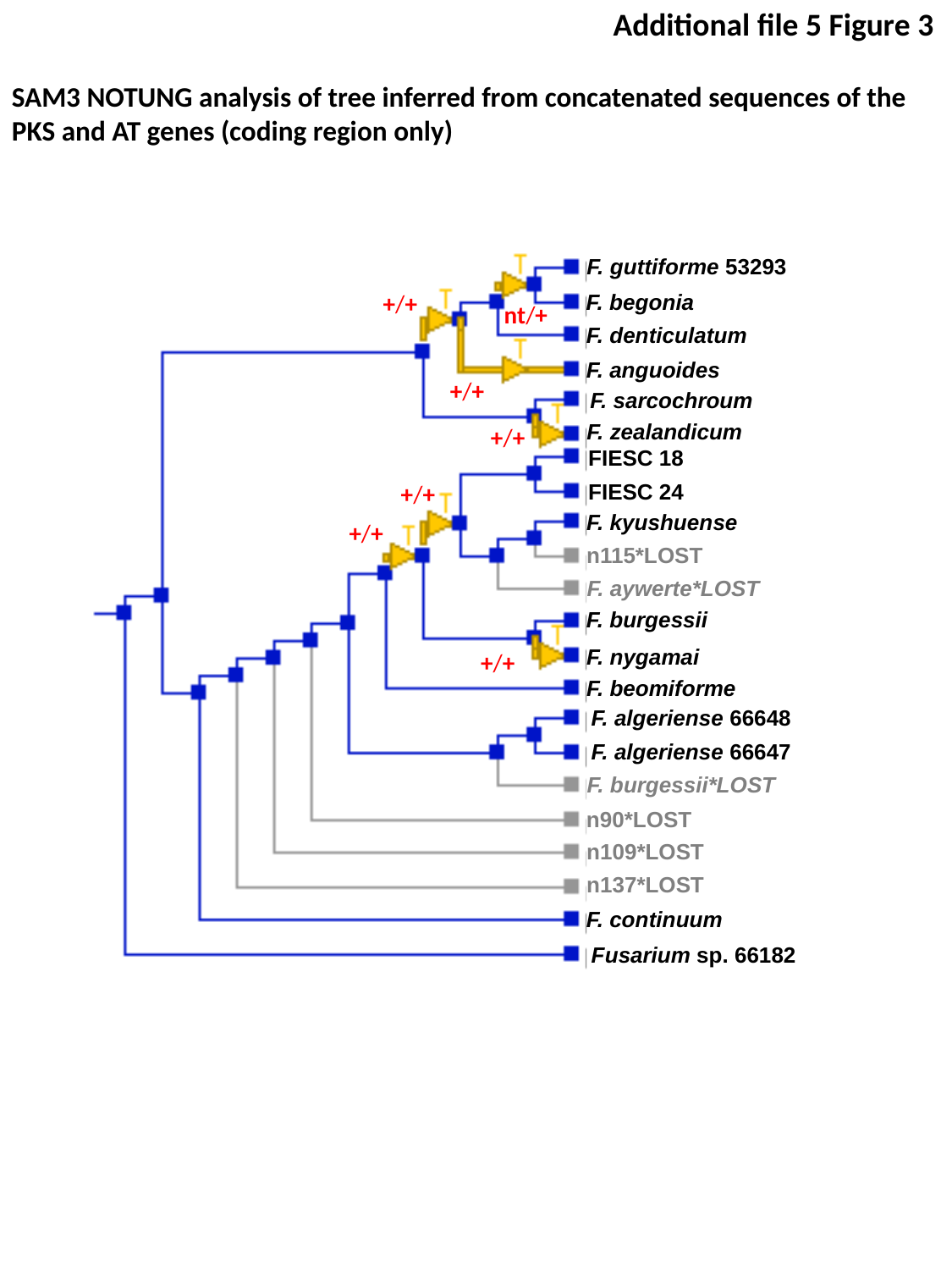

Additional file 5 Figure 3
SAM3 NOTUNG analysis of tree inferred from concatenated sequences of the PKS and AT genes (coding region only)
+/+
nt/+
+/+
+/+
+/+
+/+
+/+
 F. guttiforme 53293
 F. begonia
 F. denticulatum
 F. anguoides
 F. sarcochroum
 F. zealandicum
 FIESC 18
 FIESC 24
 F. kyushuense
 n115*LOST
 F. aywerte*LOST
 F. burgessii
 F. nygamai
 F. beomiforme
 F. algeriense 66648
 F. algeriense 66647
 F. burgessii*LOST
 n90*LOST
 n109*LOST
 n137*LOST
 F. continuum
 Fusarium sp. 66182

## Slide 4
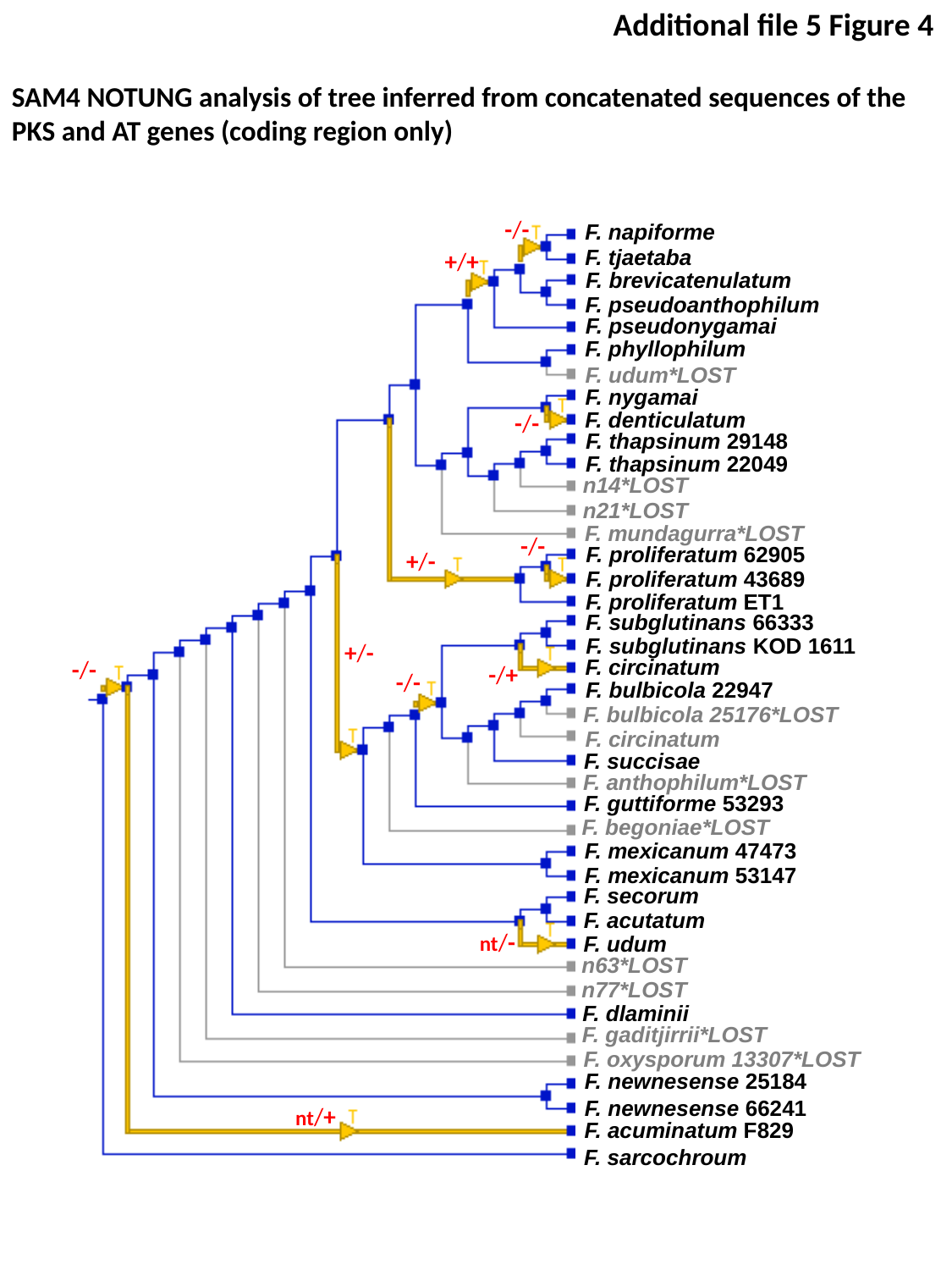

Additional file 5 Figure 4
SAM4 NOTUNG analysis of tree inferred from concatenated sequences of the PKS and AT genes (coding region only)
-/-
+/+
-/-
-/-
+/-
+/-
-/-
-/+
-/-
nt/-
nt/+
 F. napiforme
 F. tjaetaba
 F. brevicatenulatum
 F. pseudoanthophilum
 F. pseudonygamai
 F. phyllophilum
 F. udum*LOST
 F. nygamai
 F. denticulatum
 F. thapsinum 29148
 F. thapsinum 22049
n14*LOST
n21*LOST
F. mundagurra*LOST
 F. proliferatum 62905
 F. proliferatum 43689
 F. proliferatum ET1
 F. subglutinans 66333
 F. subglutinans KOD 1611
 F. circinatum
 F. bulbicola 22947
 F. bulbicola 25176*LOST
 F. circinatum
 F. succisae
F. anthophilum*LOST
 F. guttiforme 53293
F. begoniae*LOST
 F. mexicanum 47473
 F. mexicanum 53147
 F. secorum
 F. acutatum
 F. udum
n63*LOST
n77*LOST
 F. dlaminii
F. gaditjirrii*LOST
 F. oxysporum 13307*LOST
 F. newnesense 25184
 F. newnesense 66241
 F. acuminatum F829
 F. sarcochroum

## Slide 5
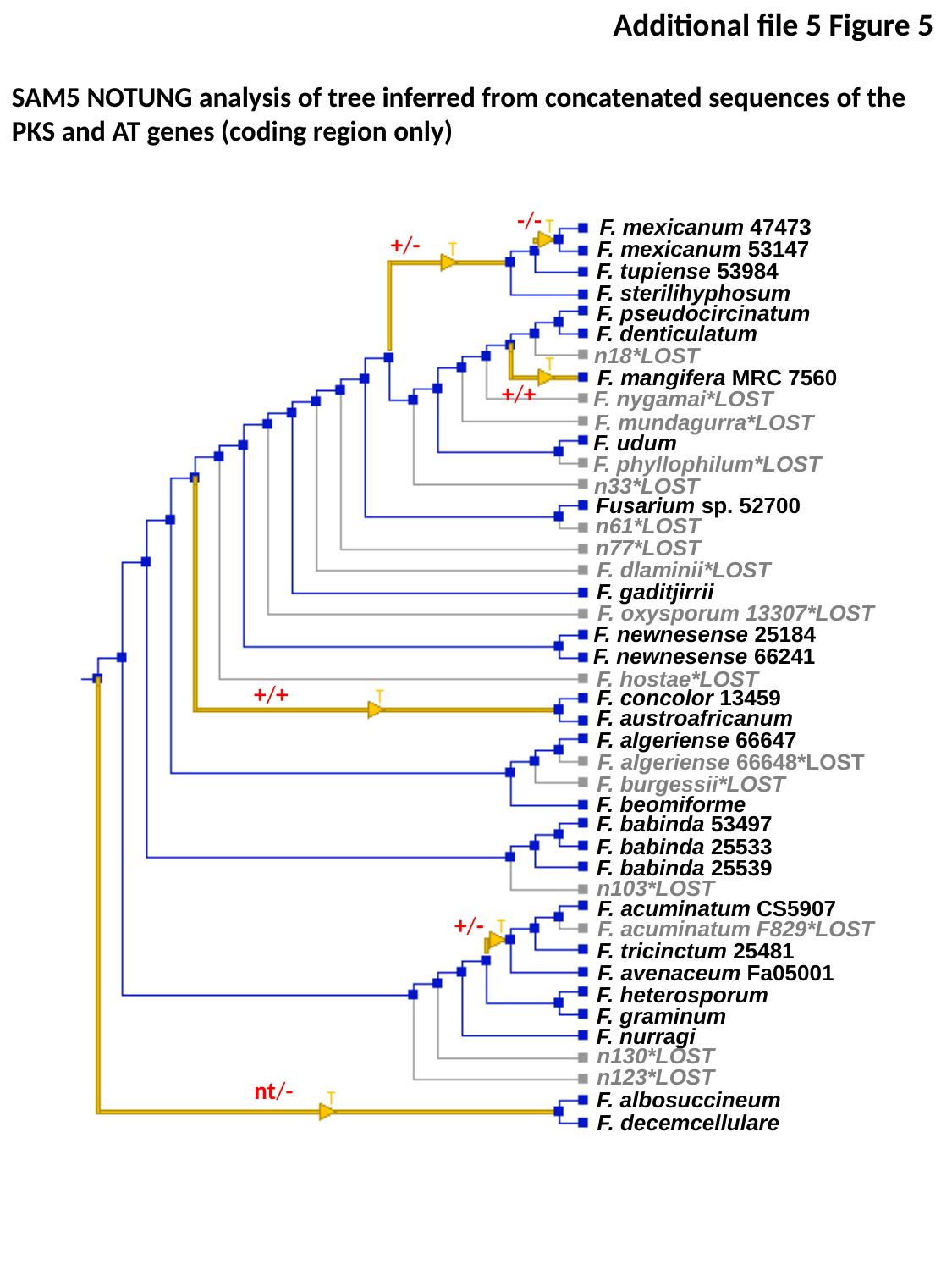

Additional file 5 Figure 5
SAM5 NOTUNG analysis of tree inferred from concatenated sequences of the PKS and AT genes (coding region only)
-/-
 F. mexicanum 47473
+/-
 F. mexicanum 53147
 F. tupiense 53984
 F. sterilihyphosum
 F. pseudocircinatum
 F. denticulatum
n18*LOST
 F. mangifera MRC 7560
+/+
F. nygamai*LOST
F. mundagurra*LOST
 F. udum
F. phyllophilum*LOST
n33*LOST
 Fusarium sp. 52700
n61*LOST
n77*LOST
 F. dlaminii*LOST
 F. gaditjirrii
 F. oxysporum 13307*LOST
F. newnesense 25184
 F. newnesense 66241
 F. hostae*LOST
+/+
 F. concolor 13459
 F. austroafricanum
 F. algeriense 66647
 F. algeriense 66648*LOST
 F. burgessii*LOST
 F. beomiforme
 F. babinda 53497
 F. babinda 25533
 F. babinda 25539
n103*LOST
 F. acuminatum CS5907
+/-
 F. acuminatum F829*LOST
 F. tricinctum 25481
 F. avenaceum Fa05001
 F. heterosporum
 F. graminum
 F. nurragi
n130*LOST
n123*LOST
nt/-
 F. albosuccineum
 F. decemcellulare

## Slide 6
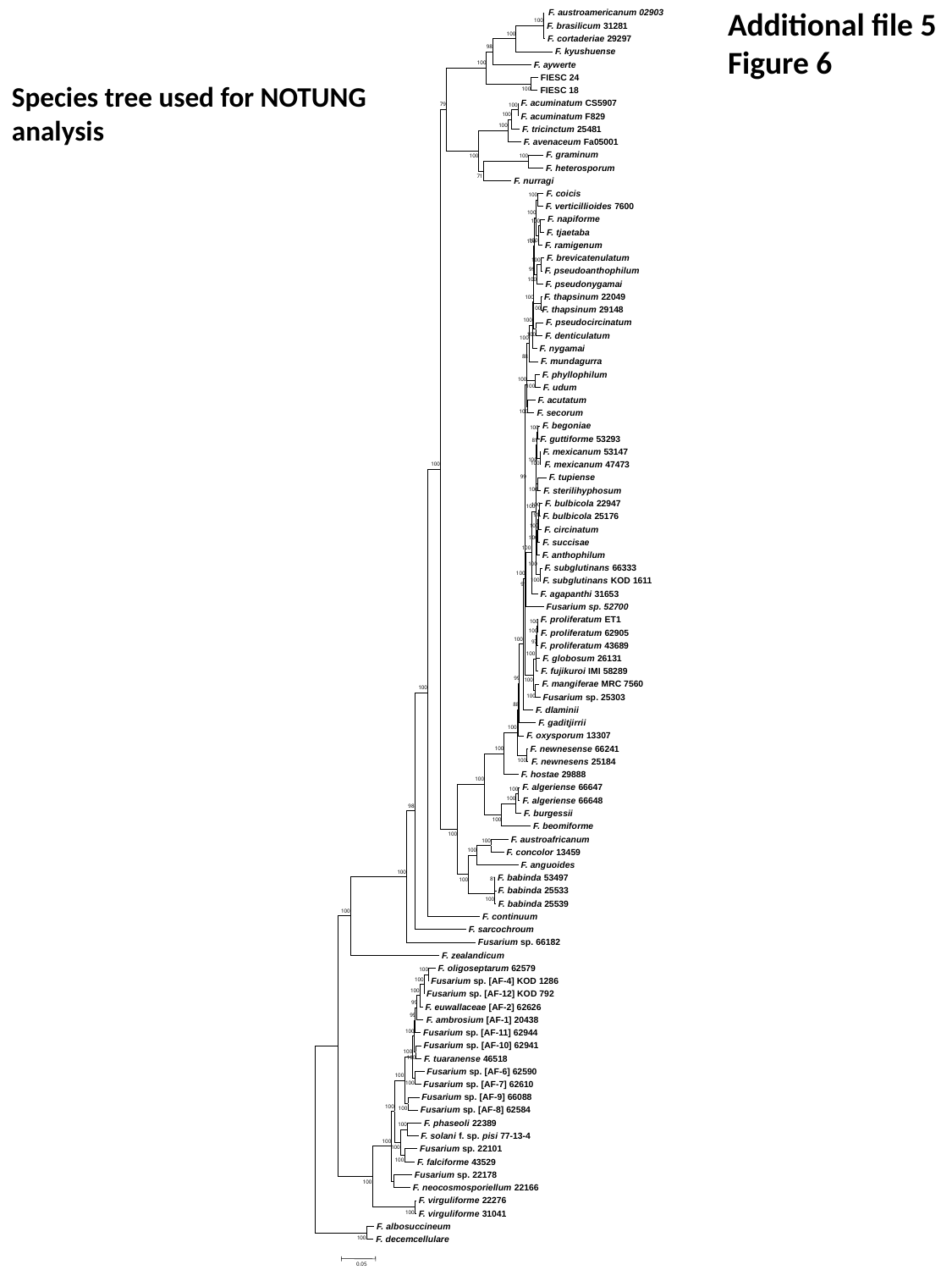

F. austroamericanum 02903
 F. brasilicum 31281
 F. cortaderiae 29297
 F. kyushuense
 F. aywerte
 FIESC 24
 FIESC 18
 F. acuminatum CS5907
 F. acuminatum F829
 F. tricinctum 25481
 F. avenaceum Fa05001
 F. graminum
 F. heterosporum
 F. nurragi
 F. coicis
 F. verticillioides 7600
 F. napiforme
 F. tjaetaba
 F. ramigenum
 F. brevicatenulatum
 F. pseudoanthophilum
 F. pseudonygamai
 F. thapsinum 22049
F. thapsinum 29148
 F. pseudocircinatum
 F. denticulatum
 F. nygamai
 F. mundagurra
 F. phyllophilum
 F. udum
 F. acutatum
 F. secorum
 F. begoniae
F. guttiforme 53293
 F. mexicanum 53147
 F. mexicanum 47473
 F. tupiense
 F. sterilihyphosum
 F. bulbicola 22947
 F. bulbicola 25176
 F. circinatum
 F. succisae
 F. anthophilum
 F. subglutinans 66333
 F. subglutinans KOD 1611
 F. agapanthi 31653
 Fusarium sp. 52700
 F. proliferatum ET1
 F. proliferatum 62905
 F. proliferatum 43689
 F. globosum 26131
 F. fujikuroi IMI 58289
 F. mangiferae MRC 7560
 Fusarium sp. 25303
 F. dlaminii
 F. gaditjirrii
 F. oxysporum 13307
 F. newnesense 66241
 F. newnesens 25184
 F. hostae 29888
 F. algeriense 66647
 F. algeriense 66648
 F. burgessii
 F. beomiforme
 F. austroafricanum
 F. concolor 13459
 F. anguoides
 F. babinda 53497
100
100
98
100
100
79
100
100
100
100
100
71
100
100
100
100
100
100
99
100
100
100
100
100
100
88
100
100
100
100
81
100
100
100
99
100
100
100
91
100
100
100
100
100
100
91
100
100
100
97
100
99
100
100
100
88
100
100
100
100
100
100
98
100
100
100
100
100
81
100
F. babinda 25533
100
 F. babinda 25539
100
 F. continuum
 F. sarcochroum
 Fusarium sp. 66182
 F. zealandicum
 F. oligoseptarum 62579
100
 Fusarium sp. [AF-4] KOD 1286
100
100
 Fusarium sp. [AF-12] KOD 792
99
 F. euwallaceae [AF-2] 62626
99
 F. ambrosium [AF-1] 20438
 Fusarium sp. [AF-11] 62944
100
 Fusarium sp. [AF-10] 62941
100
 F. tuaranense 46518
100
 Fusarium sp. [AF-6] 62590
100
 Fusarium sp. [AF-7] 62610
100
 Fusarium sp. [AF-9] 66088
100
 Fusarium sp. [AF-8] 62584
100
 F. phaseoli 22389
100
 F. solani f. sp. pisi 77-13-4
100
 Fusarium sp. 22101
100
 F. falciforme 43529
100
 Fusarium sp. 22178
100
 F. neocosmosporiellum 22166
 F. virguliforme 22276
 F. virguliforme 31041
100
 F. albosuccineum
 F. decemcellulare
100
0.05
Additional file 5 Figure 6
Species tree used for NOTUNG analysis
