# Supplementary material for: Identification and distribution of gene clusters required for synthesis of sphingolipid metabolism inhibitors in diverse species of the filamentous fungus Fusarium
Source: BMC Genomics. 2020 Jul 23;21:510. doi: 10.1186/s12864-020-06896-1 (PMC7376913; doi:10.1186/s12864-020-06896-1)
Supplement: Supplementary file 8 — Additional file 8. Deletion analysis of the SAM5 PKS gene, AOD1, in F. babinda NRRL 25539 and F. tricinctum strain NRRL 25481. [file 12864_2020_6896_MOESM8_ESM.docx]

**Additional file 8**

**
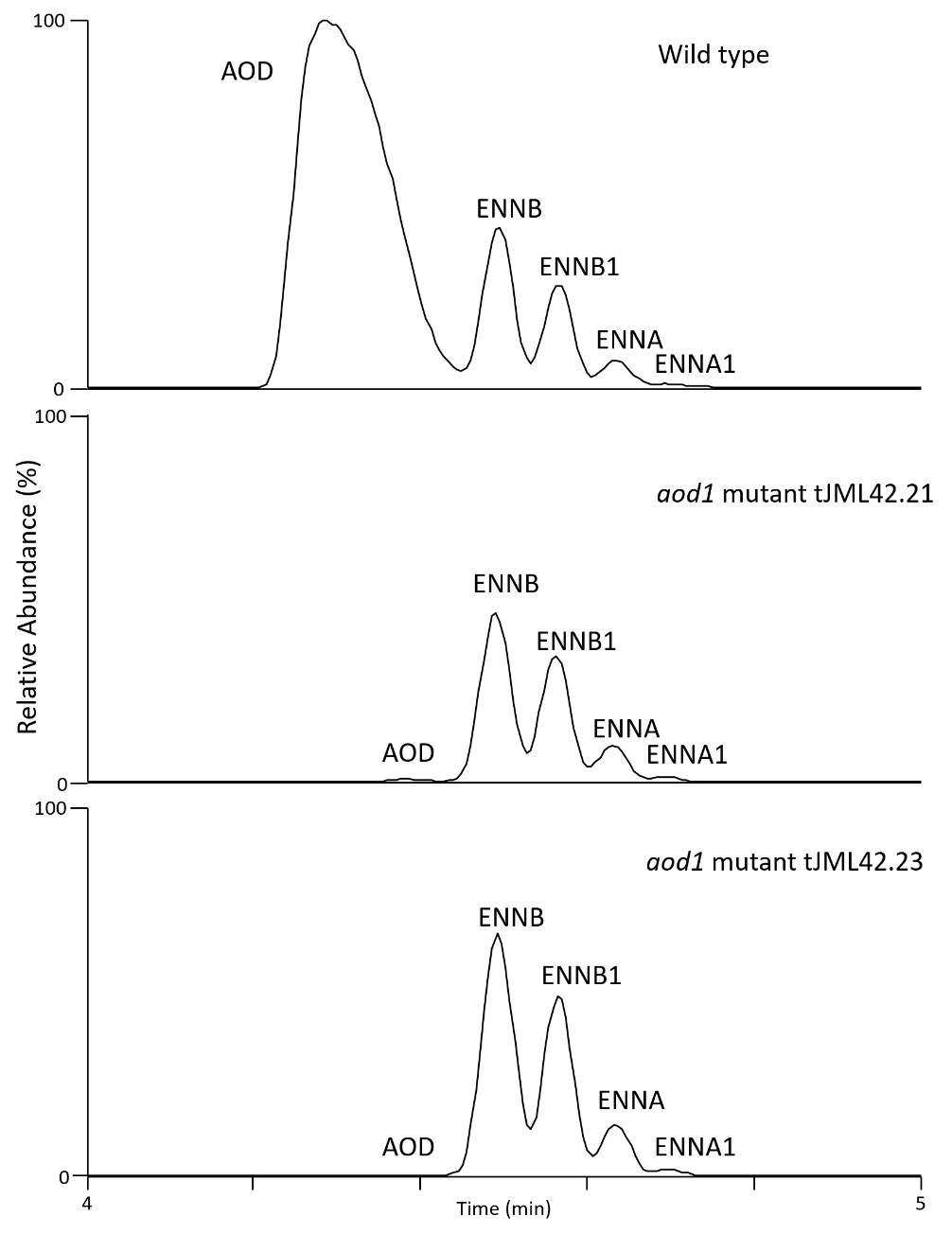
Additional file 8 - Figure 1:** Liquid chromatography-mass spectrometry analysis of AOD and enniatin production in the wild-type (NRRL 25481) and *aod1* mutant strains (tJML42.21 and tJML42.23) of *F. tricinctum*. The labels ENNA, ENNA1, ENNB and ENNB indicate peaks corresponding to four enniatin analogs.

**Additional file 8 – Table 1:** PCR primers used to prepare *AOD1* deletion constructs and for diagnostic PCR to detect *aod1* mutants.

| **Primer number** | **Sequence (5’ - 3’)** | **Primer used to:** |
| --- | --- | --- |
| **Primers for *AOD1* deletion constructs** | | |
| 2739  2740 | AAAAAAGGCGCGCCGGCCGCTCTAGAACTAGTGGATCCA  AAAAAAGGCGCGCCGAATTCCTGCAGCCCAACTGATATTG | amplify 1.5 kb *hygB* from plasmid pA-Hyg-OSCAR to construct plasmid pJML31.1 |
| 2820  2821 | CTCCAATCCTAACTCGCAAACAAGGC  GGCTGTTAGAGTCTGAAATTTGAAGAAGTGGT | amplify 1.5 kb *F. tricinctum* *AOD1* upstream fragment |
| 2822  2823 | ACCACTTCTTCAAATTTCAGACTCTAACAGCCGAATTCCTGCAGCCCAACTGATATTG  ACATCTTTGAGTAGTCTGATGATGATGTTAGTTTGGCCGCTCTAGAACTAGTGGATCCA | amplify 1.5 kb *hygB* fragment from pJML31.1 to generate *F. tricinctum* *AOD1* deletion construct |
| 2824  2825 | AAACTAACATCATCATCAGACTACTCAAAGATGT  ACACACATTGACCATTGTCTAGCTGTC | amplify 1.4 kb *F. tricinctum* *AOD1* downstream fragment |
| 2826  2827 | GCGTGGATCAACTCATCATAAACAACAGC  TTGACGAACCTCAATCGCTACCAATGA | amplify 3.9 kb *F. tricinctum* *AOD1* deletion construct |
| 2828  2829 | GTTCCCTCACGTATAAGCGATTGAGACT  GGCTGTTTAATGTTGGAGATGTGGAGATTG | amplify 1.5 kb *F. babinda* *AOD1* upstream fragment |
| 2830  2831 | CAATCTCCACATCTCCAACATTAAACAGCCGAATTCCTGCAGCCCAACTGATATTG  ATTTTCCAAAGACTATAGGTTAGGTTAAAGACTTGGCCGCTCTAGAACTAGTGGATCCA | amplify 1.5 kb *hygB* fragment from pJML31.1 to generate *F. babinda* *AOD1* deletion construct |
| 2832  2833 | AAGTCTTTAACCTAACCTATAGTCTTTGGAAAAT  GACAGAAGGCACAACGGTCTTTC | amplify 1.5 kb *F. babinda* *AOD1* downstream fragment |
| 2834  2835 | TCATCATACACGATGGCATCACCAGG  CAACCTGGCAAGAGACGGATGC | amplify 4.0 kb *F. babinda* *AOD1* deletion construct |
| **Primers for diagnostic PCR to detect *aod1* mutants** | | |
| 2757 | CCGATAGTGGAAACCGACGCC | detect juxtaposition of *hygB* and downstream fragment in *F. babinda aod1* mutant when paired with primer 2833 and in *F. tricinctum* *aod1* mutant when paired with primer 2825 |
| 2758 | CCAGCCAAGCCCAAAAAATGCTCC | detect juxtaposition of *hygB* and upstream fragment in *F. babinda aod1* mutant when paired with primer 2828 and in *F. tricinctum* *aod1* mutant when paired with primer 2820 |
| 2853  2854 | GCAGCCTGGAGTTGTTGTGG  CGTCGTCCAGTGCTTCAAGAGA | detect *F. tricinctum AOD1* |
| 2855  2856 | GCGAAGACTGGACCGAGATGTTTGC  AGAAGCGAGAGTGTCCACCAGG | detect *F. babinda AOD1* |

**
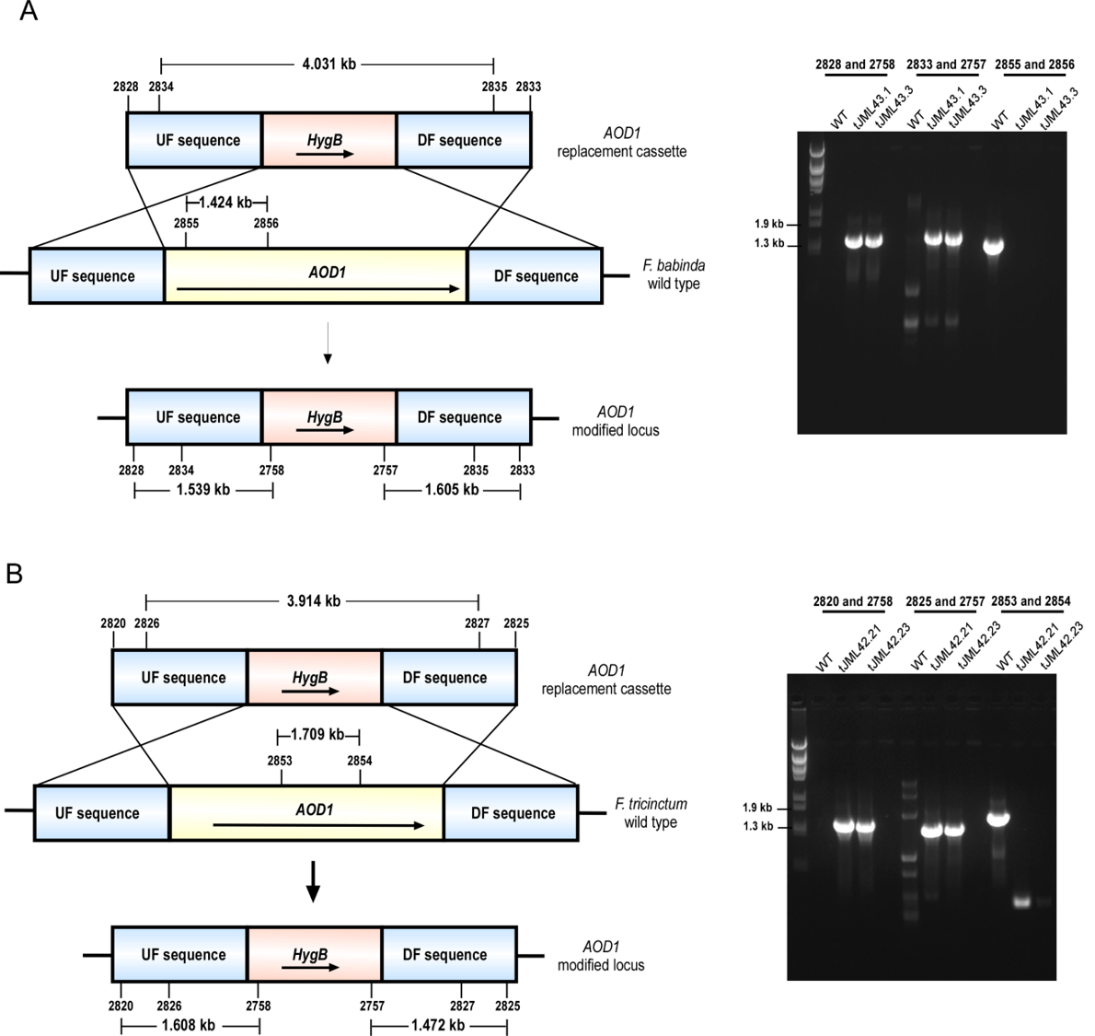
**

**Additional file 8 – Figure 2:** Construction and diagnostic PCR for deletion of *AOD1*. (A) *AOD1* deletion schematic in *F. babinda* strain NRRL 25539. To confirm *AOD1* deletion, diagnostic PCR assays were done with three primer combinations: i) primers 2828 and 2758 to amplify a 1.5 kb fragment from within *hygB* to 5’ of the upstream portion of the deletion cassette; ii) primers 2833 and 2757 to amplify a 1.6 kb fragment from within *hygB* to 3’ of the downstream portion of the deletion cassette; and iii) primers 2855 and 2856 to amplify a 1.4 kb fragment from within *AOD1*. The first two primer pairs were designed to yield an amplicon from *aod1* deletion mutants, but not from the wild type or transformants in which the deletion cassette integrated ectopically. The third primer pair was designed to yield an amplicon from the wild type and transformants in which the deletion cassette integrated ectopically, but not from *aod1* deletion mutants. (B) *AOD1* deletion schematic in *F. tricinctum* strain NRRL 25481. To confirm *AOD1* deletion, diagnostic PCR assays were done with three primer combinations: i) primers 2820 and 2758 to amplify a 1.6 kb fragment from within *hygB* to 5’ of the upstream portion of the deletion cassette; ii) primers 2825 and 2757 to amplify a 1.5 kb fragment from within *hygB* to 3’ of the downstream portion of the deletion cassette; and iii) primers 2853 and 2854 to amplify a 1.7 kb fragment from within *AOD1*. The first two primer pairs were designed to yield an amplicon from *aod1* deletion mutants, but not from the wild type or transformants in which the deletion cassette integrated ectopically. The third primer pair was designed to yield an amplicon from the wild type and transformants in which the deletion cassette integrated ectopically, but not from *aod1* deletion mutants. UF indicates upstream fragment and DF indicates downstream fragment as described in the Methods section.
