# Supplementary material for: Identification and distribution of gene clusters required for synthesis of sphingolipid metabolism inhibitors in diverse species of the filamentous fungus Fusarium
Source: BMC Genomics. 2020 Jul 23;21:510. doi: 10.1186/s12864-020-06896-1 (PMC7376913; doi:10.1186/s12864-020-06896-1)
Supplement: Supplementary file 9 — Additional file 9. Proposed sphingofungin C biosynthetic pathway inferred by comparison of the chemical structure of sphingofungin C and the content of enzyme-encoding genes in the putative sphingofungin biosynthetic gene cluster. Proposed functions of genes in the putative sphingofungin biosynthetic gene cluster based on sequence homology to genes of known function (Additional file 9 Table 1). Arrangement of genes in the putative sphingofungin biosynthetic gene cluster. Arrows represent genes and point in the direction of transcription. The designations for the 13 genes in the cluster are Afu3g14670 – Afu3g14770, Afu3g14790 and Afug14800. However, only the last three digits of these designations are shown above the genes in the figure. Genes encoding the PKS, AT and SDR enzymes are indicated (Additional file 9 Figure 1). Proposed sphingofungin C biosynthetic pathway (Additional file 9 Figure 2). [file 12864_2020_6896_MOESM9_ESM.docx]

**Additional file 9:**

**Proposed Sphingofungin Biosynthetic Pathway**

Here, we propose a biosynthetic pathway for the sphingofungin C, a SAM molecule produced by some isolates of *Aspergillus fumigatus*. The proposed pathway is based on our attempt to reconcile the predicted functions of genes in the putative sphingofungin biosynthetic gene cluster in *A. fumigatus* with the chemical structure of sphingofungin C [1, 2]. In addition to the PKS, AT and SDR, the putative sphingofungin cluster in *A. fumigatus* includes several other predicted enzyme-encoding genes (Additional file 9 Figure 1 and Table 1), and we propose that all of them play a role in synthesis of sphingofungin C. In the proposed pathway, the PKS (Afu3g14700) catalyzes formation of a 18-carbon linear and fully reduced polyketide; the AT (Afu3g14690) catalyzes condensation of the polyketide with serine to form a linear 20-carbon chain with an hydroxyl at C1, an amine at C2, and a carbonyl at C3; and the SDR (Afu3g14740) catalyzes reduction of the C3 carbonyl to a hydroxyl (Additional file 9 Figure 2). The presence of oxygen atoms at C4, C5 and C14 of the sphingofungin C structure indicates that three hydroxylation reactions likely occur at these positions during biosynthesis. The putative cluster includes only two classes of genes that are typically associated with hydroxylation reactions; one gene is predicted to encode a dioxygenase (Afu3g14710) and the other a cytochrome P450 monooxygenase (Afu3g14760). To account for the hydroxyl groups at C4, C5 and C14, either the dioxygenase or the monooxygenase would have to catalyze two hydroxylation reactions. There are examples of both classes of oxygenase enzymes that catalyze two hydroxylation reactions [3-5]. Thus, in the proposed pathway, either the dioxygenase or the monooxygenase catalyzes hydroxylation of two carbons (e.g., C4 and C5) of the sphingofungin backbone, and the other enzyme catalyzes hydroxylation of the other carbon (e.g., C14) (Additional file 9 Figure 2).

In the proposed sphingofungin C pathway, the formation of the acetyl group at C5 results from esterification of the C5 hydroxyl group. This reaction is proposed to be catalyzed by the acetyltransferase (Afu3g14730). The carboxyl group at C1 is proposed to result from two oxidation reactions of the C1 hydroxyl group that are catalyzed by the dehydrogenases Afu3g14790 and Afu3g14800. Formation of the carboxyl group from the hydroxyl group is analogous to the process that occurs during ethanol metabolism, where alcohol dehydrogenase catalyzes conversion of ethanol to acetaldehyde, and acetaldehyde dehydrogenase then catalyzes conversion of acetaldehyde to acetate. Finally, formation of the double bond at C6-C7 is proposed to result from reduction of the C6-C7 single bond catalyzed by reductase/dehydratase/epimerase Afu3g14770. Functional analyses are required to determine whether the proposed pathway is accurate and whether the enzymes function as predicted.

**Additional file 9 Table 1**: Proposed functions of genes in the putative sphingofungin biosynthetic gene cluster.

**Gene Model Predicted function based sequence predicted function in**

**homology sphingofungin biosynthesis**

Afu3g14670 Major Facilitator Superfamily Metabolite transport

Transporter

Afu3g14680 Lysophospholipase None predicted

Afu3g14690 Aminotransferase (AT) Condensation of polyketide

with serine (or alanine)

Afu3g14700 Polyketide synthase (PKS) Synthesis of linear 18-carbon

polyketide

Afu3g14710 Dioxygenase Hydroxylation of backbone at

C4, C5 and/or C14

Afu3g14720 Major Facilitator Superfamily Metabolite transport

Transporter

Afu3g14730 LysR family/Acyl transferase Acetylation of oxygen at C5

Afu3g14740 Short chain dehydrogenase/ 3-ketoreduction

Reductase (SDR)

Afu3g14750 Fungal specific transcription Regulation of gene transcription

factor domain

Afu3g14760 cytochrome P450 monooxygenase Hydroxylation of backbone at

C4, C5 and/or C14

Afu3g14770 dehydrogenase/reductase Formation C6-C7 double bond

Afu3g14790 Shikimate/quinate dehydrogenase Formation of C1 carboxyl

Afu3g14800 Shikimate/quinate dehydrogenase Formation of C1 carboxyl


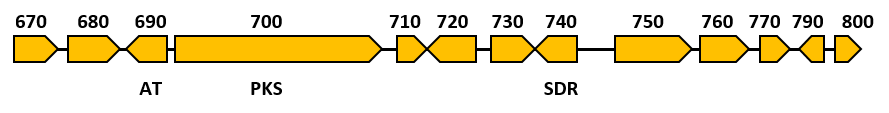


**Additional file 9 Figure 1:** Arrangement of genes in the putative sphingofungin biosynthetic gene cluster. Arrows represent genes and point in the direction of transcription. The designations for the 13 genes in the cluster are Afu3g14670 – Afu3g14770, Afu3g14790 and Afug14800. However, only the last three digits of these designations are shown above the genes in the figure. Genes encoding the PKS, AT and SDR enzymes are indicated.


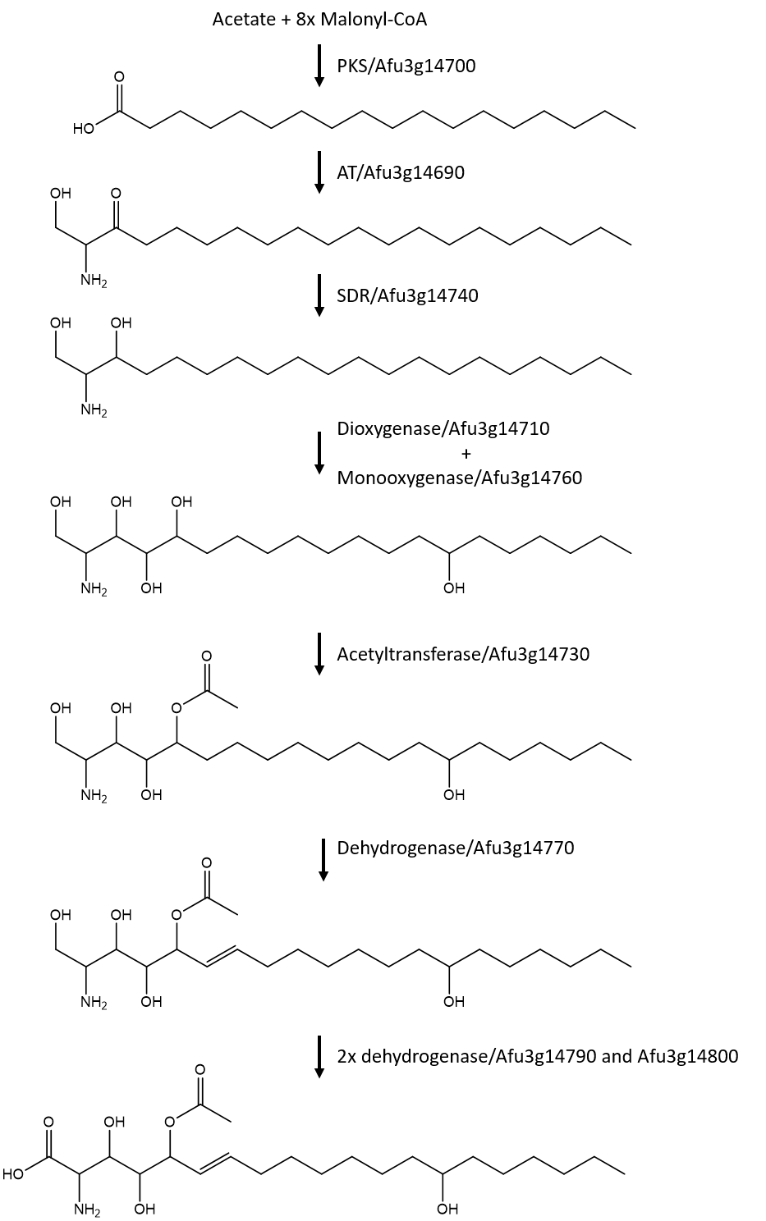


**Additional file 9 Figure 2:** Proposed sphingofungin C biosynthetic pathway.

**References**

1. Bignell E, Cairns TC, Throckmorton K, Nierman WC, Keller NP: Secondary metabolite arsenal of an opportunistic pathogenic fungus. *Philosophical transactions of the Royal Society of London Series B, Biological sciences* 2016, 371(1709).

2. Lind AL, Wisecaver JH, Lameiras C, Wiemann P, Palmer JM, Keller NP, Rodrigues F, Goldman GH, Rokas A: Drivers of genetic diversity in secondary metabolic gene clusters within a fungal species. *PLoS biology* 2017, 15(11):e2003583.

3. Cochrane RV, Vederas JC: Highly selective but multifunctional oxygenases in secondary metabolism. *Acc Chem Res* 2014, 47(10):3148-3161.

4. Bömke C, Tudzynski B: Diversity, regulation, and evolution of the gibberellin biosynthetic pathway in fungi compared to plants and bacteria. *Phytochemistry* 2009, 70:1876-1893.

5. Uhlig S, Busman M, Shane DS, Ronning H, Rise F, Proctor R: Identification of early fumonisin biosynthetic intermediates by inactivation of the *FUM6* gene in *Fusarium verticillioides*. *J Agric Food Chem* 2012, 60(41):10293-10301.
